# DNA framework signal amplification platform-based high-throughput systemic immune monitoring

**DOI:** 10.1038/s41392-024-01736-0

**Published:** 2024-02-07

**Authors:** Ye Chen, Xingyu Chen, Bowen Zhang, Yuxin Zhang, Songhang Li, Zhiqiang Liu, Yang Gao, Yuxuan Zhao, Lin Yan, Yi Li, Taoran Tian, Yunfeng Lin

**Affiliations:** 1 State Key Laboratory of Oral Diseases, National Center for Stomatology, National Clinical Research Center for Oral Diseases, West China Hospital of Stomatology, Sichuan University, Chengdu, 610041 Sichuan PR China; 2 Department of Prosthodontics, Tianjin Stomatological Hospital, School of Medicine, Nankai University, Tianjin, 300041 PR China; 3 Department of Laboratory Medicine, West China Hospital, Sichuan University, Chengdu, 610041 Sichuan PR China; 4 Sichuan Provincial Engineering Research Center of Oral Biomaterials, Chengdu, 610041 Sichuan China

**Keywords:** Nanobiotechnology, Prognostic markers

## Abstract

Systemic immune monitoring is a crucial clinical tool for disease early diagnosis, prognosis and treatment planning by quantitative analysis of immune cells. However, conventional immune monitoring using flow cytometry faces huge challenges in large-scale sample testing, especially in mass health screenings, because of time-consuming, technical-sensitive and high-cost features. However, the lack of high-performance detection platforms hinders the development of high-throughput immune monitoring technology. To address this bottleneck, we constructed a generally applicable DNA framework signal amplification platform (DSAP) based on post-systematic evolution of ligands by exponential enrichment and DNA tetrahedral framework-structured probe design to achieve high-sensitive detection for diverse immune cells, including CD4+, CD8+ T-lymphocytes, and monocytes (down to 1/100 μl). Based on this advanced detection platform, we present a novel high-throughput immune-cell phenotyping system, DSAP, achieving 30-min one-step immune-cell phenotyping without cell washing and subset analysis and showing comparable accuracy with flow cytometry while significantly reducing detection time and cost. As a proof-of-concept, DSAP demonstrates excellent diagnostic accuracy in immunodeficiency staging for 107 HIV patients (AUC > 0.97) within 30 min, which can be applied in HIV infection monitoring and screening. Therefore, we initially introduced promising DSAP to achieve high-throughput immune monitoring and open robust routes for point-of-care device development.

## Introduction

Systemic immune monitoring is an indispensable tool for early diagnosis of immunologic dysfunction while facilitating the real-time assessment of therapeutic outcomes.^1–3^ Further, it is critical for infection control, vaccine development, organ transplantation, and immunotherapy.^4–6^ Accordingly, immune monitoring has been widely confirmed as a fundamental healthcare evaluation method for immune status.^7^ Flow cytometry-based cell phenotyping is the primary method for conventional immune monitoring.^8,9^ However, as a one-by-one measurement, the widespread application of flow cytometry, especially in health screening and under-resourced regions, is hindered by its technical-sensitive, high-cost, and time-consuming nature.^10,11^ Moreover, the accuracy of flow cytometry in immune-cell phenotyping can be compromised owing to the antibody’s unspecific binding to the variants of immune markers.^12^ Several efforts have been made to improve instrument performance in fluidics and optics modules. However, the lack of intelligent and sensitive detection platforms is still the primary obstacle in the way of the development of high-throughput immune-cell phenotyping bioassay as the antibody-based labeling techniques limit its scalability.^13,14^ To overcome the challenge, some scholars introduced label-free Raman flow cytometry without cell labeling and washing. However, its clinical application is hindered by low throughput (1 events/s) caused by weak interaction between scattered light and target molecules and stronger fluorescence interference from the surrounding cellular environment.^15^

Hybridization chain reaction (HCR) is a potential strategy for constructing high-performance and generally applicable detection platforms. As an enzyme-free DNA self-assembly reaction, HCR forms extensive DNA duplex chains in response to external stimuli.^16,17^ Numerous researchers have engineered HCR-based aptasensing strategies by introducing aptamers into hairpin-structured aptamer probes’ toehold and stem regions to detect cellular membrane proteins. Nevertheless, the long detection time (>2 h), low sensitivity, and tedious manipulation (target cell isolation) cannot meet clinical demand.^18–21^ Wang et al. designed structure-switching aptamer probes to recognize membrane protein and trigger hybridization of two HCR probes. However, the detection time was long, reaching 4 h in several cases, and the intracellular uptake of probes became significant after 70 min, which can increase the unspecific fluorescence signal.^22^ Additionally, Chen et al. designed the aptamer recognition-promoted hybridization chain reaction system, including an aptamer probe and two HCR probes; however, the detection time was 2 h.^23^

Research on HCR probes for cell phenotyping in clinical sample testing has reached a bottleneck for two primary reasons.^24^ First, high-dissociation barriers between the aptamer and complementary strands can compromise the structure-switching kinetics of hairpin-structured aptamer probes.^25^ It was found that the accessibility of active domains played a crucial role in switching kinetics. Dissociation has rarely been observed when complementary strands lock the active domains.^25^ However, due to limited protein-nucleic acid interaction analyses, identifying the active domains in most aptamers remains challenging. Second, the reaction between hairpin1 and hairpin2 is often sluggish due to random collisions in the solution and charged repulsion between the cell membranes and hairpins, which hampers detection sensitivity.^26^ Researchers have tried to anchor HCR probes onto Au nanoparticles and graphene oxide surfaces to enhance the local probe concentration. However, achieving precise control over probe addressability, density, and orientation on these scaffolds remains challenging.^27,28^

**Fig. 1:**
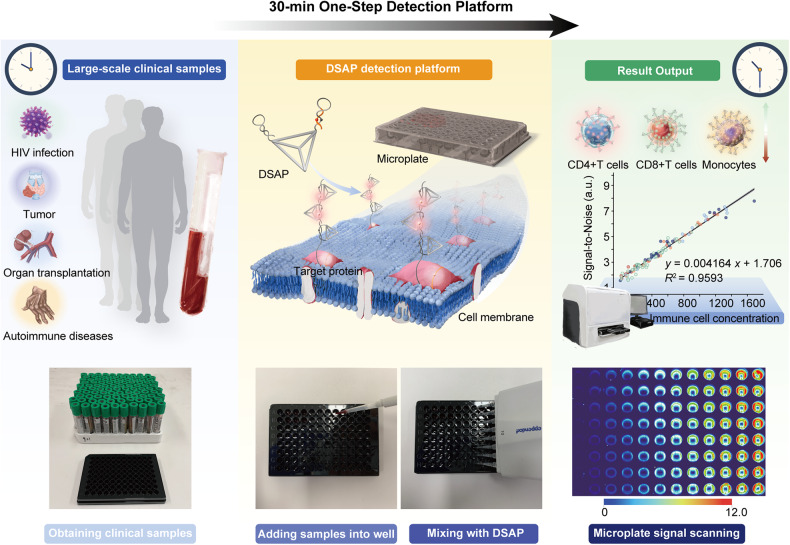
Schematic illustration of DSAP-based high-throughput immune monitoring. The wide application of immune monitoring in diverse diseases brings a huge burden for clinical detection. DSAP is a signal switching-off/on highly-sensitive detection platform for immune cells. It simplifies the clinical procedure to only one step and shortens detection time significantly. After obtaining a batch of samples, adding 100 μl blood samples into a microwell and mixed with 20 μl DSAP. Diverse immune cell numbers can be easily calculated according to signal-to-noise. The entire waiting time is no longer than 30 min. Therefore, large-scale samples can be rapidly detected in a very short time. The fluorescence visualization of microplates during DASP-based immune monitoring. The scale of the thermal figure is shown on the left

This work proposed a DNA framework signal amplification platform (DSAP) construction strategy by post-SELEX (Systematic Evolution of Ligands by Exponential Enrichment) aptamer optimization and DNA tetrahedral framework (DTF)-structured HCR probe design. First, the active domains were isolated based on post-SELEX optimization, and the partial active domains were loaded into the toehold regions of hairpin1 with the sticky end (H1-SE), which accelerated their dissociation from complementary strands upon binding to target proteins. Besides, DTF has gained increasing interest owing to its one-pot assembly, simple design, and high-yield attributes.^29,30^ DTF was used as a scaffold for probes, enabling precise spatial orientation and distance control at the nanometric scale.^31^ The spatial confinement of DTF can improve the local concentration of hairpins by 578 folds, which increases sensitivity by 40 folds. Based on the generally applicable DSAP, we developed a high-throughput immune-cell phenotyping system by quantitatively analyzing immune cells, as we showed for CD4+ T, CD8+ T lymphocytes, and monocytes. DSAP reduced the time required for the immune-cell phenotyping of clinical blood samples to 30 min without cell washing and manual subset analysis (Fig. 1). The results obtained using the DSAP were highly consistent with those obtained using conventional flow cytometry. As a proof-of-concept, DSAP demonstrated high accuracy in staging human immunodeficiency virus (HIV) infection and immune monitoring in cancer treatment. Therefore, our proposed DSAP is a promising method for rapid clinical immune monitoring and boosting the research of portable point-of-care devices.

## Results and discussion

### Post-SELEX optimization of immune-cell aptamers

SELEX serves as a standard procedure for screening aptamers, producing aptamer sequences of 80 ~ 120 nucleotides (nt) in length.^32,33^ However, several unfunctional regions may be present in the screened sequences that do not have any affinity to target proteins. The unfunctional regions may show less affinity owing to increased steric hindrance^34^ and impede the design of the aptamer-based HCR probes, adversely affecting the detection sensitivity. Therefore, this study employed a structure-guided post-SELEX optimization strategy to truncate unfunctional regions in the screened aptamers sequence and obtain the active domains, which contribute to the primary affinity to target proteins. The detailed method of aptamer truncation, secondary structure prediction, and interaction simulation can be observed in Supplementary Figs. S1–S5.

**Fig. 2:**
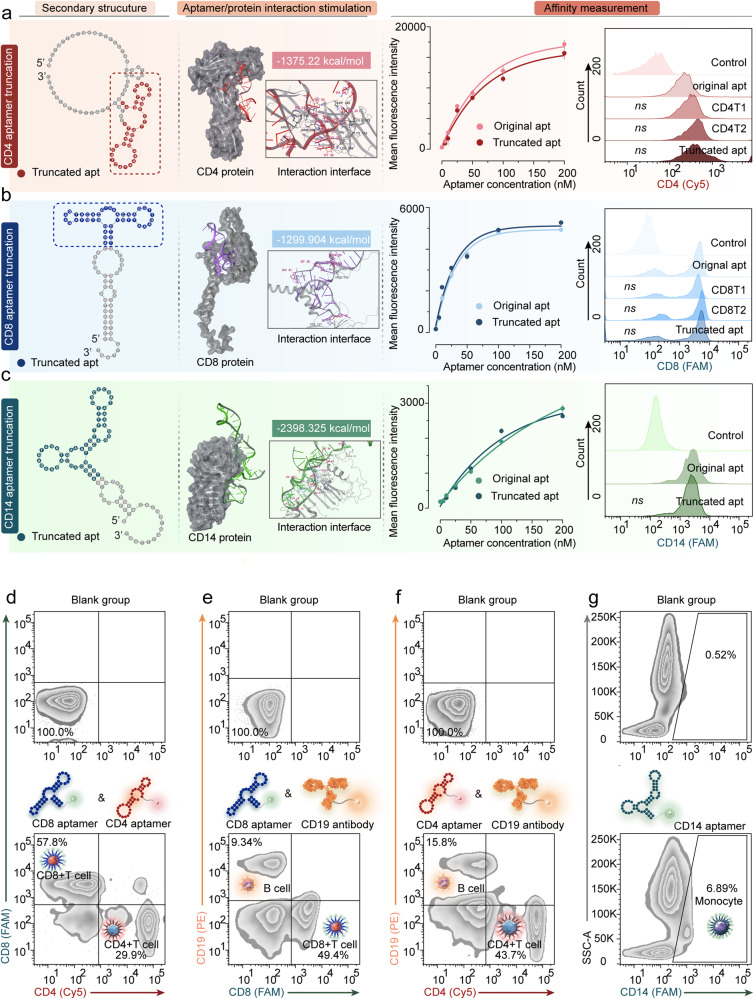
The structure-guided post-SELEX optimization of sensitive and specific aptamers of CD4+, CD8+ T lymphocytes, and monocytes. **a** The structure-guided post-SELEX optimization of CD4. The secondary structures of CD4 aptamers were predicted by Mfold software. The gray regions represent unfunctional regions in original aptamers. The colorized regions represent truncated aptamer sequences. The aptamer/protein interaction simulations were conducted for CD4 aptamer with human CD4 protein (PDB ID: 7T0R Chain C). The 3D model, PIPER pose energy, and interaction interfaces were displayed. The affinity measurements of the aptamers for detecting CD4+ T lymphocytes were carried out by flow cytometry. The dissociation curve of original aptamers and truncated aptamers was shown. The histogram of the original aptamer, CD4 T1, CD4 T2, and truncated aptamer is displayed. **b** The structure-guided post-SELEX optimization of CD8. The secondary structure of aptamer before and after truncation, the interaction simulation results, and affinity measurement were shown. **c** The structure-guided post-SELEX optimization of CD14. The secondary structure of aptamer before and after truncation, the interaction simulation results, and affinity measurement were shown. **d** The FCA of whole blood cells before and after being treated with Cy5-labeled CD4 aptamer and FAM-labeled CD8 aptamer. The CD8+/CD4− cells represent mature CD8+ T lymphocytes. The CD4+/CD8− cells belong to mature CD4+ T lymphocytes. **e** The FCA of whole blood cells before and after being treated with FAM-labeled CD8 aptamer and PE-labeled CD19 antibody. The CD8+/CD19− cells are CD8+ T lymphocytes and the CD19+/CD8− cells represent B cells. **f** The FCA of whole blood cells before and after being treated with Cy5-labeled CD4 aptamer and PE-labeled CD19 antibody. The CD4+/CD19− cells represent CD4+ T lymphocytes. The CD19+/CD4− cells belong to B cells. **g** The FCA of whole blood cells before and after being treated with FAM-labeled monocyte aptamer. The gated cells are monocytes. The error bars in (**a**) are determined by the standard deviation (SD) of the MFI from three parallel experiments. All the tested samples were technical replicates. The abbreviation ‘apt’ in a means aptamer

First, in the previous reports, we chose human CD4 protein aptamer, human CD8 protein aptamer, and human CD14 protein aptamer, which showed high affinity to the target protein.^35–37^ The aptamer truncation was conducted based on the secondary structure prediction, trial-and-error testing, and DNA/protein interaction simulation. Mfold software provided the secondary structure of the aptamers. The flow-cytometric analysis (FCA) evaluated the aptamer affinity changes. The interaction simulation predicted the binding stability and binding sites between proteins and aptamers by Maestro software. For CD4 aptamer truncation, CD4 T1 was acquired by truncating the 3’ primer of the original aptamer. CD4 T2 was acquired by truncating the 5’ primer of CD4 T1. The truncated aptamer was produced by truncating a free single-strand from CD4 T2. The stem-loop structures of the aptamer usually provide the primary affinity. The secondary structure of the truncation aptamer was conserved in those of the original aptamer: CD4 T1 and CD4 T2. According to FCA, the mean fluorescence intensity (MFI) of CD4+ T lymphocytes demonstrated no significant difference after treatment with these aptamers (Fig. 2a and Supplementary Fig. S8). We further extracted the two stem-loop to form CD4 T3 and CD4 T4. However, CD4 T3 and CD4 T4 exhibited no affinity to CD4+ T lymphocytes. Therefore, the truncated aptamer is the active domain of the original aptamer (Supplementary Fig. S1). We conducted CD4 truncated aptamer/CD4 protein interaction simulation. The PIPER pose energy was −1375.22 kcal/mol, indicating strong binding stability. The interaction sites between aptamer and protein are provided in Supplementary Figs. S4 and S5). The dissociation curve and *K*_D_ values evaluate the affinity of the original and truncated aptamer. The *K*_D_ value of the truncated aptamer was 41.63 nM, which is lower than that of the original aptamer (47.53 nM, Fig. 2a).

For CD8 aptamer truncation, CD8 T1 was produced by truncating the original aptamer’s 5’ and 3’ primer. CD8 T2 was acquired by truncating the free single strand from CD8 T1. The truncated aptamer was obtained by truncating the partial stem sequence. The secondary structure of the truncation aptamer was conserved in those of the original aptamer: CD8 T1 and CD8 T2. According to FCA, the MFI of CD8+ T lymphocytes treated with these aptamers exhibited no significant difference (Fig. 2b). We further truncated the partial stem sequences to obtain CD8 T3; however, the stem could not be maintained, leading to the larger secondary structure difference from the truncated aptamer. FCA showed that CD8 T3 had a weaker affinity to CD8+ T lymphocytes. Therefore, the truncated aptamer is the active domain of the original aptamer (Supplementary Fig. S2). The PIPER pose energy between CD8 truncated aptamer and CD8 protein was −1299.904 kcal/mol (Supplementary Figs. S4 and S5). According to FCA, the *K*_D_ value of the truncated aptamer was 22.01 nM, slightly lower than that of the original aptamer (22.41 nM).

For CD14 aptamer truncation, the truncated aptamer was produced by truncating partial stem-loop regions. According to FCA, the MFI of monocytes treated with CD14 truncated aptamer showed no difference from the MFI of monocytes treated with the original aptamer (Fig. 2c). We further truncated the partial stem regions in the end to obtain CD14 T2. The partial stem-loop structure of the truncated aptamer was extracted to form CD14 T3 and CD14 T4. However, CD14 T2, CD14 T3, and CD14 T4 showed no affinity to monocytes. Therefore, the truncated aptamer was the primary active domain (Supplementary Fig. S3). Further truncating the truncated aptamer can decrease its affinity. According to interaction simulation, the PIPER pose energy was −2398.325 kcal/mol. The *K*_D_ value of the truncated aptamer was 51.08 nM, slightly lower than that of the original aptamer (141.2 nM, Supplementary Figs. S4 and S5).

The interaction between the target protein and aptamer was validated by the electrophoretic mobility shift assay (EMSA). The sensitivity and specificity of truncated and original aptamers were also explored by EMSA. The *K*_D_ value decided by EMSA of the truncated aptamer was similar to or lower than that of the original aptamer. However, the *K*_D_ values are not absolutely equal to those calculated by cell detection, especially for that of CD8 original aptamer. Inconsistent structures of recombinant proteins may be the cause of this issue when compared to the actual protein structures (Supplementary Fig. S6). The truncation did not have any influence on the specificity of aptamer (Supplementary Fig. S7). Besides, to explore the detection capacity of truncated aptamer in whole blood samples, the blood samples were treated with Cy5-labeled CD4 truncated aptamer, FAM-labeled CD8 truncated aptamer, or PE-labeled CD19 antibodies at 25 °C for 30 min. FCA revealed distinct subsets of Cy5+/FAM− and Cy5−/FAM+ cells in lymphocytes, showing no PE signal (Fig. 2d–f). Neutrophils and monocytes hardly showed any FAM signal. The blood samples were treated with FAM-labeled CD14 truncated aptamer at 25 °C for 30 min. According to FCA, the FAM-labeled CD14 truncated aptamer demonstrated high affinity and specificity toward monocytes. Neutrophils and lymphocytes hardly exhibited FAM signaling (Fig. 2g). These findings highlight the remarkable sensitivity and specificity of the three aptamers for whole blood detection.

Furthermore, we applied the truncated aptamer-based and fluorescence antibody-based methods to detect CD4+, CD8+ T lymphocytes, and monocytes in 30 blood samples. FCA can calculate the concentration of detected CD4+, CD8+ T lymphocytes, and monocytes by truncated aptamer and antibody. We analyzed the consistency between the two methods when deciding the target cell concentration. A good linear relationship between the two methods was obtained (Supplementary Figs. S9–S12).

### Fabrication and characterization of DSAP

**Fig. 3:**
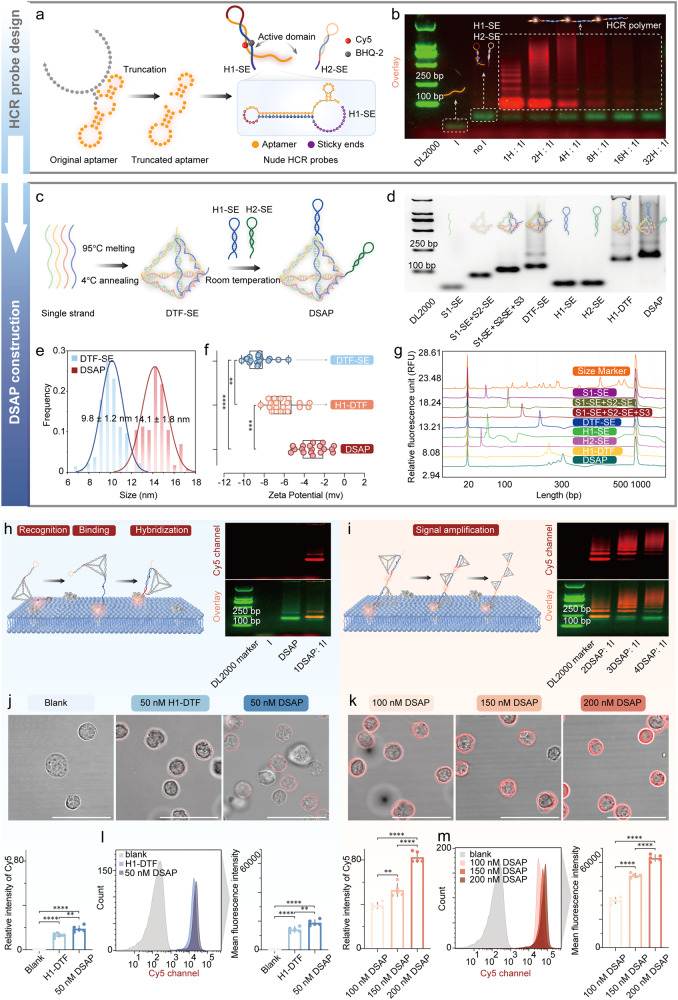
Design, fabrication, characterization, and functional verification of DSAP. **a** The fabrication of aptamer-based HCR probes. The truncated aptamer was embedded into the toehold and stem regions of H1-SE. The Cy5 and BHQ-2 were modified onto the bases in the stem regions. **b** The agarose gel electrophoresis (AGE) shows the fluorescence intensity and molecular weight changes of the reaction product between the HCR probe and initiator of varying molar ratios. The gel image is overlaid by GelRed channel (green) and Cy5 channel (red). The first panel is the DL2000 ladder, the second panel represents the initiator, the second panel is the mixture of H1-SE and H2-SE without an initiator, the fourth to the ninth panel is the reaction product between HCR probes (H1-SE, H2-SE) and the initiator of varying molar ratios. **c** The synthesis procedures of the DSAP. The DTF-SE was synthesized by four DNA oligos. The H1-SE and H2-SE were connected with DTF-SE via sticky ends. **d** The AGE shows the step-wise construction of the DSAP. **e** The particle size of DTF-SE and DSAP calculated by TEM images. Data are presented as the mean ± SD (*n* = 4). **f** The statistical diagram of the zeta potential of DTF-SE, Hairpin1-DTF (H1-DTF), and DSAP. **g** The capillary electrophoresis results show the step-wise construction of the DSAP. **h**, **i** The scheme and AGE showing the mechanism of the membrane protein recognition, binding, hairpin hybridization, and signal amplification by DSAP. **j**, **k** The CLSM characterized the signal amplification capacity of DSAP when detecting CD4+ T lymphocytes. As the CLSM shows, as the concentration of DSAP increased, the membrane fluorescence intensity became stronger. The histogram showed fluorescence intensity from three parallel experiments. **l**, **m** The FCA also displayed the Cy5 signal changes as the increased concentration of DSAP. The “H” represents HCR probes, including H1-SE and H2-SE, and the “I” represents the initiator. The **** represented *p* < 0.0001, *** represented *p* < 0.001. ** represented *p* < 0.01. Scale bars, 50 μm

The fabrication of DSAP required two steps: nude HCR probe design and DTF-structured probe design. First, we fabricated hairpin1 with the sticky end (H1-SE) and hairpin2 with the 3’ sticky end (H2-SE). The truncated aptamers were embedded into the toehold, stem, and a part of the loop region of H1-SE (the yellow region, Fig. 3a and Supplementary Table 1). To lower the dissociation barrier of H1-SE, the toehold region contained partial interaction sites of the truncated aptamer confirmed by interaction simulation. The sticky ends of H1-SE and H2-SE were reverse complementary to the sticky end of S1(S1-SE) and the sticky end of S2 (S2-SE), respectively. The residual sequences of H1-SE and H2-SE were ensured according to the HCR design principle proposed by Dirks and Pierce.^38^ To enable off/on signal switching, a pair of fluorophore/quenchers (e.g., Cy5/BHQ-2 or FAM/BHQ-1) was attached to opposite bases in the stem region (Supplementary Fig. S13). Without target proteins, H1-SE and H2-SE can remain metastable because of the complementary pairing regions in the hairpin stem, and fluorescence is quenched due to the close distance between fluorophore and quencher (Supplementary Fig. S14). The binding of the aptamers with target proteins can induce dissociation of H1-SE that enables unlocked H1-SE to hybridize with hairpin2 via the strand displacement reaction, further initiating the HCR. The cascade amplification reaction of H1-SE and H2-SE can trigger fluorescence amplification. The fluorescence intensity was positively relative to the concentration of the target. The H1-SE and H2-SE reacted with the diluted initiator strand (the reverse complementary base pairing sequence of aptamer) of different concentrations (1:1, 2:1, 4:1, 8:1, 16:1 and 32:1 molar ratio of DSAP to initiator). The agarose gel electrophoresis was subsequently performed, and the bands of the high-concentration group were observed to be lighter than those of the low-concentration group (Fig. 3b).

Four individual DNA strands, including S1-SE, S2-SE, S3, and S4, underwent 95 °C denaturation and 4 °C annealing for self-assembly, ultimately forming DTFs with sticky ends (DTF-SE). DSAP was developed by immobilizing H1-SE and H2-SE onto the two vertices of DTF-SE by base complementary pairing for 10 min at 25 °C (Fig. 3a and Supplementary Fig. S15). Due to the spatial confinement of DTF, the distance between H1-SE and H2-SE in the DSAP was ~11 nm (including DTF skeleton and sticky ends). Based on collision theory, the local hairpin concentration of the DSAP was 115.7 µM, 578 times higher than that of single-dispersed nude HCR probes (200 nM total concentration). Upon binding the aptamer to the target protein and subsequently unlocking hairpin1, the adjacent hairpin2 instantly hybridized with hairpin1 via strand displacement reaction, further driving the cascade polymerization of more DSAP to achieve signal amplification, which significantly improved the HCR speed (Supplementary Figs. S16 and S17). The step-wise synthesis of DSAP was determined by agarose gel electrophoresis (AGE, Fig. 3d), capillary electrophoresis (Fig. 3g), and 8% polyacrylamide gel electrophoresis (PAGE,^35^ Supplementary Fig. S18). Transmission electron microscopy (TEM) was employed to examine the morphologies of DTF-SE and DSAP. The mean particle sizes of DTF-SE and DSAP were calculated as ~9.9 ± 1.2 and 14.1 ± 1.8 nm according to TEM images (Fig. 3e and Supplementary Fig. S19). The charge levels of DTF-SE, H1-DTF, and DSAP were assessed using zeta potential measurements, and the mean charges of DTF-SE, H1-DTF, and DSAP were −3.1, −6.8, and −9.1, respectively (Fig. 3f).

To characterize the signal amplification property of DSAP, we first validated the signal amplification using the HCR initiator, which is the reverse complementary sequence of the CD4 aptamer. CD4 DSAP was selected as an example. When DSAP was treated with initiators at a 1:1 molar ratio, H1-SE and H2-SE on DSAP hybridized. When DSAP was treated with initiators at higher molar ratios, the assembly of DSAP formed HCR polymers of larger molecular weight (Fig. 3h, i). Next, we validated signal amplification in cell detection. H1-DTF yielded a *K*_D_ of 24.34 nM in CD4+ T lymphocyte detection (Supplementary Fig. S20), indicating that the binding to CD4+ T lymphocytes by DSAP achieved saturation at 50 nM DSAP. We treated CD4+ T lymphocytes with 50 nM H1-DTF, 50 nM DSAP, 100 nM DSAP, 150 nM DSAP, and 200 nM DSAP to validate signal amplification. According to the reaction mechanism, the duplex, triplex, and quadruplex DSAP could be formed in 100, 150, and 200 nM DSAP-treated cells, respectively. According to confocal laser scanning microscopy (CLSM), the Cy5 MFI of the 200 nM DSAP treated cells was approximately four times higher than 50 nM H1-DTF treated cells, three times higher than the 50 nM DSAP treated cells, two times higher than the 100 nM cells, and 1.3 times higher than the 150 nM cells (Fig. 3j, k). FCA also demonstrated that as DSAP concentration increased, the cascade amplification increased the MFI of cells (Fig. 3l, m).

**Fig. 4:**
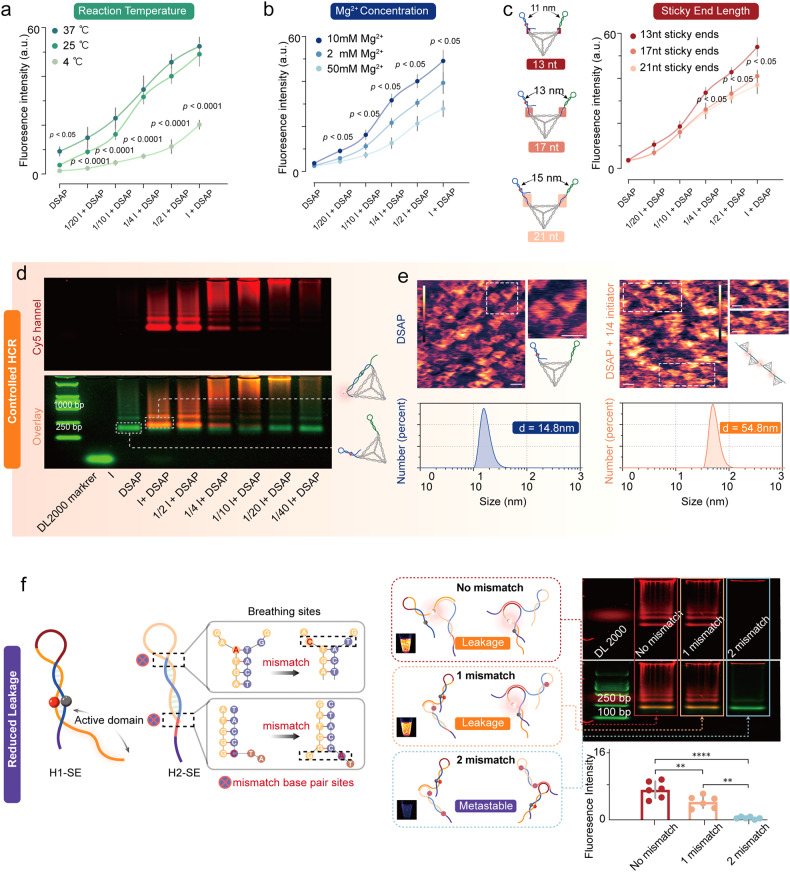
The conditional and structural optimization of the HCR efficiency of DSAP. **a** The influence of reaction temperature on the HCR efficiency of the DSAP, including 4, 25, and 37 °C. **b** The influence of Mg^2+^ concentrations in the reaction system on the HCR efficiency of the DSAP, including 2 mM Mg^2+^, 10 mM Mg^2+,^ and 50 mM Mg^2+^. **c** The influence of sticky end length on HCR efficiency of DSAP, including 13, 17, and 21 nt. The error bars in (**a**–**c**) are SD according to three repetitive experiments. **d** The AGE showing the initiator, DSAP, and the reaction products between initiators and DSAP of different ratios. The red bands are generated by the Cy5 channel, and the green bands are generated by the GelRed channel. As the Figure shows, the initiator of lower concentration can trigger larger DNA polymers, but the initiator of higher concentration can trigger smaller DNA polymers. **e** The morphology and particle sizes of the DSAP and HCR reaction products between DSAP and initiator at the molar ratios of 4:1 were determined by AFM imaging and particle size analysis. The mean particle size is decided by three parallel measurements. **f** The breathing sites can trigger self-hybridization of H1-SE and H2-SE. The base pair mismatches in the breathing sites of H2-SE can improve the metastability of DSAP. The visualization of the DSAP with no mismatch, one mismatch, and two mismatches was displayed. The AGE and fluorescence intensity measurements were also shown for the corresponding group. The error bars in (**a**–**f**) are determined by the SD of the MFI from at least five parallel experiments. All the tested samples were technical replicates. The “I” represents the initiator. The **** represented *p* < 0.0001, ** represented *p* < 0.01. Scale bars, 10 nm

Next, we explored the influence of reaction temperature, magnesium ion (Mg2+) concentration, and sticky end length on the HCR efficiency of DSAP. First, DSAP was treated with diluted initiators (molar ratios between DSAP and initiator of 20:1, 10:1, 5:1, 2:1, 1:1) at 4, 25, and 37 °C. We found that higher reaction efficiency occurred at 25 and 37 °C than at 4 °C (*p* < 0.0001). No significant difference was shown between the 25 and 37 °C groups; however, larger background noise was shown in the 37 °C group (*p* < 0.05, Fig. 4a). Besides, we compared the HCR efficiency of DSAP in detecting CD4+ T lymphocytes at 4, 25, and 37 °C. FCA showed that a temperature of 25 °C facilitated HCR (vs. 37 °C, *p* < 0.05; vs. 25 °C, *p* < 0.0001, Supplementary Fig. S21). Therefore, we chose 25 °C as the reaction temperature for the following experiments due to higher signal-to-noise. Subsequently, we explored the optimal Mg2+ concentration for the HCR of DSAP. DSAP was treated with diluted initiators of various concentrations in TM buffers containing 2, 10, and 50 mM Mg2+. We found that 10 mM Mg2+ facilitated the HCR of DSAP the most (vs. 50 mM Mg2+, *p* < 0.05). The 50 mM Mg2+ concentration limited the structural switching of H1-SE, whereas 2 mM Mg2+ may have influenced strand propagation (Fig. 4b). The spatial confinement effect of the DTFs facilitated the alignments of H1-SE and H2-SE in close proximity. The distance between H1-SE and H2-SE can be adjusted by the sticky end length, about 11, 13, and 15 nm for DSAP with 13-nt, 17-nt, and 21-nt sticky ends, respectively. The DSAP with 13-nt SE demonstrated the highest HCR efficiency (vs. 17-nt or 21-nt SE, *p* < 0.05, Fig. 4c). However, our preliminary experiments revealed that sticky ends shorter than 13 nt could reduce HCR efficiency due to the limited flexibility of the hairpins.

To quantify the immune cells, a controllable HCR was needed to ensure relatively consistent fluorescence intensity among the target cells. First, an adequate DSAP concentration needed to be verified. As indicated by the AGE results, the DSAP treated with a less concentrated initiator produced larger HCR polymers, whereas the highly concentrated initiator produced smaller HCR polymers (Fig. 4d). The primary reaction products between DSAP and the initiator at a molar concentration ratio of 4:1 were three times larger in molecular weight than DSAP. The AFM image showed the single DSAP without an initiator. When DSAP was treated with the initiator at a molar concentration of 4:1, the quadruplex DSAP structures were mainly observed. According to particle size measurement, the particle size was ~54.8 nm, nearly four times larger than DTF-SE (*p* < 0.0001, Fig. 4e). Besides, according to AGE, strand propagation of DSAP decreased because of increased steric hindrance at higher DSAP-to-initiator ratios (>4:1), consistent with reported work (Supplementary Fig. S22).^16,39^ Second, the kinetics of aptamer recognition were relatively slower than that of strand displacement. This difference in kinetics ensures that DSAP cannot initiate signal amplification until they bind with the target proteins. Therefore, DSAP amplification can be controlled by the two aspects. In immune-cell detection, according to the above-mentioned experiment results, 200 nM DSAP produced fluorescence on cell membranes that were three times stronger than 50 nM DSAP (Fig. 3j–m). Thus, the 200 nM concentration of DSAP can contribute to the form of quadruplex DSAP on cell membranes, which was used in subsequent experiments.

Background leakage of the HCR probe can lead to high detection background noise, which is caused by cross-linking H1-SE and H2-SE without the target. The binding of the toehold with the protein contributes to the H1-SE dissociation. Thus, it is necessary for the toehold to incorporate interaction sites, according to interaction simulation, which can lead to background leakage due to long toehold regions. To reduce background noise leakage, we investigated whether a mismatch in the breathing sites of H2-SE could enhance the metastability of HCR probes. Based on the results obtained from both AGE and microwell fluorescence analyses, the incorporation of two mismatches located in the two breathing sites led to a significant enhancement in metastability and a 14-fold reduction in background noise when compared with the one mismatch (*p* < 0.01) and no mismatch cases (*p* < 0.0001, Fig. 4f). Notably, several scholars shortened the length of loop regions in HCR probes to reduce background leakage; however, this method can decrease the stored energy in the hairpin structure, which can harm HCR efficiency. By contrast, the mismatches in H2-SE did not have any negative influence on the efficiency of HCR.

### Excellent detection performance of DSAP

**Fig. 5:**
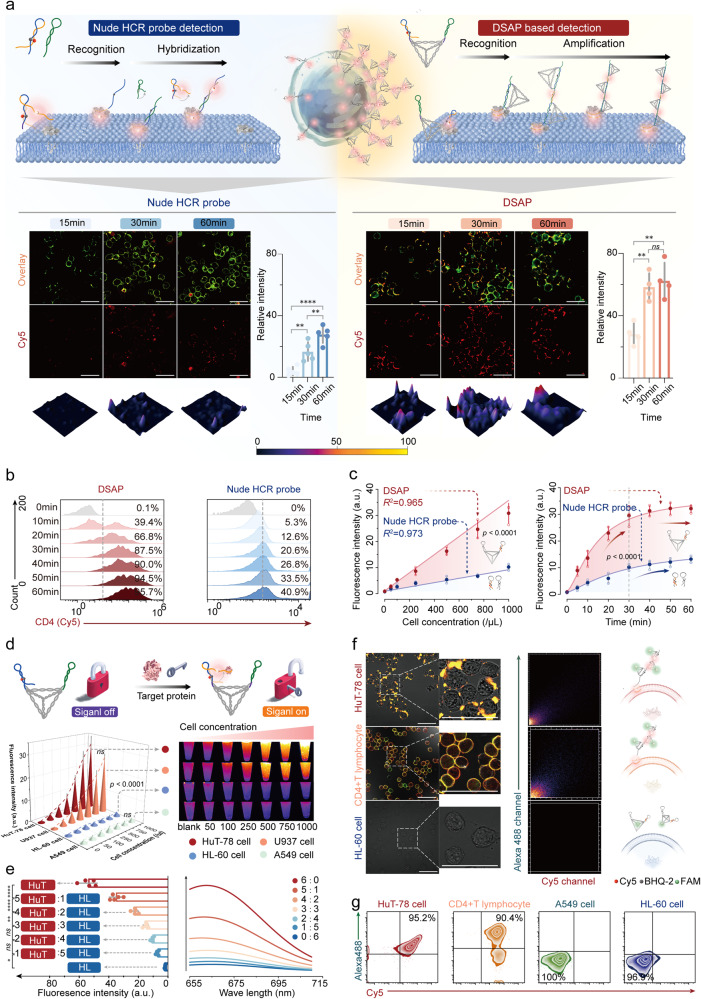
The excellent sensitivity, specificity, and stability of the DSAP. **a** The comparison between the DSAP and nude HCR probes in detecting membrane protein. CLSM images of HuT-78 cells treated with DSAP and nude HCR probes for 15, 30, and 60 min, respectively. The cell membranes were stained with DiO dye (green channel), and the released fluorescence from DSAP was Cy5. The statistical results of nude HCR probes and DSAP were shown by histogram. **b** The FCA of HuT-78 cells treated with DSAP and nude HCR probes from 0 to 60 min at 10 min intervals. **c** The MFI of HuT-78 cells at increasing concentrations after treatment with DSAP and nude HCR probes. The MFI of HuT-78 cells treated with the DSAP and nude HCR probes from 0 to 60 min. **d** The schematic illustration of signal switching off/on DSAP. The MFI and visualization of CD4+ cells (HuT-78 cells and U937 cells) and CD4− cells (HL-60 cells and A549 cells) of varying concentration after treatment with the DSAP. **e** The MFI and emission wave curve of the mixture containing HuT-78 cells and HL-60 cells of varying concentration ratios after treatment with DSAP. **f** CLSM images of HuT-78 cell, CD4+ T lymphocyte, and HL-60 cell, as well as colocalization profiles of Alexa 488 channel and Cy5 channel after treating FAM/Cy5/BHQ-2 labeled DSAP. The CLSM images were overlaid by the Cy5 channel and FAM channel. **g** The FCA of HuT-78 cell, CD4+ T cell, A549 cell and HL-60 cell treated with FAM/Cy5/BHQ-2 labeled DSAP. The error bars in (**a**, **c**–**e**) are determined by the SD of the MFI from at least three parallel experiments. All the tested samples were technical replicates. The **** represented *p* < 0.0001, ** represented *p* < 0.01. Scale bars, 50 μm

CD4 DSAP was used as a model system to validate the sensitivity, specificity, and structural stability of DSAP. To validate the sensitivity of DSAP, CD4-positive (CD4+) HuT-78 cells were chosen, and the CD4 phenotype was validated by FCA (Supplementary Fig. S23). HuT-78 cells were treated with the nude HCR probe and DSAP for 15, 30, and 60 min, respectively. According to CLSM, HuT-78 cells treated with DSAP exhibited three times higher fluorescence intensity on the membrane than those treated with nude HCR probes at 15, 30, and 60 min (*p* < 0.0001). The fluorescence intensity of the cells treated with DSAP remained almost unchanged from 30 to 60 min (*p* > 0.05), whereas a notable increase was observed in the cells treated with nude HCR probe from 0 to 60 min (Fig. 5a, *p* < 0.01). The Cy5 fluorescence released from the DSAP was overlaid by the DiO-labeled membrane surface. Minimal fluorescence can be observed in the cytoplasm, indicating minimal interference from the cytoplasm (Fig. 5a). Meanwhile, FCA revealed that Cy5+ ratios in HuT-78 cells treated with the DSAP reached saturation after 30 min incubation. Conversely, the Cy5+ ratios of HuT-78 cells treated with nude HCR probes increased slowly from 0 to 60 min. In the DSAP treatment group, the MFI continuously increased from 0 to 30 min; nevertheless, no significant difference was observed after 30 min (Fig. 5b and Supplementary Fig. S24). By contrast, the MFI of cells treated with the nude HCR probe continued to increase from 0 to 60 min, which was approximately three times lower than that in the DSAP group at 30 min (*p* < 0.0001; Fig. 5b and Supplementary Fig. S25). Besides, microwell fluorescence was also measured to explore the sensitivity. DSAP was treated with diluted HuT-78 cells of varying concentrations from 50 to 1000/µl for 30 min at 25 °C. The MFI of cells treated with DSAP was higher than that of cells treated with nude HCR probes. A strong linear correlation was observed between cell concentration and MFI detected by the nude HCR probe (*R*^2^ = 0.973) and DSAP (*R*^2^ = 0.965). As revealed by the LoD formula (LoD = 3σ/S), the LoD of the DSAP at 32/100 μl was 40 times lower than that of the nude HCR probes at 13,000/ml. The HuT-78 cells were treated with DSAP and nude HCR probes for 0 to 60 min. This revealed that the MFI of the DSAP treatment group reached saturation after 30 min. Conversely, the MFI of the nude HCR probe group continued to increase from 0 to 60 min (Fig. 5c). At 30 min, the MFI of the DSAP treatment group was approximately three times higher than that of the nude HCR probe treatment group (*p* < 0.0001), which was consistent with the CLSM and FCA results.

In addition to remarkable sensitivity, as a signal-switching off/on probe, DSAP is endowed with superior specificity compared with those constantly fluorescent probes.^40^ The specificity of DSAP was investigated by the CD4+ HuT-78 and U937 cells, CD4 negative (CD4−) HL-60, and A 549 cells (phenotype verified by FCA, Supplementary Figs. S23 and S26). When CD4+ and CD4− cells of various concentrations from 50 to 1000/μl were treated with DSAP, the increase in detection signals was observed only in the CD4+ cells, while rare fluorescent signals were detected in the CD4− cells according to microwell fluorescence measurement and fluorescence visualization (Fig. 5d). According to CLSM, rare Cy5 fluorescence was shown on the membrane of HL-60 (Supplementary Fig. S27). To explore whether DSAP can detect target cells from scramble cells in complex systems, we mixed HuT-78 and HL-60 cells at varying concentration ratios. Subsequently, we treated cell mixtures of different cell ratios with DSAP for 30 min at 25 °C. According to microwell fluorescence measurement, as the proportion of HuT-78 cells increased in cell mixtures, the MFI of cell mixtures increased. Meanwhile, the emission-wave curves of different cell mixtures showed that the highest emission peak occurred at ~650 nm (Fig. 5e).

Alexa 488 was conjugated to the 5’ end of the S4 chain of DTF-SE, Cy5, and BHQ-2, and was modified into the stem regions of H1-SE. Thus, Alexa 488/Cy5/BHQ-2 labeled CD4 DSAP was constructed to validate their structural stability during interactions with cell membrane proteins. The HuT-78 cells, isolated CD4+ T lymphocytes, and HL-60 cells were treated with Alexa 488/Cy5/BHQ-2 labeled CD4 DSAP for 30 min at 25 °C. The CLSM results revealed extensive fluorescence colocalization between Alexa 488 and Cy5 on the membrane surface of CD4+ cells. The fluorescence colocalization profiles of Cy5 and Alexa 488 were assessed, with Pearson’s correlation coefficient values equal to 0.951 for HuT-78 cells and 0.794 for CD4+ T lymphocytes, respectively. In contrast, HL-60 cells exhibited no colocalization on the membrane surface (Fig. 5f). Meanwhile, FCA demonstrated that ~90.4% of CD4+ T lymphocytes and 95.2% of HuT-78 cells were Alexa 488+/Cy5+. However, the HL-60 cells and A549 cells showed no FAM or Cy5 fluorescence (Fig. 5g and Supplementary Fig. S28).

### Validation of the DSAP for diverse immune-cell phenotypes

DSAP was proposed to address the contemporary challenges in flow-cytometric immune-cell phenotyping. We constructed Cy5/BHQ-2-labeled CD4 DSAP, FAM/BHQ-1-labeled CD8 DSAP, and FAM/BHQ-1-labeled CD14 DSAP to detect CD4+ T, CD8+ T lymphocytes, and monocytes. The detection performance of the DSAP was examined by assessing its sensitivity, specificity, multiplex labeling capacity, and correlation between concentrations and signals.

**Fig. 6:**
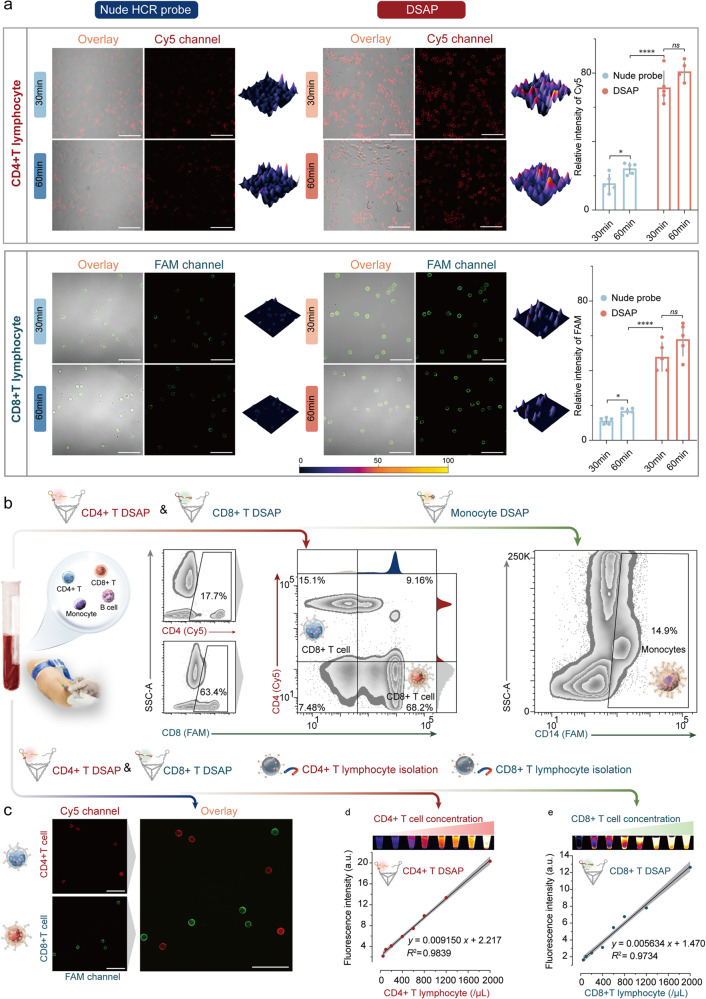
The validation of DSAP for diverse immune cells. **a** The CLSM results of isolated human CD4+ T and CD8+ T lymphocytes treated with CD4 nude HCR probes and DSAP as well as CD8 nude HCR probes and DSAP for 30 and 60 min, respectively. The CD4 DSAP and nude HCR probes were modified with Cy5/BHQ-2, and the CD8 DSAP and nude HCR probes were modified with FAM/BHQ-1. The histogram on the right shows the statistical results from three parallel experiments. **b** The FCA of whole blood cells treated with CD4 DSAP and CD4 DSAP and CD14. **c** The CLSM of blood samples treated with both CD4 DSAP (red channel) and CD4 DSAP (green channel). **d** The MFI of isolated human CD4+ T lymphocytes at increasing concentrations after treatment with the DSAP. **e** The MFI of isolated human CD8+ T lymphocytes of increasing concentrations after treatment with the CD4 DSAP and CD4 DSAP. The dots in (**d**) and (**e**) represent the mean average of MFI from at least three parallel experiments. The gray bands in (**d**) and (**e**) represent the 95% confidence interval band. All the tested samples were technical replicates. Scale bars, 50 μm

The DSAP exhibited significant signal amplification owing to the spatial confinement of DTF, as confirmed by CLSM. As indicated by the results, about 3 ~ 4 times stronger MFI was observed on the membrane surface of the isolated CD4+ T lymphocytes and CD8+ T lymphocytes in the DSAP group compared with those in the nude HCR probe group following treatment periods of 30 min and 60 min (*p* < 0.0001). No statistically significant MFI differences were observed between the 30- and 60-min treatment periods in the DSAP group, thus indicating signal saturation. In contrast, the nude HCR probe group yielded a substantial increase in MFI from 30 to 60 min (*p* < 0.05, CD4+ T lymphocytes; *p* < 0.01, CD8+ T lymphocytes; Fig. 6a).

To demonstrate its specificity, blood samples were incubated with CD4+ T lymphocyte and CD4 DSAP at 25 °C for 30 min after red blood cell lysis and washed twice before analysis. As indicated by FCA, Cy5+/FAM− and FAM+/Cy5− cell populations were observed in the lymphocyte population (Fig. 6b). Interestingly, two evident cell populations were identified in the FAM-positive cell group: FAM-low and FAM-high (Fig. 6b). A reasonable explanation for this phenomenon is that the aptamer primarily recognizes the CD8 alpha chain, of which variable expression levels in the CD8+ T lymphocyte population indicate different functional and differentiation statuses. Additionally, most monocytes yielded FAM signals after treatment with CD14. Notably, the three types of DSAP rarely exhibited nonspecific signals in the undesired cell populations (Fig. 6b and Supplementary Fig. S29). Furthermore, according to CLSM, Cy5, and FAM fluorescence signals emerged on the membrane surfaces of distinct cells after the cells were treated with CD4+ T and CD4 DSAP (Fig. 6c). The finding demonstrated the excellent specificity of DSAP.

To demonstrate the linear correlation between MFI and concentrations, CD4+ and CD8+ T lymphocytes were isolated from the blood and diluted to various concentrations (50/μl to 2000/μl). The chosen concentration range encompasses the majority of CD4+ and CD8+ T lymphocyte concentrations typically observed in clinical blood samples. After treatment with CD4+ and CD4 DSAP, a strong linear correlation was revealed between the MFI and cell concentrations, with *R*^2^ coefficients equal to 0.97 and 0.98 for CD4+ T and CD4 DSAP, respectively (Figs. 5f and 6e). The LoD for CD4+ T lymphocytes was 1/100 μl, while for CD8+ T lymphocytes, it was 4/100 μl. These values were sufficient to meet the clinical demands of cell phenotyping. The robust linear correlation indicates the potential of DSAP to quantify cell concentrations in blood samples.

### High-accuracy responses of DSAP in clinical applications

**Fig. 7:**
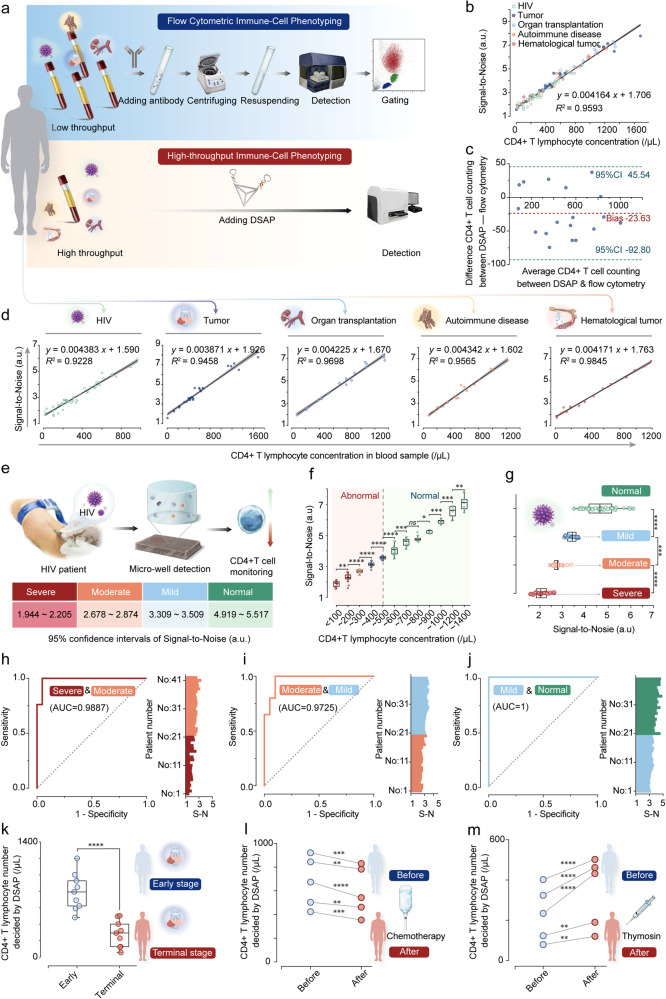
The high accuracy in CD4+ T detection and HIV immunodeficiency staging by DSAP. **a** The workflow of flow cytometric immune-cell phenotyping and DSAP-based high-throughput immune-cell phenotyping for blood samples analysis. The dots represent S-N according to at least three times parallel experiments. The gray bands represent 95% confidence interval bands. **b** The linear relationship between S-N decided by DSAP and the CD4+ T lymphocyte number decided by flow cytometry. **c** The Bland-Altman analysis of the CD4+ T lymphocyte number decided by flow cytometry and DSAP when detecting 18 blood samples. The upper and lower limit 95% confidence intervals and the mean bias of difference values are shown. **d** DSAP measured the blood samples from the patients with various diseases, and the linear relationships between S-N decided by DSAP and the CD4+ T lymphocyte number decided by FCA were displayed. The dots represent S-N according to at least three times parallel experiments. The gray bands represent 95% confidence interval bands. **e** The schematic illustration of CD4+ T lymphocyte monitoring by DSAP for HIV patients. The 95% confidence intervals of S-N were decided by DSAP when detecting blood samples from patients with mild, moderate, and severe immunodeficiency. **f** The boxplot represents the signal-to-noise by DSAP when detecting blood samples with different CD4+ T lymphocyte concentrations. **g** The signal-to-noise by DSAP when detecting blood samples from HIV patients with mild, moderate, and severe immunodeficiency. The ROC curve reflects the diagnostic accuracy of DSAP in diagnosing mild versus moderate immunodeficiency (**h**), moderate versus severe immunodeficiency (**i**), and severe immunodeficiency versus normal conditions (**j**). **k** The CD4+ T lymphocyte number was decided by DSAP of ten cancer patients of the terminal stage and ten of the early stage. The dots represent the CD4+ T lymphocyte mean concentration of every patient by DSAP, according to three repetitive experiments. **l** The CD4+ T lymphocyte number decided by DSAP for five patients before and after chemotherapy. The dots represent the CD4+ T lymphocyte mean concentration decided by DSAP, according to three repetitive experiments. **m** The CD4+ T lymphocyte number decided by DSAP for five patients before and after thymosin. The dots represent the CD4+ T lymphocyte mean concentration decided by DSAP, according to three repetitive experiments. All the tested samples were technical replicates. The **** represented *p* < 0.0001, *** represented *p* < 0.001, ** represented *p* < 0.01, * represented *p* < 0.05. The abbreviation S-N re*p*resents signal-to-noise

Because of its superior sensitivity, specificity, multiplex labeling, and concentration-signal correlation attributes, the DSAP has emerged as a promising technique for rapid immune monitoring. Compared with FCA, which employs microbeads as an internal reference for immune-cell quantification, the MFI released by DSAP maintained a robust linear correlation with the target cell concentration in blood samples. Cell concentrations can be calculated according to fitted linear equations, which reduces both the cost and technical sensitivity, enhancing the efficiency and precision of clinical immunotyping (Fig. 7a). As a preliminary test, the mean fluorescence intensities of CD4+ T lymphocytes in blood samples from healthy individuals (10 samples, Supplementary Fig. S30) and patients with different diseases/states were determined, including HIV (10 samples, Supplementary Fig. S31), tumors (10 samples, Supplementary Fig. S32), organ transplantation (10 samples, Supplementary Fig. S33), hematological tumors (10 samples, Supplementary Fig. S34), and autoimmune (10 samples, Supplementary Fig. S35). The mean fluorescence intensity of CD4+ T lymphocytes yielded no differences between healthy individuals and patients and no difference among various diseases (Supplementary Fig. S36). Similar expression levels of CD4 on CD4+ T lymphocytes from different immune statuses were observed, consistent with previous reports.^2,41,42^ Thus, the microwell MFI can indicate concentration variations in CD4+ T lymphocytes.

We selected 120 random blood samples from patients who required regular monitoring of their immune statuses, including those diagnosed with HIV (45 samples), hematological tumors (17 samples), tumors (25 samples), autoimmune diseases (18 samples), and those who had undergone organ transplantation (17). We observed that the signal-to-noise ratios (S-N, determined by the MFI of the sample to blank microwells) of the DSAP exhibited a robust linear correlation with the FCA results of the 120 blood samples, with an *R*^2^ coefficient of 0.959. Furthermore, a robust linear correlation between the S-N of the DSAP and clinical results from patients suffering from a wide variety of diseases was identified, with *R*^2^ values >0.92 (Fig. 7d and Supplementary Table 2).

Expanding on the detection results of CD4+ T lymphocytes in the 120 blood samples, the linear equation of S-N and clinical results (*y* = 0.004164 *x* + 1.706) was fitted, where *y* symbolizes the signal-to-noise ratios from three parallel measurements, and *x* represents the CD4+ T lymphocyte concentrations in blood samples (cells/μl). This linear equation allowed us to quantify the CD4+ T lymphocyte concentration in blood samples based on the S-N. We further explored the stability of detection results based on DSAP during the detection of CD4+ T lymphocytes. The immuno-trol cells with low and high concentrations of CD4+ T lymphocytes were treated with DSAP every day for 7 days. The concentration decided by DSAP showed no statistical difference among these results from everyday detection. Therefore, the detection results of DSAP remain relatively stable (Supplementary Fig. S37). We then compared detection result consistency between DSAP and FCA. The concentrations of CD4+ T lymphocytes in the 18 blood samples were concurrently detected using DSAP and FCA. The Bland–Altman plot demonstrates that all the difference values of the 18 samples were within the 95% agreement limits, and the mean bias between the two methodologies was −23.63, which lies within the clinically acceptable error range. Accordingly, these two methods are comparable (Fig. 7c and Supplementary Table 3).

As a proof-of-concept, this methodology could serve as a valuable tool for immune status monitoring, particularly in patients with HIV. The concentration of CD4+ T lymphocytes typically lies between 500/µl to 1600/μl in healthy individuals. As HIV attacks CD4+ T lymphocytes, a progressive decrease in the CD4+ T lymphocyte population signifies immune function degradation.^43^ CD4+ T lymphocyte concentrations lower than 500/µl signals the onset of immunodeficiency, concentrations between 350/µl and 500/µl indicate mild immunodeficiency, concentrations between 200/µl and 350/µl signify moderate immunodeficiency, and a level below 200 points demarcates severe immunodeficiency.^44^

Our study examined the 95% confidence intervals (CIs) of the DSAP signal-to-noise ratios among HIV patients. The 95% CIs of the signal-to-noise ratio for DSAP results from patients in the mild, moderate, severe immunodeficiency, and normal stages were 3.309–3.509, 2.678–2.874, 1.944–2.205, and 4.919–5.517, respectively (Fig. 7e). The boxplot in Fig. 7f illustrates the signal-to-noise for various CD4+ T lymphocyte concentration intervals. As shown, statistically significant differences exist between interval concentrations (*p* < 0.05) (except the 600–700/µl and 700–800/µl intervals) (*p* = 0.7250, Supplementary Table 4). We also examined the signal-to-noise distribution of HIV patients in the mild, moderate, and severe immunodeficiency stages; statistically significant differences were exhibited among the DSAP results at each stage (*p* < 0.001) (Fig. 7g and Supplementary Table 5). We applied receiver-operating characteristic curve to evaluate the accuracy of DSAP in diagnosing mild, moderate, and severe immunodeficiency. The analysis demonstrated the robust diagnostic performance of DSAP in classifying normal vs. mild (area under curve (AUC) = 1, Supplementary Table 6), mild vs. moderate (AUC = 0.9725, Supplementary Table 7), and moderate vs. severe immunodeficiency stages (AUC = 0.9887, Supplementary Table 8, Fig. 7f–i and Supplementary Fig. S38), thus substantiating the effectiveness of our methodology for the rapid assessment of immunodeficiency stages in HIV patients.

Furthermore, we applied DSAP to perform immune monitoring for cancer patients at different stages. For patients at an early stage, their immune system remains healthy. The CD4+ T lymphocyte number is usually normal if the patient does not suffer from other diseases. By contrast, the immune system faces possible collapse due to the tumor invasion.^45^ The CD4+ T lymphocyte number usually decreases significantly. Therefore, immune monitoring for cancer patients can provide important indications for cancer treatment planning and prognosis.^46^ First, we collected blood samples from 20 patients with malignant solid tumors, including 10 patients at the early stage and 10 patients at the terminal stage, diagnosed based on clinical symptoms and radiological examination. Subsequently, we detected CD4+ T lymphocytes in the 20 tubes of blood samples by our proposed DSAP. According to the analysis, significant differences in CD4+ T lymphocyte numbers between early- and terminal-stage patients can be observed (*p* < 0.0001). Therefore, DSAP rapidly evaluates the immune status of the patients in the cancer process (Fig. 7k). Second, we applied DSAP to carry out immune monitoring for cancer patients undergoing chemotherapy. The chemotherapy is proven to inhibit CD4+ T lymphocyte proliferation, leading to a significant decrease in the peripheral CD4+ T lymphocyte number.^47^ Therefore, immune monitoring is essential for controlling chemotherapy drug doses. First, we follow up with five patients undergoing chemotherapy. We detected the CD4+ T lymphocyte number in the blood samples from the five patients before and after chemotherapy using DSAP. According to the results, we observed that the CD4+ T lymphocyte number decreased significantly after chemotherapy for the five patients (*p* < 0.0001). Therefore, DSAP can rapidly provide information for clinicians regarding the immune status to adjust treatment planning (Fig. 7l). Third, we applied DSAP to conduct immune monitoring for patients undergoing thymosin treatment. As an immune effector, thymosin can stimulate T lymphocyte proliferation and differentiation, and thymosin is usually used to modulate immune function for cancer patients.^48^ Following the administration of thymosin, the peripheral CD4+ T lymphocyte will increase to a certain extent. Therefore, immune monitoring is used to evaluate the treatment effect of thymosin. We followed up with five cancer patients undergoing thymosin. We detected the CD4+ T lymphocyte number of the blood samples before and after thymosin injection using DSAP. According to the results, we can confirm that thymosin can improve CD4+ T lymphocytes significantly (*p* < 0.0001). Therefore, DSAP can be used to evaluate the treatment effect of thymosin for cancer patients (Fig. 7m).

## Discussion

Aimed at the bottleneck of rapid immune-cell phenotyping, we introduced DSAP-based DSAP through microwell quantitative analysis to achieve 30-min rapid immune monitoring, which provides opportunities for mass health screenings and point-of-care device development. According to previous studies on constructed aptasensors for cell phenotyping, the complex nature of the blood environment causes deficient sensitivity and specificity, and tedious cell isolation or protein extraction manipulation harms accuracy.^49,50^ Additionally, the prolonged detection time of aptasensors could cause increased false-positive signals due to the phagocytosis effect of monocytes and neutrophils in the blood.^51^ To overcome the challenges, we proposed DSAP by structure-guided post-SELEX optimization and DTF-structured HCR probe design, which enhanced target recognition and signal amplification. As a proof-of-concept, DSAP contained three kinds of representative immune cells: CD4+ T, CD8+ T lymphocytes, and monocytes. The DSAP for other immune markers can also be developed based on identical methods, and the general applicability of DSAP in multiplex immune phenotyping was confirmed using DSAP with different labeled fluorophores. Current researches have designed biosensors for detecting single immune cells,^52^ whereas limited applicability could not achieve immune-cell phenotyping (e.g., innate and adaptive immune systems). The development of high-throughput flow cytometry has been a research hotspot. However, it has needed to be optimized in clinical applications because of high costs, intricate data processes, and limited device functions.^13,53^ Furthermore, microfluidic flow cytometry was proposed due to automatic sample analysis but is obstructed by sample preprocessing and equipment requirements.^54^ Unlike the above-mentioned methods, DSAP can quantitatively analyze diverse immune cell subsets through microwell fluorescence measurement, lowering cost, technology requirement, and interpretation complexity. DSAP is a promising tool for high-throughput immune monitoring, especially in mass health screenings and resource-limited areas, as well as boosting the development of point-of-care devices for immune monitoring.

The screening and optimizing high-quality aptamer are challenging, which determine reaction kinetics and detection specificity. Besides structure-guided post-SELEX to functionalize the DSAP, further improvement is needed to establish more high-affinity aptamers for other immune markers. The versatility and efficiency of DSAP could be further extended if recent breakthroughs in aptamer optimizations were encompassed. Kelly et al.^55^ proposed a Pro-SELEX chip with varying linear velocity to isolate the high-affinity aptamer based on varying magnetic levels of aptamer-modified particles, related to the amount of bound target. Stojanovic et al.^56^ established a functional group–guided aptamer optimization, overcoming the steric hindrance between aptamer and target to boost the high-affinity of aptamers. Their novel strategies can benefit DSAP construction and establish versatile and comprehensive DSAP for immune monitoring in broader scenarios. Furthermore, the remaining vertexes or skeletons of DTF can also be used to load HCR probes to construct the multi-target biosensors. Even the AND logic gate-based DTF-structured biosensors can also be designed to enhance the detection specificity. The DTF-structured probe can achieve intracellular analyte detection due to its excellent endocytosis capacity. And the lysosomal escape capacity of DTF has been demonstrated in previous studies via the caveolin-mediated endocytosis pathway.^57^ Therefore, some scholars fabricated DTF-based probes to detect intracellular miRNA or mRNA with high specificity.^58^ The other framework nuclear acids, such as DNA triangular prism and DNA cube, can also be used as a carrier to construct the framework nuclear acid-structured HCR probes and increase HCR efficiency via spatial confinement and orientation control. Bui et al.^59^ designed a localized HCR device on the DNA origami. The distances and positions of HCR probes can be controlled. The localized HCR device can significantly improve the detection speed than nude HCR probes. Besides improving detection speed, the framework nucleic acid can also improve the detection specificity via the spatial confinement effect. Fu et al.^60^ utilized the framework nucleic acid to construct a molecular sieve to discriminate between mature miRNA and pre-miRNA via a size-selective way. The detection specificity can be improved in detecting miRNA.

In summary, DSAP for rapid 30-min immune monitoring was proposed, based on high performance, which was functioned by structure-guided post-SELEX optimization and DTF-structured HCR probes. DSAP can also become the assembly platform of diagnosis and therapy for immunologic dysfunction. First, the self-assembling and amplified DNA duplex formed by HCR can provide many binding sites for molecular dug via the groove binding or covalent modification.^61^ Wang et al.^22^ conjugated a synthesized prodrug into the terminal of H2. The activation of HCR can cause the accumulation of these prodrugs on the surface of targeted cells. The HCR polymers can be internalized into the targeted cells via an endocytosis pathway. The stronger cell cytotoxicity can be observed by HCR probe-functioned prodrugs than traditional prodrugs. Second, DTF is an excellent drug delivery platform for nuclear acid and peptide drugs due to excellent endocytosis, programmability, and biocompatibility.^57,62–64^ The miRNA or peptide can be loaded into the vertexes or skeletons of DTF. The DTF can significantly improve the serum resistance and cellular uptake of the loaded drugs. Besides, the excellent tissue permeability of the DTF-structured probes allows its precise living imaging of solid tumors.^65^ Third, DSAP can recognize immune cells specifically by aptamer, which reduces the side effects of immune therapy and improves the efficacy of anti-tumor therapy. The tumor-related protein degradation is a heated concern in anti-tumor therapy, such as HER2 and PDL1. The DSAP can enhance the targeted protein into lysosomal pathway-mediated degradation, exerting the tumor suppression effects.^66^ Last, the DTF-based HCR probes can be used in cell membrane surface engineering via mediating cell-to-cell interaction, cellular protection, and cellular capture. The activation of HCR can accumulate the linkers between cellular contact zones to enhance the interaction stability.^67^ Besides, the HCR on the cellular surface can form biomimetic cell walls with the help of Ca^2+^ to protect the cell in complex environments.^68^ Meanwhile, cellular capture can be achieved by the DTF-structured HCR strategy due to the formation of hydrogel-based cell surfaces. Therefore, DSAP has enormous potential to facilitate the diagnosis and therapy of immunologic dysfunction. Meanwhile, framework nucleic acid-structured HCR probes can be a promising strategy for solving the challenges of immune monitoring and biotherapy.

## Materials and methods

### Construction and characterization of the DSAP

Four oligonucleotide chains, S1-SE, S2-SE, S3, and S4, were quantified to 100 uM by Nanodrop One (Thermo Fisher Scientific). H1-SE and H2-SE were quantified to 10 uM. The four oligonucleotide chains were mixed in equal molar weight with TM buffer, resulting in their final concentration of 2 uM. The DTF-SE, H1-SE, and H2-SE were synthesized by denaturation at 95 °C for 10 min, followed by 4 °C annealing for 20 min. DTF-SE and H1-SE were mixed at a molar concentration ratio 1:1 at 25 °C for 10 min to obtain H1-DTF. DTF-SE, H1-SE, and H2-SE were mixed at a molar concentration ratio 1:1:1 at 25 °C for 10 min to obtain DSAP. The synthesized DSAP was purified through HPLC.

One μl S1-SE was mixed with 1 μl S2-SE. Next, the mixture was heated to 95 °C and annealed to 4 °C by thermocycler (SimpliAmp, Thermo Fisher Scientific) to synthesize S1-SE + S2-SE. The S1-SE + S2-SE + S3 is synthesized according to the same procedures. To characterize the successful step-wise synthesis of DSAP, 1 uM S1, S1 + S2, S1 + S2 + S3, DTF-SE, H1-SE, H2-SE, H1-DTF, and DSAP were subjected to 2% agarose gel electrophoresis (AGE) at 120 V for 30 min in 0.5×TBE buffer and capillary electrophoresis (Qsep100 Bioptic). The AGE results were visualized by scanning the gels with the iBright gel imaging system (Thermo Fisher Scientific).

For AFM measurement, a 9.9 nm mica (Bruker) was positively modified using a 100 mM NiCl_2_ solution for 5 min and then dried with nitrogen. A mixture of 25 µl 20 nM DSAP and 25 µl fixation solution (10 mM MgCl_2_, 25 mM KCl, 10 mM HEPES, pH = 7.5) was dropped onto the mica for 10 min. The mica was gently washed, and 50 ul of imaging solution (10 mM NiCl_2_, 25 mM KCl, 10 mM HEPES, pH = 7.5) was then dropped onto the mica, and the sample observed using an AFM (Cypher VRS, Oxford Instruments, United Kingdom). The particle size of DTF-SE and DSAP as well as the potential of DTF-SE, H1-DTF, and DSAP were measured using the Zetasizer Nano ZS90 (Malvern Instrument Ltd., Malvern, United Kingdom). The 20 repetitive measurements were conducted for every sample.

### The validation of aptamer and DSAP detection capacity based on flow cytometry

To explore the affinity changes of aptamers in the truncation process, a total of 1 × 10^5^ isolated human CD4+ T lymphocytes, CD8+ T lymphocytes, and PBMCs were treated with 200 nM of their corresponding aptamers in the binding buffer at 25 °C for 30 min. The CD4+ T lymphocytes were treated with 200 nM original aptamer, CD4 T1, CD4 T2, and truncated aptamer for 30 min. The CD8+ T lymphocytes were treated with original aptamer, CD8 T1, CD8 T2, and truncated aptamer. The PBMCs were treated with original aptamer and truncated aptamer. The cells were washed twice with binding buffer and resuspended with 200 µl binding buffer for subsequent FCA (AttuneNxT, Thermo Fisher Scientific). To calculate the *K*_D_, isolated human CD4+ T lymphocytes, CD8+ T lymphocytes, and PBMCs cells were incubated with original and truncated aptamer of various concentrations (ranging from 5 nM to 200 nM) at 25 °C for 30 min. The MFI of 1 × 10^4^ recorded target cells in each concentration sample was measured by three parallel experiments. To evaluate the detection capacity of the aptamers in whole blood samples, the blood samples after red cell lysis were treated with 200 nM Cy5-labeled CD4 truncated aptamer, 5 µl PE-labeled CD19 antibody, FAM-labeled CD8 truncated aptamer, and FAM-labeled CD14 aptamer for 30 min at 25 °C. After washing with binding buffer twice, the cell suspension was analyzed by flow cytometry.

To compare the detection efficiency of DSAP and nude HCR probes, 2 × 10^5^ HuT-78 cells were treated with 200 nM DSAP and nude HCR probes at 25 °C for 0, 10, 20, 30, 40, 50, 60 min. The fluorescence signal of HuT-78 cells was detected via flow cytometry in the Cy5 channel. The three parallel experiments were carried out for every time point. To validate the structural stability of DSAP, the FAM was modified onto the 5‘ end of the S4 strand of DTF-SE, and the FAM/Cy5/BHQ-2 DSAP was synthesized. The HuT-78 cells, CD4+ T lymphocytes, HL-60 cells, and A549 cells were treated with 200 nM FAM/Cy5/BHQ-2 DSAP at 25 °C for 30 min. The cell suspension was detected by flow cytometry. To validate the detection capacity of DSAP in whole blood samples, the blood samples after red blood cell lysis were treated with 200 nM CD4 DSAP, CD4 DSAP, and CD14 for 30 min at 25 °C in the dark. Afterward, the cell suspension was detected via flow cytometry.

### Micro-well fluorescence intensity measurement

To validate the influence of environment temperature on the HCR efficiency of DSAP with 13-nt sticky end, 10 µl of 1.2 µM Cy5/BHQ-2 DSAP were added to 40 µl diluted initiators (containing 10 mM Mg^2+^) to achieve the different target-to-DSAP molar ratios of 1:20, 1:10, 1:5, 1:2, and 1:1, respectively. The mixtures were incubated at 4, 25, and 37 °C for 30 min in the dark. The microplate reader measured the fluorescence intensity (Varioskan Lux, Thermo Fisher Scientific). (Cy5: excitation wavelength of 640 nm, emission wavelength of 670 nm, excitation bandwidth of 12 nm). The three parallel experiments were carried out. To explore the influence of sticky end length on the HCR efficiency of DSAP, a total of 10 µl of 1.2 µM DSAP with sticky end lengths, including 13, 17, and 21 nt were added to 40 µl diluted initiators (containing 10 mM Mg^2+^) to achieve the different target-to-DSAP ratios of 1:20, 1:10, 1:5, 1:2, and 1:1, respectively at 25 °C in the dark. The parameters mentioned above were used for the micro-well fluorescence detection. The three repetitive experiments were carried out. When validating the influence of Mg^2+^ concentration on the HCR, a total of 10 µl of 1.2 µM DSAP with 13 nt sticky ends were added to 50 µl diluted initiators (containing 2, 10, and 50 mM Mg^2+^) to achieve the different target-to-DSAP ratios of 1:20, 1:10, 1:5, 1:2, and 1:1, respectively. Following a 30-min incubation at 25 °C in the dark, the fluorescence intensity of Cy5 was measured by the microplate reader for every micro-well.

To explore the influence of the mismatch in the breathing sites of H2-SE on the metastability of HCR probes, we designed the H2-SE without mismatch (H2-SE NM), H2-SE with one mismatch in one breathing site (H2-SE OM), and H2-SE with two mismatches in two breathing site (H2-SE). The 25 µl Cy5/BHQ-2 labeled H1-SE were treated with 25 µl H2-SE NM, H2-SE OM, and H2-SE for 30 min at 25 °C. The three parallel experiments were conducted, and the Cy5 fluorescence intensity was measured by the microplate reader. Ten µl samples from the three groups (H1-SE/H2-SE NM, H1-SE/H2-SE OM, and H1-SE/H2-SE) were mixed with 6×loading buffer (Takara). The mixtures were loaded into the wells of agarose gel, and electrophoresis was conducted. The Cy5 and GelRed signal was detected via the iBright gel image system.

To compare the HCR efficiency of DSAP and nuder HCR probes, HuT-78 cells were serially diluted into gradients of 1000/µl, 750/µl, 500/µl, 250/µl, 100/µl, and 50/µl with the binding buffer. Subsequently, 10 µl of 1.2 µM Cy5/BHQ-2 DSAP was added to 50 µl of diluted cell suspension, 2 µl of 5.2 µM Cy5/BHQ-2 H1-SE and H2-SE were added to the 50 µl diluted cell suspension. Following a 30-min incubation in the dark, Cy5 fluorescence intensity was measured using the microplate reader. The three parallel experiments were conducted. For characterization of reaction kinetics, 10 µl of 1.2 µM Cy5/BHQ-2 DSAP was added into 50 µl of HuT-78 cells of the concentration of 1000/µl. Two µl of 5.2 µM Cy5/BHQ-2 H1-SE and H2-SE were added to 50 µl of HuT-78 cells of the concentration of 1000/µl. The mixtures were incubated at 25 °C in the dark for 0, 10, 20, 30, 40, 50, and 60 min. The fluorescence intensity of Cy5 was measured using the microplate readers. The three parallel experiments were conducted. For validating specificity, 10 µl of 1.2 μM DSAP were added to 50 µl of HuT-78, U937, HL-60, and A549 cells, which were diluted to concentrations of 1000/µl, 750/µl, 500/µl, 250/µl, 100/µl, 50/µl respectively for 30 min at 25 °C. The Cy5 fluorescence intensity is measured by the microplate reader. In addition, to validate the capability of distinguishing target cells from scrambled cells, HuT-78 and HL-60 cells were mixed at varying number ratios of 0:6, 1:5, 1:2, 1:1, 2:1, 4:1, and 6:0. The mixtures were treated with the 200 nM DSAP for 30 min at 25 °C, and the microplate reader measured fluorescence intensity.

To explore the performance of DSAP in detecting CD4+ T and CD8+ T lymphocytes. The CD4+ T and CD8+ T lymphocytes were isolated from the whole blood samples and were diluted into 2000/ul, 1200/ul, 800/ul, 600/ul, 400/ul, 200/ul, 100/ul using the binding buffer. A 10 µl of 1.2 µM CD4 DSAP and CD8 DSAP was added to 50 µl of diluted CD4+ T and CD8+ T lymphocyte suspensions. The mixtures were incubated at 25 °C in the dark for 30 min. The detection signal of CD4 DSAP and CD8 DSAP was measured using the microplate reader. (FAM: excitation wavelength of 495 nm, emission wavelength of 512 nm, excitation bandwidth of 12 nm). All the micro-well fluorescence intensity measurements were conducted in at least three parallel experiments to ensure accuracy and reproducibility.

### Clinical blood sample testing by DSAP

All human blood samples in this experiment were obtained from the Department of Laboratory Medicine at West China Hospital following standard ethical protocols. To conduct the relationship analysis of DSAP and FCA in CD4+ T lymphocyte concentration evaluation, a total of 120 blood samples were randomly selected: 45 from HIV patients, 23 from tumor patients, 17 from organ transplant patients, 18 from autoimmune disease patients, and 17 from hematological tumor patients. CD4+ T lymphocyte detection was performed simultaneously by FCA and DSAP for these 200 blood samples. Before conducting FCA, the quality control of flow cytometry, including optical alignment, parameter compensation, and electronic standardization was carried out under clinical testing standards. In the pretreatment stage, blood samples in heparinized tubes both need to undergo the process of red blood cell lysis. In total, 100 µl whole blood samples were treated with 500 µl red blood cell lysis solution at 25 °C for 5 min. In the sample detection period, for FCA, 100 uL cell suspension was treated with 5 uL APC-labeled CD4 antibody at 25 °C in the dark for 30 min. For DSAP, 100 µl of cell suspension was added into one microwell and was treated with 20 µl of 1.2 µM DSAP at 25 °C in the dark for 30 min. Each sample testing by DSAP was repeated in at least three parallel experiments. In the signal reading period, the APC+ single cells flowing past excitation sources were detected for FCA, and the CD4+ T lymphocyte concentration could be calculated. For DSAP, the microplate reader can detect the fluorescence signal in microwells. Next, the S-N can be calculated according to the ratios of MFI between the sample and the blank group from three parallel experiments. The linear regression analysis can be conducted between S-N from DSAP and results from FCA, and the linear equation can be obtained. The CD4+ T lymphocyte concentration can be calculated using a linear equation in subsequent experiments. In the signal reading period, the time for DSAP is significantly shorter than FCA. The waiting time for FCA is about 6 h for the 120-tube samples, but for DSAP, the waiting time needed is only 1 min for 120-tube samples.

To confirm the consistency between DSAP and FCA, 18 blood samples were randomly chosen. Subsequently, CD4+ T lymphocyte was detected using FCA and DSAP simultaneously. For FCA, 100 µl of samples were treated with 5 ul APC-labeled CD4 antibody. For DSAP, 100 µl of samples were treated with 20 µl of 1.2 µM DSAP. After 30-min incubation at 25 °C in the dark, the concentration of CD4+ T lymphocytes was obtained by FCA. For DSAP, the S-N could be calculated from three parallel experiments, and the concentration of CD4+ T lymphocytes could be decided according to the linear equation mentioned above. Bland-Altman analysis was conducted to confirm the consistency.

For conducting immunodeficiency staging for HIV patients, 107 blood samples were obtained from HIV patients with mild (*n* = 25), moderate (*n* = 20), and severe immunodeficiency (*n* = 20), as well as normal immune function (*n* = 42). All the blood samples detected by DSAP were repeated in at least three parallel experiments, and S-N can be calculated for every sample. The ROC curve is presented according to the S-N values of the samples from the patients with normal/mild immunodeficiency, mild/moderate immunodeficiency, and moderate/severe immunodeficiency by DSAP. The AUC could be further calculated. The detection time for the 107 samples was 30 min at maximum.

To confirm the capacity to evaluate the immune status of the patients in the cancer process, we collected blood samples from 20 patients with malignant solid tumors, including 10 patients at the early stage and 10 patients at the terminal stage, which were diagnosed based on clinical symptoms and radiological examination. Next, we detected the CD4+ T lymphocyte of the 20 samples by DSAP, and the concentration of CD4+ T lymphocyte can be calculated by S-N values according to the above-mentioned linear equation. To validate the capacity to evaluate the immune status of the patients before and after chemotherapy, we followed up five patients undergoing chemotherapy and detected the CD4+ T lymphocytes of the five patients before and after chemotherapy by DSAP. The concentration of CD4+ T lymphocytes can be decided according to the method mentioned earlier. To evaluate the performance in immune monitoring of the patient undergoing immune treatment, We followed up with five cancer patients undergoing thymosin. We detected the CD4+ T lymphocytes before and after thymosin injection by DSAP. The concentration of CD4+ T lymphocytes can be decided according to the methods mentioned above.

### Supplementary information

Revised supporting information

## Data Availability

All data are available in the main text and the Supplementary Materials.

## References

[1] Hartmann FJ, Bendall SC (2020). Immune monitoring using mass cytometry and related high-dimensional imaging approaches. Nat. Rev. Rheumatol..

[2] Hiam-Galvez KJ, Allen BM, Spitzer MH (2021). Systemic immunity in cancer. Nat. Rev. Cancer.

[3] Ishiguro T (2017). An anti-glypican 3/CD3 bispecific T cell-redirecting antibody for treatment of solid tumors. Sci. Transl. Med..

[4] Cheng C (2020). Immune monitoring reveals fusion peptide priming to imprint cross-clade HIV-neutralizing responses with a characteristic early B cell signature. Cell Rep..

[5] Aarntzen EH, Figdor CG, Adema GJ, Punt CJ, de Vries IJ (2008). Dendritic cell vaccination and immune monitoring. Cancer Immunol. Immunother..

[6] Fernandez DM, Giannarelli C (2022). Immune cell profiling in atherosclerosis: role in research and precision medicine. Nat. Rev. Cardiol..

[7] Wunsch K (2022). COVID-19 in elderly, immunocompromised or diabetic patients-from immune monitoring to clinical management in the hospital. Viruses.

[8] Ghosh S (2023). Radiation-induced circulating myeloid-derived suppressor cells induce systemic lymphopenia after chemoradiotherapy in patients with glioblastoma. Sci. Transl. Med..

[9] Wang C, Liu J, Li W (2023). ‘Off the shelf’ immunotherapies: generation and application of pluripotent stem cell-derived immune cells. Cell Prolif..

[10] Fujita Y (2022). A versatile and robust cell purification system with an RNA-only circuit composed of microRNA-responsive ON and OFF switches. Sci. Adv..

[11] Cheng X (2007). A microfluidic device for practical label-free CD4(+) T cell counting of HIV-infected subjects. Lab Chip.

[12] Phillips D (2021). Highly multiplexed phenotyping of immunoregulatory proteins in the tumor microenvironment by CODEX tissue imaging. Front. Immunol..

[13] Lei C (2018). High-throughput imaging flow cytometry by optofluidic time-stretch microscopy. Nat. Protoc..

[14] Ding M, Kaspersson K, Murray D, Bardelle C (2017). High-throughput flow cytometry for drug discovery: principles, applications, and case studies. Drug Discov. Today.

[15] Hiramatsu K (2019). High-throughput label-free molecular fingerprinting flow cytometry. Sci. Adv..

[16] Figg CA, Winegar PH, Hayes OG, Mirkin CA (2020). Controlling the DNA hybridization chain reaction. J. Am. Chem. Soc..

[17] Bi S, Yue S, Zhang S (2017). Hybridization chain reaction: a versatile molecular tool for biosensing, bioimaging, and biomedicine. Chem. Soc. Rev..

[18] Yuan B (2020). Highly sensitive and specific detection of tumor cells based on a split aptamer-triggered dual hybridization chain reaction. Analyst.

[19] Wang X, Jiang A, Hou T, Li H, Li F (2015). Enzyme-free and label-free fluorescence aptasensing strategy for highly sensitive detection of protein based on target-triggered hybridization chain reaction amplification. Biosens. Bioelectron..

[20] Rafiee SD, Kocabey S, Mayer M, List J, Ruegg C (2020). Detection of HER2(+) breast cancer cells using bioinspired DNA-based signal amplification. ChemMedChem.

[21] Wu J, Lv J, Zheng X, Wu ZS (2021). Hybridization chain reaction and its applications in biosensing. Talanta.

[22] Wang YM, Wu Z, Liu SJ, Chu X (2015). Structure-switching aptamer triggering hybridization chain reaction on the cell surface for activatable theranostics. Anal. Chem..

[23] Chen J, Yang C, Nie H, Li H (2023). Aptamer recognition-promoted hybridization chain reaction for amplified label-free and enzyme-free fluorescence analysis of pesticide. Spectrochim. Acta A Mol. Biomol. Spectrosc..

[24] Zhu Y (2022). Double signal amplification strategy for dual-analyte fluorescent aptasensors for visualizing cancer biomarker proteins. Anal. Chem..

[25] Lackey HH, Peterson EM, Harris JM, Heemstra JM (2020). Probing the mechanism of structure-switching aptamer assembly by super-resolution localization of individual DNA molecules. Anal. Chem..

[26] Xie S (2023). Aptamer-based targeted delivery of functional nucleic acids. J. Am. Chem. Soc..

[27] Beyrampour-Basmenj H (2022). Sensitive and convenient detection of miRNA-145 using a gold nanoparticle-HCR coupled system: computational and in vitro validations. IEEE Trans. Nanobiosci..

[28] Li H (2021). Intracellular CircRNA imaging and signal amplification strategy based on the graphene oxide-DNA system. Anal. Chim. Acta.

[29] Tian T (2023). A dynamic DNA tetrahedron framework for active targeting. Nat. Protoc..

[30] Lin S (2022). Tetrahedral framework nucleic acids-based delivery promotes intracellular transfer of healing peptides and accelerates diabetic would healing. Cell Prolif..

[31] Zhang P (2020). Capturing transient antibody conformations with DNA origami epitopes. Nat. Commun..

[32] Gao S, Zheng X, Jiao B, Wang L (2016). Post-SELEX optimization of aptamers. Anal. Bioanal. Chem..

[33] Le TT, Chumphukam O, Cass AEG (2014). Determination of minimal sequence for binding of an aptamer. A comparison of truncation and hybridization inhibition methods. RSC Adv..

[34] Campos-Fernandez E, Barcelos LS, Souza AG, Goulart LR, Alonso-Goulart V (2020). Post-SELEX optimization and characterization of a prostate cancer cell-specific aptamer for diagnosis. ACS Omega.

[35] Sylvestre M (2020). Identification of a DNA aptamer that binds to human monocytes and macrophages. Bioconjug. Chem..

[36] Kacherovsky N (2019). Traceless aptamer-mediated isolation of CD8(+) T cells for chimeric antigen receptor T-cell therapy. Nat. Biomed. Eng..

[37] Fellows T (2020). Gold nanoparticle-streptavidin conjugates for rapid and efficient screening of aptamer function in lateral flow sensors using novel CD4-binding aptamers identified through Crossover-SELEX. Analyst.

[38] Dirks RM, Pierce NA (2004). Triggered amplification by hybridization chain reaction. Proc. Natl Acad. Sci. USA.

[39] Jia F (2021). Interaction between the functionalized probes: the depressed efficiency of dual-amplification strategy on ratiometric electrochemical aptasensor for aflatoxin B1. Biosens. Bioelectron..

[40] Hu M (2020). Signal-switchable lab-on-paper photoelectrochemical aptasensing system integrated triple-helix molecular switch with charge separation and recombination regime of type-II CdTe@CdSe core-shell quantum dots. Biosens. Bioelectron..

[41] Cachot A (2021). Tumor-specific cytolytic CD4 T cells mediate immunity against human cancer. Sci. Adv..

[42] Liu K (2021). A novel mouse model for liver metastasis of prostate cancer reveals dynamic tumour-immune cell communication. Cell Prolif..

[43] Blazkova J (2021). Distinct mechanisms of long-term virologic control in two HIV-infected individuals after treatment interruption of anti-retroviral therapy. Nat. Med..

[44] Board NL, Moskovljevic M, Wu F, Siliciano RF, Siliciano JD (2022). Engaging innate immunity in HIV-1 cure strategies. Nat. Rev. Immunol..

[45] Liu XY (2023). Single-cell transcriptomic analysis deciphers key transitional signatures associated with oncogenic evolution in human intramucosal oesophageal squamous cell carcinoma. Clin. Transl. Med..

[46] Watanabe M, Moon KD, Vacchio MS, Hathcock KS, Hodes RJ (2014). Downmodulation of tumor suppressor p53 by T cell receptor signaling is critical for antigen-specific CD4(+) T cell responses. Immunity.

[47] Bassez A (2021). A single-cell map of intratumoral changes during anti-PD1 treatment of patients with breast cancer. Nat. Med..

[48] Wang Z (2021). Thymosin alpha-1 has no beneficial effect on restoring CD4+ and CD8+ T lymphocyte counts in COVID-19 patients. Front. Immunol..

[49] Wang Y (2020). Target-triggered “signal-off” electrochemical aptasensor assisted by Au nanoparticle-modified sensing platform for high-sensitivity determination of circulating tumor cells. Anal. Bioanal. Chem..

[50] Zhu X (2021). A disposable gold foil paper-based aptasensor for detection of enteropathogenic Escherichia coli with SERS analysis and magnetic separation technology. Mikrochim. Acta.

[51] Liao L (2021). Highly stable surface-enhanced Raman spectroscopy assay on abnormal thrombin levels in the blood plasma of cancer patients. Anal. Methods.

[52] Gohring JT, Fan X (2010). Label free detection of CD4+ and CD8+ T cells using the optofluidic ring resonator. Sensors.

[53] Chen J (2015). Microfluidic impedance flow cytometry enabling high-throughput single-cell electrical property characterization. Int. J. Mol. Sci..

[54] Wang M (2022). Developments of conventional and microfluidic flow cytometry enabling high-throughput characterization of single cells. Biosensors.

[55] Chang D (2023). A high-dimensional microfluidic approach for selection of aptamers with programmable binding affinities. Nat. Chem..

[56] Yang K (2023). A functional group-guided approach to aptamers for small molecules. Science.

[57] Gao Y (2022). A lysosome‐activated tetrahedral nanobox for encapsulated siRNA delivery. Adv. Mater..

[58] Yang F, Li Q, Wang L, Zhang GJ, Fan C (2018). Framework-nucleic-acid-enabled biosensor development. ACS Sens..

[59] Bui H (2018). Localized DNA hybridization chain reactions on DNA origami. ACS Nano.

[60] Fu X (2020). Size-selective molecular recognition based on a confined DNA molecular sieve using cavity-tunable framework nucleic acids. Nat. Commun..

[61] Wu Q (2022). Multibranched linear DNA-controlled assembly of silver nanoclusters and their applications in aptamer-based cell recognition. ACS Appl. Mater. Interfaces.

[62] Zhang T (2023). Nanomaterials targeting toll‐like receptor 4 prevent bisphosphonate‐related osteonecrosis of the jaw via regulating mitochondrial homeostasis in macrophages. Adv. Funct. Mater..

[63] Chen R (2022). Treatment effect of DNA framework nucleic acids on diffuse microvascular endothelial cell injury after subarachnoid hemorrhage. Cell Prolif..

[64] Yan R (2023). Typhaneoside-tetrahedral framework nucleic acids system: mitochondrial recovery and antioxidation for acute kidney injury treatment. ACS Nano.

[65] Zhang B (2022). Facilitating in situ tumor imaging with a tetrahedral DNA framework‐enhanced hybridization chain reaction probe. Adv. Funct. Mater..

[66] Ma W (2022). Biomimetic nanoerythrosome-coated aptamer-DNA tetrahedron/maytansine conjugates: pH-responsive and targeted cytotoxicity for HER2-positive breast cancer. Adv. Mater..

[67] Li J (2021). DNA-based dynamic mimicry of membrane proteins for programming adaptive cellular interactions. J. Am. Chem. Soc..

[68] Shi P, Zhao N, Coyne J, Wang Y (2019). DNA-templated synthesis of biomimetic cell wall for nanoencapsulation and protection of mammalian cells. Nat. Commun..

