# Supplementary material for: DNA framework signal amplification platform-based high-throughput systemic immune monitoring
Source: Signal Transduct Target Ther. 2024 Feb 7;9:28. doi: 10.1038/s41392-024-01736-0 (PMC10847453; doi:10.1038/s41392-024-01736-0)
Supplement: Supplementary file 1 — Revised supporting information [file 41392_2024_1736_MOESM1_ESM.docx]

Supplementary Materials for

**DNA framework signal amplification platform-based high-throughput systemic immune monitoring**

Ye Chen^1^, Xingyu Chen^1^, Bowen Zhang^3^, Yuxin Zhang^1^, Songhang Li^1^, Zhiqiang Liu^1^, Yang Gao^1^, Yuxuan Zhao^1^, Lin Yan^2^, Yi Li^2^*, Taoran Tian^1^*, Yunfeng Lin^1,4^*

Correspondence to: liyiscu@outlook.com / taoran.tian@scu.edu.cn / yunfenglin@scu.edu.cn

**This PDF file includes:**

Table of Contents

Supplementary materials and methods

Supplementary Figures. S1 to S38 and respective legends

Tables S1 to S8 and respective captions

References for supplementary materials

Table of Contents

**Supplementary Fig. S1**. The step-wise truncation process and affinity analysis of CD4 aptamer.

**Supplementary Fig. S2**. The step-wise truncation process and affinity analysis of CD8 aptamer.

**Supplementary Fig. S3**. The step-wise truncation process and affinity analysis of CD14 aptamer.

**Supplementary Fig. S4**. The interaction simulation of CD4 aptamer/CD4 protein, CD8 aptamer/CD8 protein, and CD14 aptamer/CD14 protein by Maestro.

**Supplementary Fig. S5.** The interaction simulation of CD4 aptamer/CD4 protein, CD8 aptamer/CD8 protein, and CD14 aptamer/CD14 protein by HDOCK.

**Supplementary Fig. S6**. The electrophoretic mobility shift assay (EMSA) and affinity analysis of CD4, CD8 and CD14 aptamers.

**Supplementary Fig. S7**. The specificity validation of the original and truncated aptamer by EMSA.

**Supplementary Fig. S8**. The cell isolation and phenotype analysis of CD4+ T, CD8+ T lymphocytes, and PBMCs.

**Supplementary Fig. S9**. The performance of CD4 and CD8 aptamer in detecting CD4+ T, CD8+ T lymphocytes in whole blood cells.

**Supplementary Fig. S10**. The CD4+ T lymphocyte detection for ten blood samples by CD4 truncated aptamer.

**Supplementary Fig. S11.** The CD8+ T lymphocyte detection for ten blood samples by CD8 truncated aptamer.

**Supplementary Fig. S12.** The monocyte detection for ten blood samples by CD14 truncated aptamer. **Supplementary Fig. S13.** The detailed design scheme of three pairs of HCR probes for CD4, CD8, and CD14 proteins.

**Supplementary Fig. S14.** The 2% agarose gel electrophoresis (AGE) demonstrating the metastability of H1-SE and H2-SE.

**Supplementary Fig. S15.** The detailed structure design scheme of DSAP.

**Supplementary Fig. S16.** The mechanism of accelerated HCR by the spatial confinement effect of DTF.

**Supplementary Fig. S17.** The fabrication method of DSAP, including HCR probe design and DTF-structured HCR probe design.

**Supplementary Fig. S18.** The 8% polyacrylamide gel electrophoresis (PAGE) showing the step-wise construction of DSAP.

**Supplementary Fig. S19.** TEM image showing the structure of DTF-SE and DSAP.

**Supplementary Fig. S20.** The dissociation curve and KD value of CD4 Hairpin1-DTF.

**Supplementary Fig. S21.** The FCA of the influence of temperature on the HCR efficiency.

**Supplementary Fig. S22.** The AGE showing the cascade amplification capacity of DSAP.

**Supplementary Fig. S23.** The validation of CD4 expression in HuT-78 cells and U937 cells.

**Supplementary Fig. S24.** The signal changes of HuT-78 cells treated with DSAP along with time. **Supplementary Fig. S25.** The signal changes of HuT-78 cells treated with nude HCT probes along with time.

**Supplementary Fig. S26.** The validation of CD4 expression in HL-60 cells and A549 cells.

**Supplementary Fig. S27.** The confocal laser scanning microscopy (CLSM) of HL-60 cells treated with DSAP for 30 min and 60 min, respectively.

**Supplementary Fig. S28.** The colocalization analysis of the Cy5 and Alexa 488 signal in U937 cells, CD4+ T lymphocytes, and HL-60 cells treated with FAM/Cy5/BHQ-2 labeled DSAP.

**Supplementary Fig. S29.** The validation of CD4+ T cell, CD8+ T cell, and monocytes detection by DSAP in whole blood samples.

**Supplementary Fig. S30.** The CD4+ T lymphocyte detection of ten blood samples from healthy people by flow cytometry.

**Supplementary Fig. S31.** The CD4+ T lymphocyte detection of ten blood samples from HIV patients by flow cytometry.

**Supplementary Fig. S32.** The CD4+ T lymphocyte detection of ten blood samples from tumor patients by flow cytometry.

**Supplementary Fig. S33.** The CD4+ T lymphocyte detection of ten blood samples from organ transplantation patients by flow cytometry.

**Supplementary Fig. S34.** The CD4+ T lymphocyte detection of ten blood samples from hematological tumor patients by flow cytometry.

**Supplementary Fig. S35.** The CD4+ T lymphocyte detection of ten blood samples from autoimmune disease patients by flow cytometry.

**Supplementary Fig. S36.** The analysis of CD4 expression level in CD4+ T lymphocytes from the patients with different health situations and diseases.

**Supplementary Fig. S37.** The stability of the detection results based on DSAP.

**Supplementary Fig. S38.** The application of DSAP-based immune monitoring in HIV immunodeficiency staging.

**supplementary Table 1.** The sequences (5’-3’) of DSAP and aptamers in this work.

**supplementary Table 2.** The results of flow cytometry and DSAP in blood samples from the patients with HIV, tumor, organ transplantation, autoimmune diseases and organ transplantation.

**supplementary Table 3.** The consistency comparison between flow cytometry results and DSAP results.

**supplementary Table 4.** The mean signal-to-noise by DSAP when detecting blood samples with different CD4+ T lymphocyte concentration intervals.

**supplementary Table 5.** The mean signal-to-noise of blood samples from HIV patients of severe, moderate, mild immunodeficiency, and normal conditions by DSAP.

**supplementary Table 6.** The mean signal-to-noise of blood samples from HIV patients of mild period (350~500/μL CD4+ T lymphocytes) and normal period (>500/μL CD4+ T lymphocytes)

**supplementary Table 7.** The mean signal-to-noise of blood samples from HIV patients of moderate period (200~350/μL CD4+ T lymphocytes) and mild period (350~500/μL CD4+ T lymphocytes).

**supplementary Table 8.** The mean signal-to-noise of blood samples from HIV patients of severe period (0~200/μL CD4+ T lymphocytes) and moderate period (200~350/μL CD4+ T lymphocytes).

**Reference**

Materials and methods

*Materials and reagents*

The DNA oligonucleotides were synthesized from Sangon (Sangon) and were purified via PAGE or high-performance liquid chromatography (HPLC). 40% Acr-Bis (39:1) was purchased from Sangon. Ammonium persulfate (APS), N, N, N’, N’-Tetramethylethylenediamine (TEMED), 10×Tris-Borate-EDTA (TBE) buffer, 1 M Tris-HCl buffer (pH = 8.0), glucose, and agarose and magnesium chloride (MgCl2) were purchased from Sigma Aldrich with analytical grade. The DL 2000 DNA marker and 6×loading buffer were purchased from Takara, and the 10,000×Super GelRed dye was purchased from US Everbright Corporation. Dulbecco’s phosphate-buffered saline (DPBS) and DiO dye were purchased from ThermoFisher Scientific. The human CD4+ T lymphocytes isolation kit, human CD8+ T lymphocytes isolation kit, human CD4 antibody, human CD8 antibody, and human CD19 antibody were purchased from BioLegend. Milli-Q ultrapure water (18 MΩ·cm) was used for all experiments.

The buffers used in the study were prepared as follows:

TE buffer containing 10 mM Tris-HCl and 1 mM EDTA, at pH=8.0.

TM buffer containing 10 mM Tris-HCl and 10 mM MgCl2, at pH=8.0.

10×TBE buffer containing 900mM Tris-borate, 20 mM EDTA, at pH=8.0

0.5×TBE buffer containing 45 mM Tris-borate, 1 mM EDTA, at pH=8.0.

DPBS buffer was supplemented with 5 mM MgCl2 and 4.5 mg/ml glucose to form the binding buffer.

*The truncation procedure of CD4, CD8, and CD14 aptamer.*

The advent of SELEX technology has shown the ability to evolve artificial ligands with high affinity and specificity. The primary step of SELEX includes library preparation, positive selection, competitive binding, negative selection, and sequence enrichment.^1^ The key to screening the aptamer with high specificity is iteration times, competitive binding, and negative selection. First, around 20 rounds of cell-SELEX are required to achieve aptamers with the highest affinity toward the target cell line. The aptamers with high affinity and specificity have been isolated after many rounds. Second, set a cluster of scramble cells or proteins, like the natural detection environment in the competitive binding. For example, to screen CD8 aptamer, the authors set PBMCs as competitive environments to evolve higher specific aptamers.^2^ The pan T cell will be isolated using the magnetic bead separation method, and the bound aptamer will enter the subsequent selection. Third, the negative objectives should be chosen as the cells or proteins with a similar membrane composition or protein structure. The closer the positive and negative cells or proteins resemble each other, the more specific the aptamer will be. For example, to obtain CD8 aptamer, the authors chose CD4+ CD8- J.RT3-T3.5 cells as negative cells.^2^ To screen CD14 aptamer, the authors chose CD14- Mo macrophage as negative cells.^3^

The aptamer truncation is to deplete the unfunctional regions of the original aptamer and retain the active domain for providing their affinity. The identification of the functional domains, the secondary simulation, flow cytometry, and aptamer/protein interaction simulation were conducted. The secondary structures were predicted by Mfold and NUPACK software. The human CD4+, CD8+ T lymphocytes, and PBMCs were isolated, and they were treated with the Cy5-labeled CD4 aptamer, FAM-labeled CD8 aptamer, and FAM-labeled CD14 aptamer. The Cy5 and FMA fluorescence signal of cells was detected using flow cytometry. The detailed truncation procedures were performed as follows.

For CD4 aptamer truncation, we truncated 3’ primer sequences from the original CD4 and obtained CD4 T1. The 5’ primer sequences of CD4 T1 are then truncated and obtained CD4 T2. Because the active domain of aptamer mainly attributes in the loop and stem regions, we next truncated the 5’ free single strand in CD4 T2 and obtained the truncated aptamer. The secondary structure of the truncated aptamer is conserved in the truncation process, as shown in the square box. According to the FCA in Fig. 2a, the MFI of CD4+ T lymphocytes do not show significant differences among original aptamer, CD4 T1, CD4 T2, and truncated aptamer. The truncated aptamer shows a higher affinity to CD4 protein than the original aptamer with a lower *K*_D_ value. We further extracted the two stem-loop regions from the truncated aptamer to form CD4 T3 and CD4 T4. We treated isolated CD4+ T lymphocytes with Cy5 labeled CD4 T3, CD4 T4, and truncated aptamer. The FCA showed that CD4 T3 and CD4 T4 could not bind with CD4+ T lymphocytes. Therefore, we inferred that both stem-loop structures of truncated aptamer contribute to the affinity and bind with CD4 at specific orientations. Therefore, we inferred the truncated aptamer is most likely the actual active domain of the original aptamer.

For CD8 aptamer truncation, we first truncated 5’ and 3’ primer sequences from the original CD8 and obtained CD8 T1. We truncated 3’ free oligo from CD8 T1 and obtained the CD8 T2. The C-G base pair at the end of stem regions was then cut off and the truncated aptamer was obtained. The secondary structure of the truncated aptamer is conserved in the truncation process, as shown in the square box. According to the FCA in Fig. 2b, the MFI of CD8+ T lymphocytes does not show significant differences among original aptamer, CD8 T1, CD8 T2, and truncated aptamer. The truncated aptamer shows a higher affinity to CD8 protein than the original aptamer with a similar *K*_D_ value. We further truncated the A-T base pair at the end of the stem structure from the truncated aptamer and obtained CD8 T3. Due to reduced complementary pairing at stem regions, the stem structures cannot be blocked. The NUPACK and Mfold both indicated the same structure for CD8 T3. We treated isolated CD8+ T lymphocytes with FAM labeled CD8 T3 and truncated aptamer. The FCA indicated that CD4 T3 showed weaker affinity to CD8+ T lymphocytes. We inferred that the specific orientation and distance of the two stem-loop structures play a vital role in binding with CD8 protein. Due to the lack of stem region in CD8 T3, the distance and orientation of the two stem-loop structures cannot be maintained. Therefore, we inferred the truncated aptamer is most likely the actual active domain of the original aptamer.

For CD14 aptamer truncation, we truncated a portion of the stem-loop structure at the 5’ and 3’ ends and obtained the truncated aptamer. According to the FCA in Fig. 2c, the truncation process didn’t affect the affinity of aptamer to CD14 but increased the affinity instead. We continued to cut off the A-T base pair at the end of the stem from the truncated aptamer and obtained CD14 T2. Due to fewer base pairs in the stem regions, the secondary structure of CD14 T2 is different from the truncated aptamer. We removed the first and second loop regions from the truncated aptamer and obtained CD14 T3. We removed the last loop region from the truncated aptamer and obtained CD14 T4. The CD14 T3 and CD14 T4 are both the portion of truncated aptamer structure. We treated isolated human monocytes with FAM-labeled aptamer to ensure CD14 T2, CD14 T3, and CD14 T4 affinity changes. FCA demonstrated that CD14 T2, CD14 T3, and CD14 T4 hardly show any affinity with monocytes. Therefore, we inferred that all the stem-loop structures in truncated aptamer contribute the affinity to CD14. The stem regions at the end maintain the structural stability of the truncated aptamer. The truncated aptamer is the active domain of the original aptamer.

*The interaction simulation of the three doublets of aptamer/protein*

In our work, we applied both Maestro and HDOCK servers to conduct DNA/protein interaction simulations for the three doublets of aptamer/protein to obtain more reliable simulation results. Maestro software 13.5 (Schrödinger suite) has complete functions involved in DNA/protein interaction simulation, including protein preparation, nucleotide preparation, nucleotide-protein docking, and protein interaction analysis.^4-6^ Maestro software shows high accuracy and precision in interaction simulation, which has been widely applied in computer-aided drug design.^7-9^ HDOCK web server also have DNA/protein interaction simulation functions.^10-12^ The detailed simulation steps are described as follows.

First, we obtained the crystal structures of the CD4 protein (PDB ID: 7T0R Chain C) and CD14 protein (PDB ID: 4GLP Chain A) from the RCSB PDB database and extracted the target crystal structures. Due to the lack of correct crystal structures of CD8 alpha protein in the RCSB PDB database, we obtained its crystal structures via Alphafold2 (Uniprot: P01732). The structural accuracy of Alphafold2 has been proved in many works.^13-15^ The obtained protein crystals were processed using the Protein Preparation Wizard to perform the protein process, regenerate states of native ligand, H-bond assignment optimization, protein energy minimization, and remove water, respectively. Finally, we obtained the prepared proteins for subsequent interaction simulation.

Second, Mfold software was applied to stimulate the secondary structures of the aptamers corresponding to the three proteins.^16^ The secondary structures with the lowest ΔG values were chosen. We again conducted the secondary structure simulation with NUPACK software to double-check the reliability of the obtained secondary structures.^17^ The same structures of the three truncated aptamers were obtained from Mfold and NUPACK software. Next, we conducted trial-and-error testing to confirm the structures by flow cytometry. Therefore, we obtained an accurate aptamer secondary structure by computer prediction and trial-and-error testing. According to the predicted secondary structures, 3dRNA/DNA v2.0 web server (http://biophy.hust.edu.cn/3dRNA) was next used to indicate the tertiary structure of the aptamer.^18^ The Nucleotide Preparation Wizard was used to perform a series of processing on the aptamer tertiary structure, thus obtaining the prepared aptamer structure.

Third, perform DNA/protein interaction of the processed aptamer with CD4, CD8, or CD14 protein using the Glide, the Nucleotide-Protein Docking module. Set the number of ligand rotations to probe to 70000 (the highest docking number in current software) and set maximum poses to return to 30. In particular, the ligand DNA was rotated 70,000 times in multiple orientations relative to the receptor protein. The PIPER pose energy can be calculated at every DNA/protein complex, reflecting the binding free energy between the ligand and the receptor. The lower the PIPER pose energy, the higher the binding stability. Therefore, the rank of PIPER pose energy of all the DNA/protein complex produced by multiple different orientations simulation can be obtained. The DNA/protein complex ranked in the top 1000 were clustered based on the root mean square distance between the matching atoms. The DNA/protein complex with the most neighborhoods in every cluster was chosen for further optimization. Next, the residue side chains within 5Å near the interaction interface will be optimized to minimize conflicts and optimize interactions.

Forth, the optimal DNA/protein complex with the lowest PIPER pose energy was regarded as a reliable interaction between the aptamer and their corresponding proteins. To present 3D stereoscopic views of interaction structure, the different chains of DNA and protein were labeled with different colors. In addition, the Protein Interaction Analysis module was used to identify specific interaction interfaces between the aptamer and CD4 or CD8 or CD14 proteins.

Fifth, to double-check the interaction simulation's reliability, we again performed DNA/protein interaction simulations of the three doublets of aptamer/protein in the HDOCK web server. The prepared PDB files of the protein and corresponding aptamer were input, and the docking scores and interaction structure were obtained. The figures below were generated using PyMol software. According to the standard about the docking scores provided by HDOCK software, these aptamers showed significant affinity to their corresponding proteins. And the interaction structure is consistent with those from Maestro software.

*The electrophoretic mobility shift assay*

The human CD4 (P01730), CD8 alpha chain (P01732), CD14 (P08571), CD3D-CD3E heterodimer (P04234; P07766), and CD19 (P15391) recombinant proteins were purchased from MCE. The original and truncated CD4, CD8, and CD14 aptamers were modified with FAM at the 5’end of the sequences. The volume of the reaction system is 20 μL, and the concentration of aptamer, including original aptamer and truncated aptamer, is set as 500 μM. The concentration of protein varies from 0 to 1000 nM. Phosphate-buffered saline was applied as the binding buffer for all aptamer/protein interactions. After 30-min incubation at 25℃, 5 μL reaction solution was mixed with 1μL 6×DNA loading buffer (Takara). The mixtures were loaded into 1.0mm 8% polyacrylamide gel wells. The electrophoresis was performed at 80V for 80 min in 0.5×TBE buffer. After electrophoresis, the gels were exposed to detect FAM fluorescence using the FAM filter configuration (excitation at 494 nm and emission at 525 nm) of the iBright scanning system (Thermo Fisher Scientific).

To explore the specificity of the three aptamers before and after truncation, the FAM-labeled original and truncated CD4, CD8, and CD14 aptamers were treated with human CD3 and CD19 recombinant proteins at 25℃ for 30 min. The electrophoresis of the reaction solutions was conducted according to the methods mentioned above. The gels were also scanned via the FAM filter configuration of the iBright scanning system.

Due to larger molecular weight, the aptamers binding with target proteins mobile slower than nude aptamers, leading to the band shift phenomenon. To further analyze the *K*_D_ values of the aptamers, the quantification of the bound and unbound bands was conducted by ImageJ software. The bound rate can be obtained according to the formula:

Bound rate (%)= (intensity of bound aptamer) /(bound aptamer + unbound aptamer) × 100%

The three parallel experiments were carried out. The dissociation curve can be obtained: the x-axis represents protein concentration (nM), the y-axis refers to the bound rate (%). The *K*_D_ values can be further calculated according to the dissociation curve.

*The isolation of CD4+, CD8+ T lymphocytes and PBMCs*

The human CD4+ T and CD8+ T lymphocytes were isolated from whole blood using human CD4+ T cell isolation kit (Biolegend, 480010) and human CD8+ T cell isolation kit (Biolegend, 480012). First, lymphocytes were isolated from whole blood using the human lymphocyte isolation solution. The isolated lymphocytes were treated with antibody cocktails for 15 min at 4 ℃. Next, streptavidin-coated magnetic beads were added to the mixture and incubated for 15 min at 4 ℃. The CD4+ T lymphocytes or CD8+ T lymphocytes were separated by magnetic force. The isolation of monocytes from whole blood is difficult, so we isolated PBMCs using the human mononuclear isolation solution (Solarbio, P9010) to conduct the subsequent experiments. For cell phenotype analysis, the cell suspension after CD4+ T lymphocyte isolation was treated with APC-labeled CD4 antibody (Biolegend) and conducted FCA. The cell suspension after CD8+ T lymphocyte isolation was treated with APC-labeled CD8 antibody (Biolegend) and conducted FCA.

*The detection of CD4+, CD8+ T lymphocytes and monocytes in clinical blood samples based on truncated aptamer*

The blood samples were obtained from the Department of Laboratory Medicine in West China Hospital and were approved by standard ethical protocols. The blood samples were coded based on the sample reception orders. The 20 tubes of blood samples were selected randomly. CD4+, CD8+ T lymphocytes, and monocytes were detected by fluorescence antibody and truncated aptamer simultaneously. The ten blood samples were used to detect CD4+ and CD8+ T lymphocytes. The ten blood samples were used for the detection of monocytes. The concentration of CD4+ T, CD8+ T lymphocytes, and monocytes in blood samples was confirmed by fluorescence antibody. The fluorescence antibody-based detection is conducted according to clinical standards. For aptamer detection, the same red blood cell lysis procedure was conducted. For CD4+ T lymphocyte and CD8+ T lymphocyte detection, the cell suspension was treated with Cy5-labeled CD4 truncated aptamer and FAM-labeled CD8 truncated aptamer for 30 min. The cell suspension was treated with the FAM-labeled CD14 truncated aptamer for monocyte detection for 30 min. The 100 uL cell suspension was detected, and the Cy5+ and FAM+ cell numbers were calculated by flow cytometry. The detected cell number by flow cytometry reflects the cell concentration in whole blood. The linear relationship between the number decided by fluorescence antibody and truncated aptamer was analyzed.

*The metastability of H1-SE and H2-SE*

The H1-SE was mixed with H2-SE at the final concentration of 500 nM at 25 ℃ for 30 min. The H1-SE, H2-SE, and mixture of H1-SE and H2-SE were loaded into 2% agarose gel wells. The electrophoresis is performed at 120 V for 30 min in 0.5×TBE buffer. The agarose gel was scanned with iBright scanning system.

*The 8% PAGE*

The 8% PAGE is conducted to characterize the step-wise synthesis of DSAP. The 1mm thick 8% polyacrylamide gel was made using 4.2 ml ultrapure water, 1.2 ml 40% Acr-bis, 60 μL 10% APS, 600 μL 10×TBE and 6 μL TEMED. 1 μL S1-SE was mixed with 1 μL S2-SE. Next, the mixture was heated to 95 ℃ and annealed to 4 ℃ to synthesize S1-SE+S2-SE. The S1-SE+S2-SE+S3 is synthesized according to the same procedures. The H1-SE and DTF-SE of the equal molar ratio were mixed together and were incubated at 25 ℃ for 10 min to synthesize the Hairpin1-DTF. The S1-SE, S1-SE+S2-SE, S1-SE+S2-SE+S3, DTF, H1-SE, H2-SE, Hairpin1-DTF, DSAP, and 20 bp DNA ladder were loaded into the wells of gels. Electrophoresis was performed at 80V for 80 min in 0.5×TBE buffer buffer. The gel was stained with 1×GelRed staining solution and was exposed by iBright scanning system.

*TEM*

For characterizing the morphology of DTF-SE and DSAP, 10 μL samples were mixed with 10 μL phosphotungstic acid for 5 min. The 10 μL mixtures were dropped onto the copper grid, and the samples were dried with nitrogen for 15 min for the examination by TEM (Libra 200, Zeiss, Oberkochen). The sizes of samples in TEM images were calculated by ImageJ software. The three parallel measurements were carried out.

*The binding assays of Hairpin1-DTF.*

To calculate the binding constant, *K*_D_ values of Hairpin1-DTF, we treated isolated 1×10^5^ CD4+ T lymphocytes with 0, 5, 10, 25, 50, 100, 200 nM Cy5/BHQ-2 labeled Hairpin1-DTF at 25 ℃ for 30 min. Flow cytometry detected the Cy5 signal of CD4+ T lymphocytes, and the mean fluorescence intensity of 1×10^4^ CD4+ T lymphocytes can be analyzed according to three parallel experiments. The dissociation curve can be obtained according to FCA, and the KD value can be calculated.

*The influence of temperature on the HCR efficiency*

1×10^5^ CD4+ T lymphocytes were treated with 200 nM DSAP for 30 min at 4 ℃, 25 ℃ and 37 ℃. The mean fluorescence intensity of CD4+ T lymphocytes was analyzed by flow cytometry. The three parallel experiments were carried out.

*The validation of cascade amplification reaction of DSAP*

The 500 nM initiator was treated with Cy5/BHQ-2 labeled DSAP of increasing molar weight for 30 min at 25 ℃. The molar weight ratios between the initiator and DSAP vary from 1:1 to 6:1. The mixtures were loaded into 2% agarose gel wells, and the electrophoresis was performed at 120V for 30 min in 0.5×TBE buffer.

*The validation of CD4 expression in positive and negative cells.*

To validate the CD4 expression level in different cells, we treated positive cells, including HuT-78 cells and U937 cells as well as negative cells, including HL-60 cells and A549 cells with 5 μL APC-labeled antibody. After 30-min incubation, the cells were washed with DPBS and centrifuged at 1000 rpm twice. The cell pellet was resuspended, and the APC signal was detected with flow cytometry.

*The CD4 expression level of CD4+ T lymphocytes of patients with different healthy conditions.*

The blood samples from healthy people, patients with HIV, tumors, organ transplantation, hematological tumors, and autoimmune diseases were collected. The whole blood cells were treated with red blood cell lysis buffer for 10 min. The whole blood cells were washed with DPBS and centrifuged at 500 g for 5 min twice. The 5 μL APC-labeled CD4 antibody was added into cell suspension and waited for 30 min. After washing twice with DPBS, the cells were detected with flow cytometry. The mean fluorescence intensity in the Cy5 channel was analyzed for every sample. The three repetitive measurements by flow cytometry for every sample were conducted.

*The validation of DSAP detection performance based on confocal laser scanning microscopy*

To explore the cascade amplification capacity of DSAP, a total of 1×10^6^ isolated CD4+ T lymphocytes were treated with 50 nM Hairpin1-DTF, 50 nM DSAP, 100 nM DSAP, 150 nM DSAP, and 200 nM DSAP. The CD4 DSAP was modified with Cy5 and BHQ-2. After incubation at 25 ℃ for 30 min, the cells were fixed with 10% formaldehyde solution at 4 ℃ for 30 min. Next, the cell suspension was precipitated via centrifuging at 500 g for 5 min. The cells were resuspended with 20 µL binding buffer and dropped onto the glass slides. CLSM (FV 3000, Olympus) was applied to observe the cell in the Cy5 channel. To compare the detection efficiency of nude HCR probe and DSAP, we treated 1×10^6^ HuT-78 cells with 200 nM DSAP and nude HCR probes for 30 min and 60 min. As a negative control, 1×10^6^ HL-60 cells were treated with 200 nM CD4 DSAP for 30 min and 60 min. The cells were treated with DiO staining buffer at 25℃ for 10 min and washed with binding buffer twice. The cell was precipitated, resuspended, fixation, and observed by CLSM in the FITC channel and Cy5 channel based on the same method. To validate the structural stability of DSAP, the HuT-78 cells, CD4+ T lymphocytes, and HL-60 cells were treated with FAM/Cy5/BHQ-2 DSAP at 25 ℃ for 30 min. CLSM observed the cells in the FAM channel and Cy5 channel. To compare the performance of DSAP and nude HCR probes in detecting CD4+ and CD8+ T lymphocytes, the isolated CD4+ T lymphocytes were treated with Cy5/BHQ-2 labeled CD4 DSAP and nude HCR probes. Similarly, CD8+ T lymphocytes were treated with FAM/BHQ-1 labeled CD8 DSAP and nude HCR probes for 30 min at 25 ℃. The cells were fixed with 10% formaldehyde solution and observed by CLSM in Cy5 and FAM channels. To explore CD4 and CD8 DSAP detection performance in whole blood samples, we treated the whole blood cells after red blood cell lysis with CD4 and CD8 DSAP for 30 min. The cells were fixed with 10% formaldehyde and observed by CLSM in Cy5 and FAM channels.

*The stability of the detection results based on DSAP*

The immuno-trol cells (6607077, Beckman Coulter) and immuno-trol low cells (B25700, Beckman Coulter) are the universal regents for detection quality control. The blood cell number remains unchanged during the expiration date. Therefore, we used immuno-trol cells and immuno-trol low cells to validate the stability of detection results by DSAP. 100 uL of immuno-trol cells and immuno-trol low cells were treated with red blood cell lysis buffer for 10 min, and 100 uL of cell suspension was added into the microwell and was treated with 20 µL of 1.2 µM CD4 DSAP at 25 ℃ in the dark for 30 min. The microwell fluorescence intensity was measured by the microplate reader (Varioskan Lux, Thermo Fisher Scientific). Every sample testing by DSAP was repeated in at least three parallel experiments. The measurement is repeated by the same method every day for 7 days. The CD4+ T lymphocyte concentration can be calculated according to signal-to-noise.

*Statistical Analysis*

All data analysis in this experiment was conducted using Origin and Prism GraphPad software. Quantitative results that met the assumption of normal distribution were described using mean and standard deviation. The numbers of samples and technical replicates have been indicated in the figure captions. Differences between groups were analyzed using two-tailed Student’s t-test (two groups), one-way analysis of variance (above two groups), and Tukey’s analysis (above two groups). Two-way ANOVA is used to investigate the influence of two or more factors on a continuous outcome variable. Bland-Altman analysis was performed to assess the consistency of the detection methods. ROC curves were used to assess the diagnostic accuracy of DSAP in immunodeficiency staging for HIV patients.

**
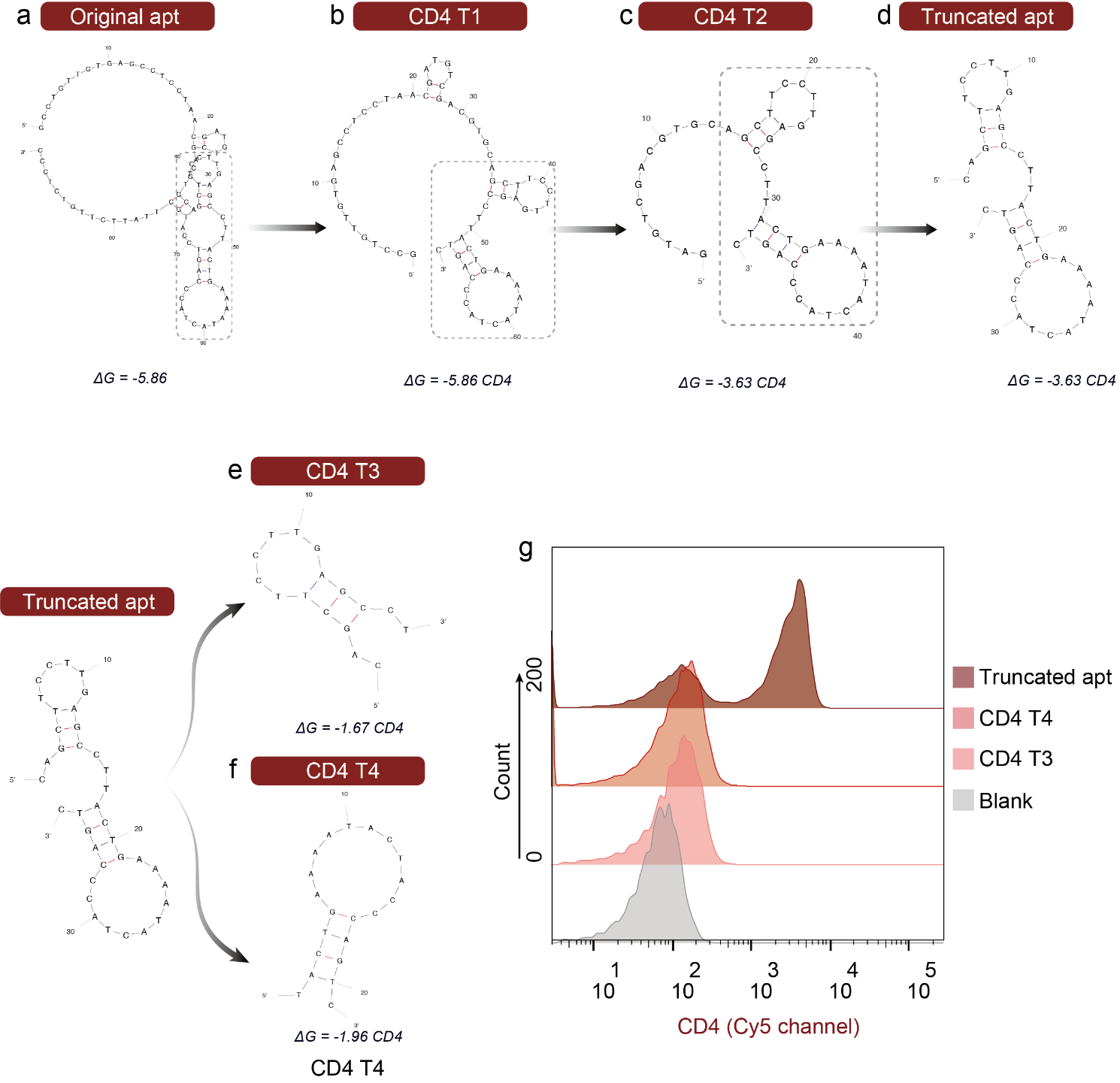
**secondary structure of the original aptamer (a), CD4 T1 (b), CD4 T2 (c), truncated aptamer (d), CD4 T3 (e) and CD4 T4 (f) are shown in the figure. g) These aptamers were modified with Cy5 at the 5' end. The human CD4+ T lymphocytes were treated with these aptamers and analyzed with flow cytometry. The flow cytometry analysis (FCA) of these aptamer treatment groups in the Cy5 channel was conducted.


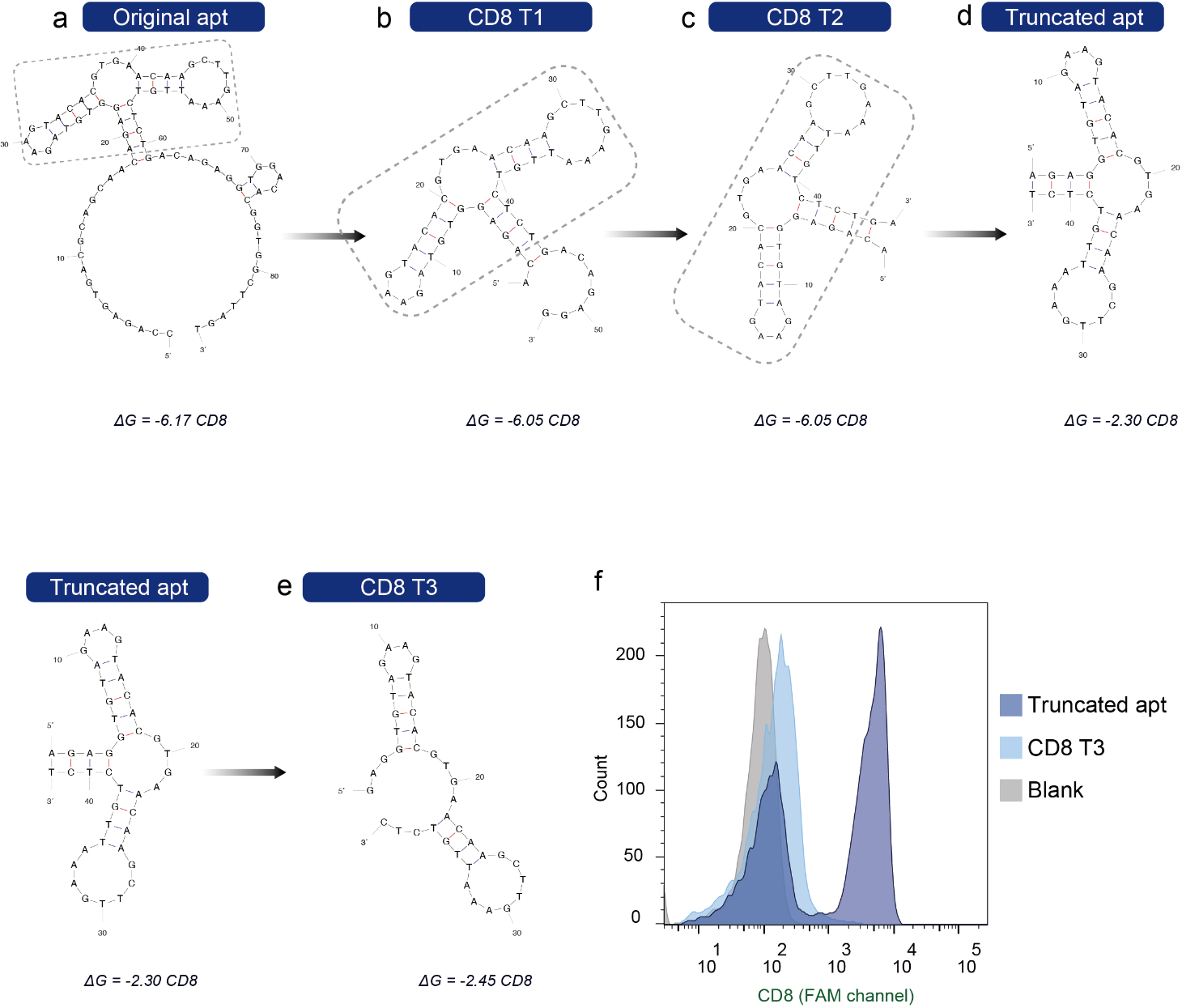
**Supplementary Fig. S2**. **The step-wise truncation process and affinity analysis of CD8 aptamer.** The secondary structures of the original aptamer (a), CD8 T1 (b), CD8 T2 (c), truncated aptamer (d), and CD8 T3 (e) are shown in the figure. f) These aptamers were modified with FAM at the 5’ end. Next, the human CD8+ T lymphocytes were treated with these aptamers and analyzed with flow cytometry. The FCA of these aptamer treatment groups in the FAM channel was conducted.


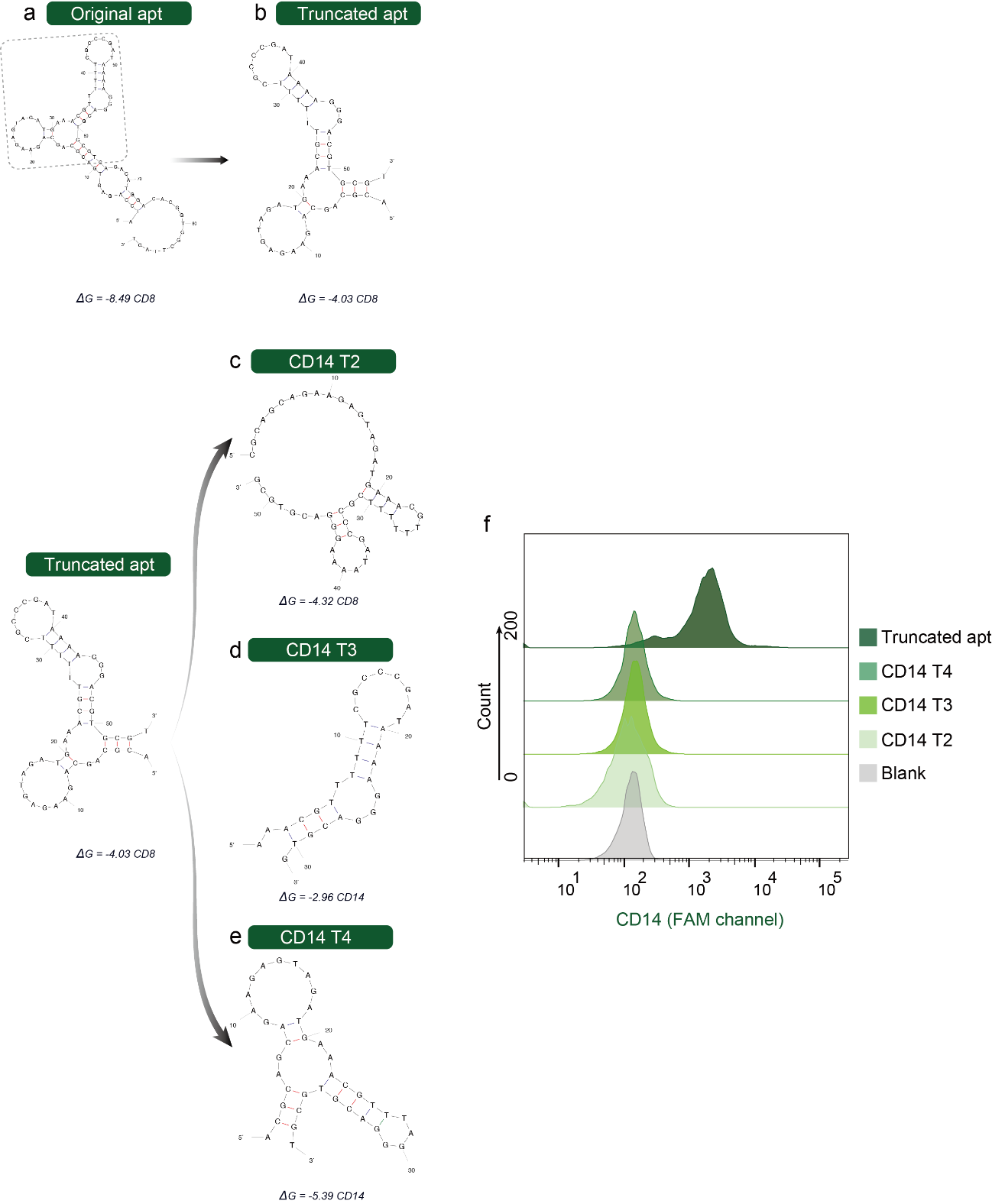
**Supplementary Fig. S3**. **The step-wise truncation process and affinity analysis of CD14 aptamer.** The secondary structure of the original aptamer (a), truncated aptamer (b), CD14 T2 (c), CD14 T3 (d), and CD14 T4 (e) are shown in the figure. f) These aptamers were modified with FAM at the 5' end. Next, the human peripheral blood mononuclear cells (PBMC) lymphocytes were treated with these aptamers and analyzed with flow cytometry. The FCA of these aptamer treatment groups in the FAM channel was conducted.

**
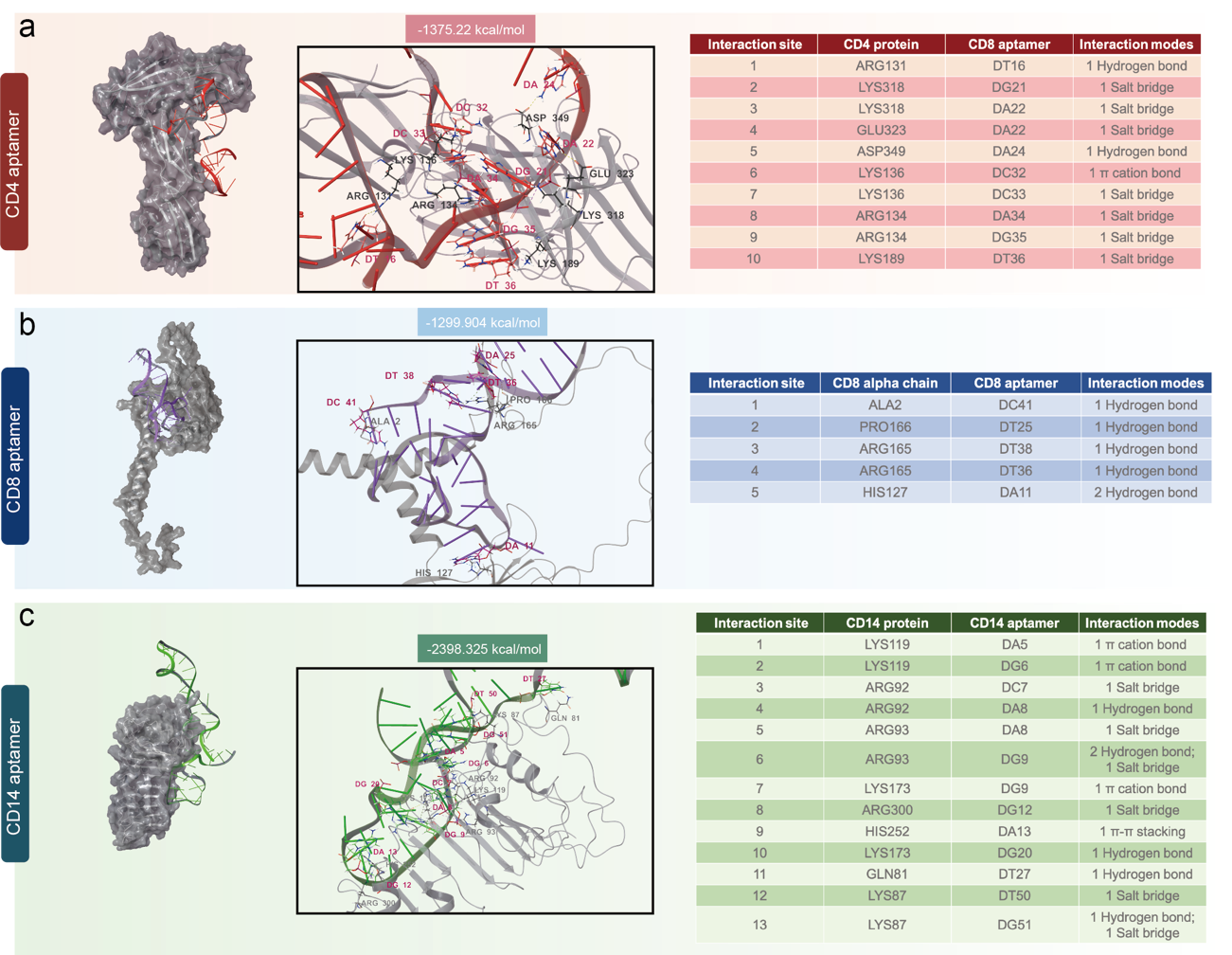
Supplementary Fig. S4**. **The interaction simulation of CD4 aptamer/CD4 protein, CD8 aptamer/CD8 protein, and CD14 aptamer/CD14 protein by Maestro.** a) The 3D structure, interaction sites, and interaction modes of interaction simulation between CD4 protein and CD4 truncated aptamer. b) The 3D structure, interaction sites, and interaction modes of interaction simulation between CD8 protein and CD8 truncated aptamer. c) The 3D structure, interaction sites, and interaction modes of interaction simulation between CD14 protein and CD14 truncated aptamer.

**
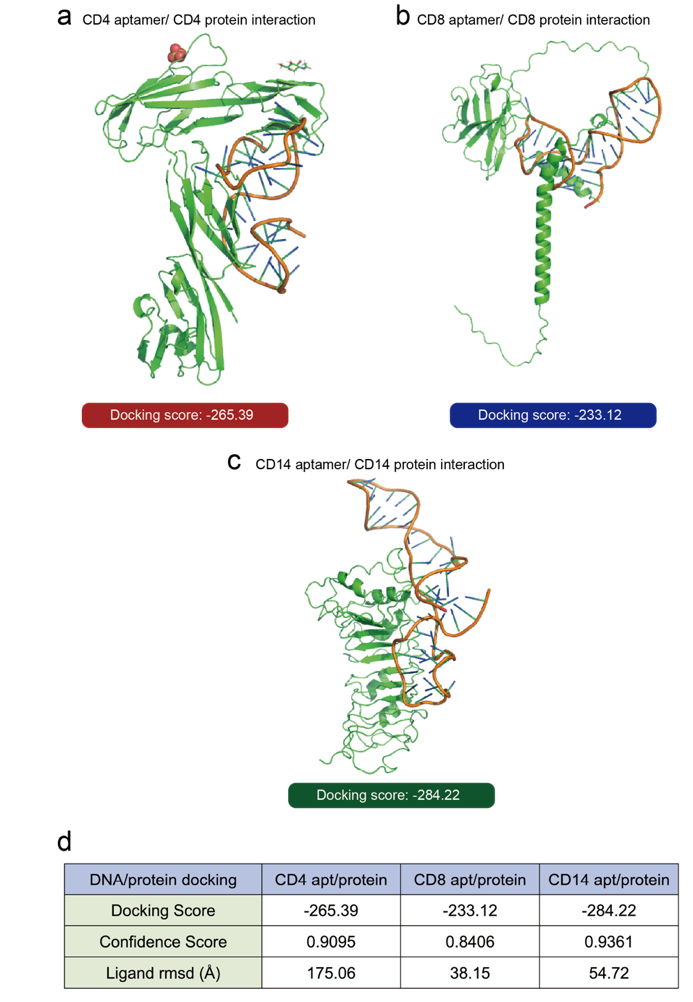
Supplementary Fig. S5. The interaction simulation of CD4 aptamer/CD4 protein, CD8 aptamer/ CD8 protein, and CD14 aptamer/CD14 protein by HDOCK.** The HDOCK is used to simulate the aptamer/protein interaction. The 3D structures of the three aptamer/protein complexes are presented. The docking scores are shown under the 3D structure. a) The interaction between CD4 truncated aptamer and CD4 protein. b) The interaction between CD8 truncated aptamer and CD8 protein. c) The interaction between CD14 truncated aptamer and CD14 protein. d) The parameter of the three aptamer/protein interaction. Docking scores are calculated by the knowledge-based iterative scoring function ITScorePP or ITScorePR. A more negative docking score means a more possible binding model. A docking score-dependent confidence score to indicate the binding likeliness of two molecules as follows,

Confidence_score = 1/[1.0+e^0.02*(Docking_Score+150)^]

Roughly, when the confidence score is above 0.7, the two molecules would be very likely to bind; when the confidence score is between 0.5 and 0.7, the two molecules would be possible to bind; when the confidence score is below 0.5, the two molecules would be unlikely to bind. The ligand RMSDs are calculated by comparing the ligands in the docking models with the input or modeled structures.

**
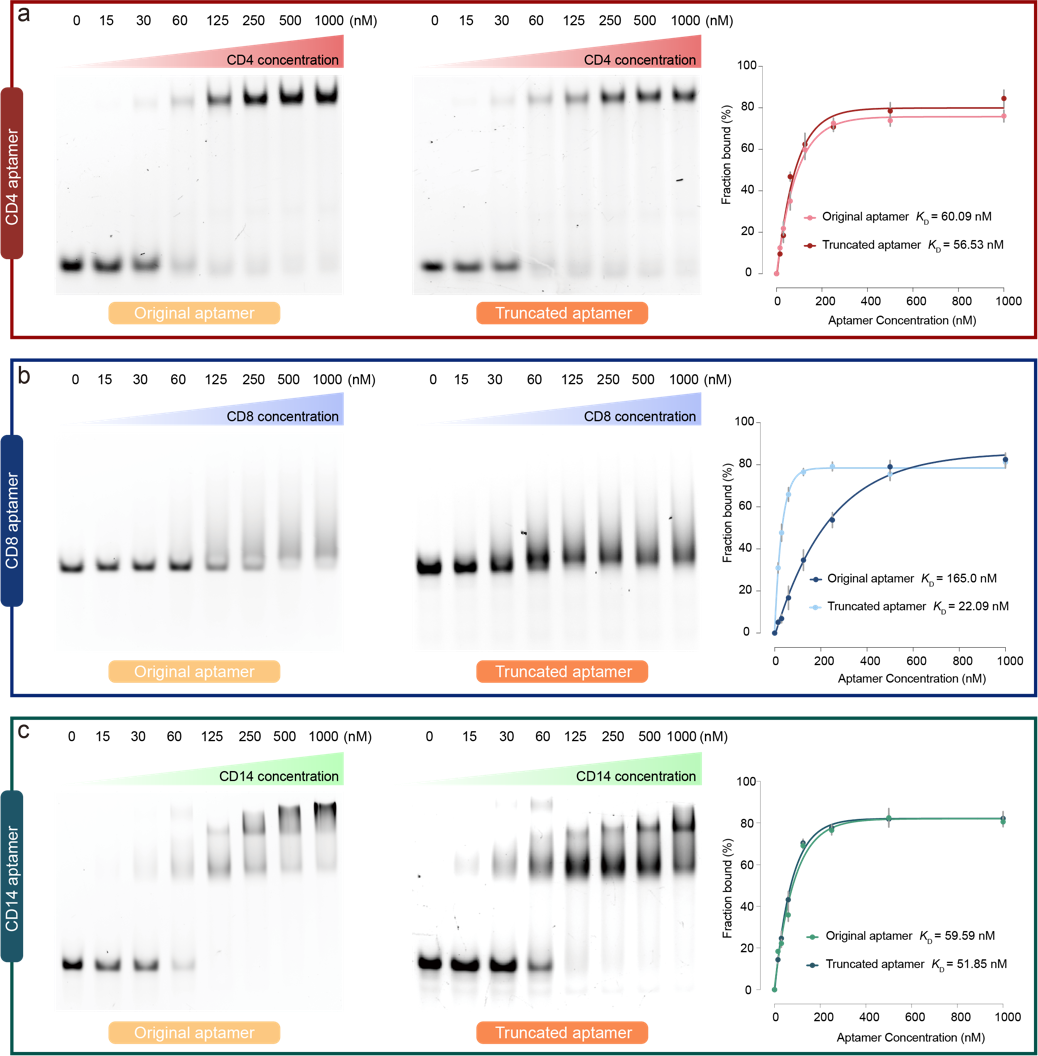
**

**Supplementary Fig. S6**. **The electrophoretic mobility shift assay (EMSA) and affinity analysis of CD4, CD8 and CD14 aptamers.** The EMSA results of CD4 aptamer/CD4 protein interaction (a), CD8 aptamer/CD8 protein interaction (b), and CD14 aptamer/CD14 protein (c). The up-shifted bands in gel images represent the aptamer binding with the target protein. Due to the larger molecular weight, the electrophoretic mobility shift is slower than the nude aptamer. The protein concentration in the reaction system increased gradually from the left to the right lane. The dissociation curves and *K*_D_ values calculated by the EMSA results of the original and truncated aptamer were displayed on the right. Three parallel experiments decided the SD values.


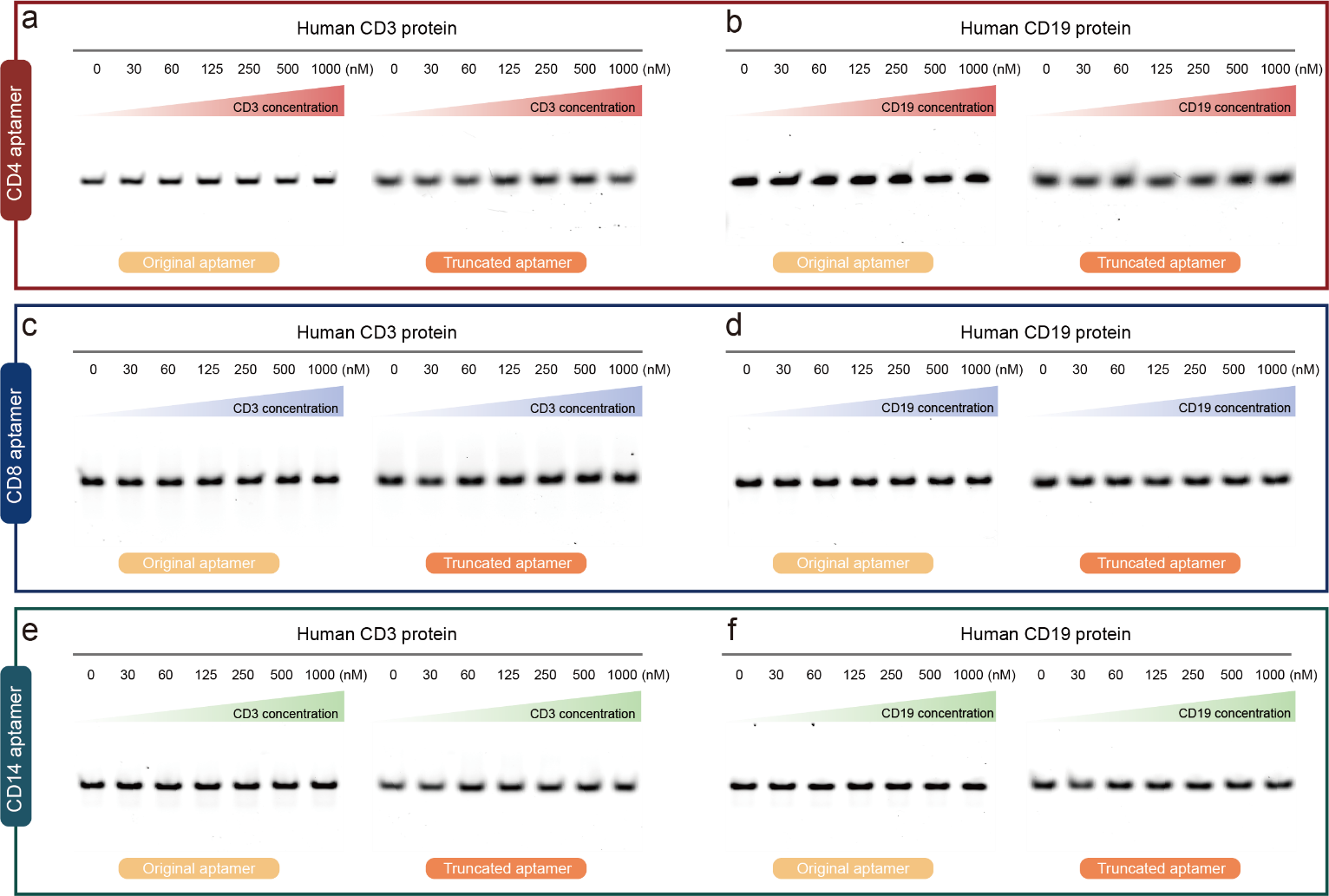
**Supplementary Fig. S7**. **The specificity validation of the original and truncated aptamer by EMSA.** EMSA is used to demonstrate the interaction between aptamers and negative proteins. The protein concentration increased from the left side to the right side of the electrophoresis lanes. a) The EMSA of the reaction products between CD3 protein and CD4 aptamer. b) The EMSA of the reaction products between CD19 protein and CD4 aptamer. c) The EMSA of the reaction products between CD3 protein and CD8 aptamer. d) The EMSA of the reaction products between CD19 protein and CD8 aptamer. e) The EMSA of the reaction products between CD3 protein and CD14 aptamer. f) The EMSA of reaction products between CD19 protein and CD14 aptamer. The band shift phenomena didn't occur in these electrophoresis gel images.


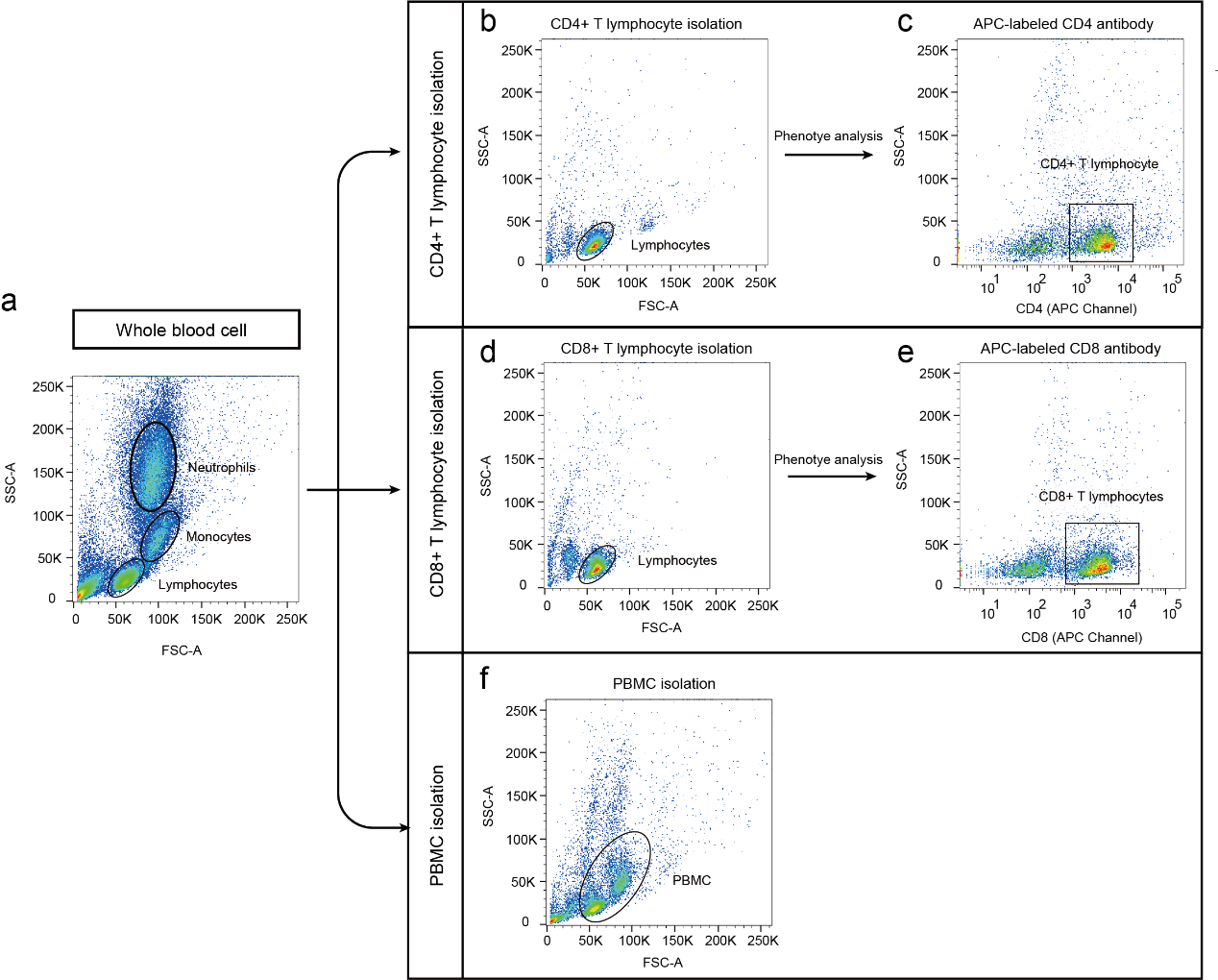
**Supplementary Fig. S8**. **The cell isolation and phenotype analysis of CD4+ T, CD8+ T lymphocytes, and PBMCs.** a) The whole blood cells. The three gated cell subsets represent lymphocytes, monocytes, and neutrophils. b) The obtained cells after CD4+ T lymphocyte isolation. c) The CD4 phenotype analysis of the isolated cells using APC-labeled CD4 antibody. The gated cell subset represents the CD4-expressing cells. d) The obtained cells after CD8+ T lymphocyte isolation. e) The CD8 phenotype analysis of the isolated cells using APC-labeled CD8 antibody. The gated cell subset represented the CD8-expressing cells. f) The gated cell subset represented PBMCs.


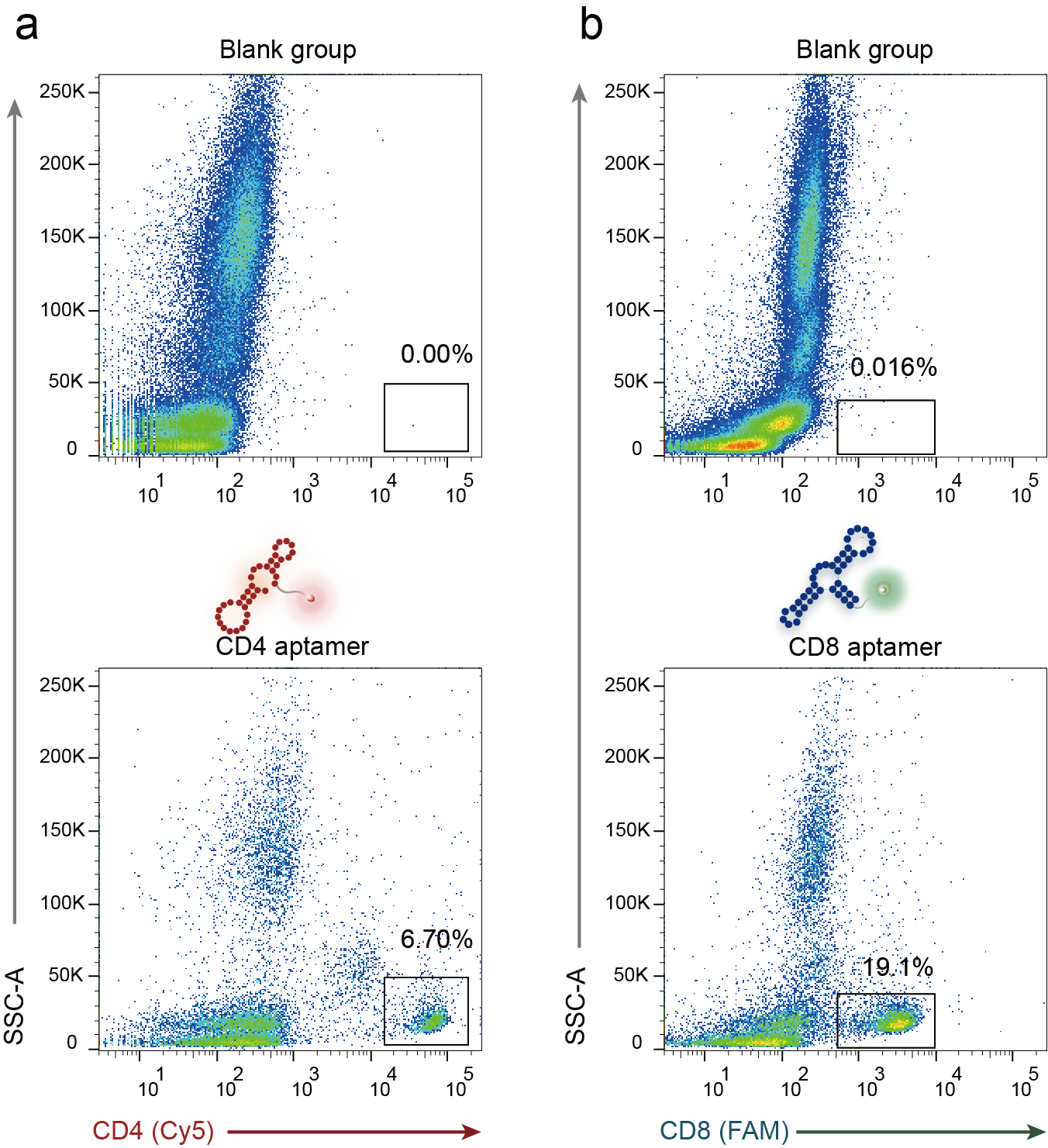
**Supplementary Fig. S9**. **The performance of CD4 and CD8 aptamer in detecting CD4+ T, CD8+ T lymphocytes in whole blood cells.** The CD4 aptamer is labeled with Cy5. The CD8 aptamer is labeled with FAM at the 5’end. a) The dot figure, gating strategy, and cell ratios of the bank group and CD4 aptamer group are shown. b) The dot figure, gating strategy, and gated cell ratios of the bank group and CD8 aptamer group are shown.


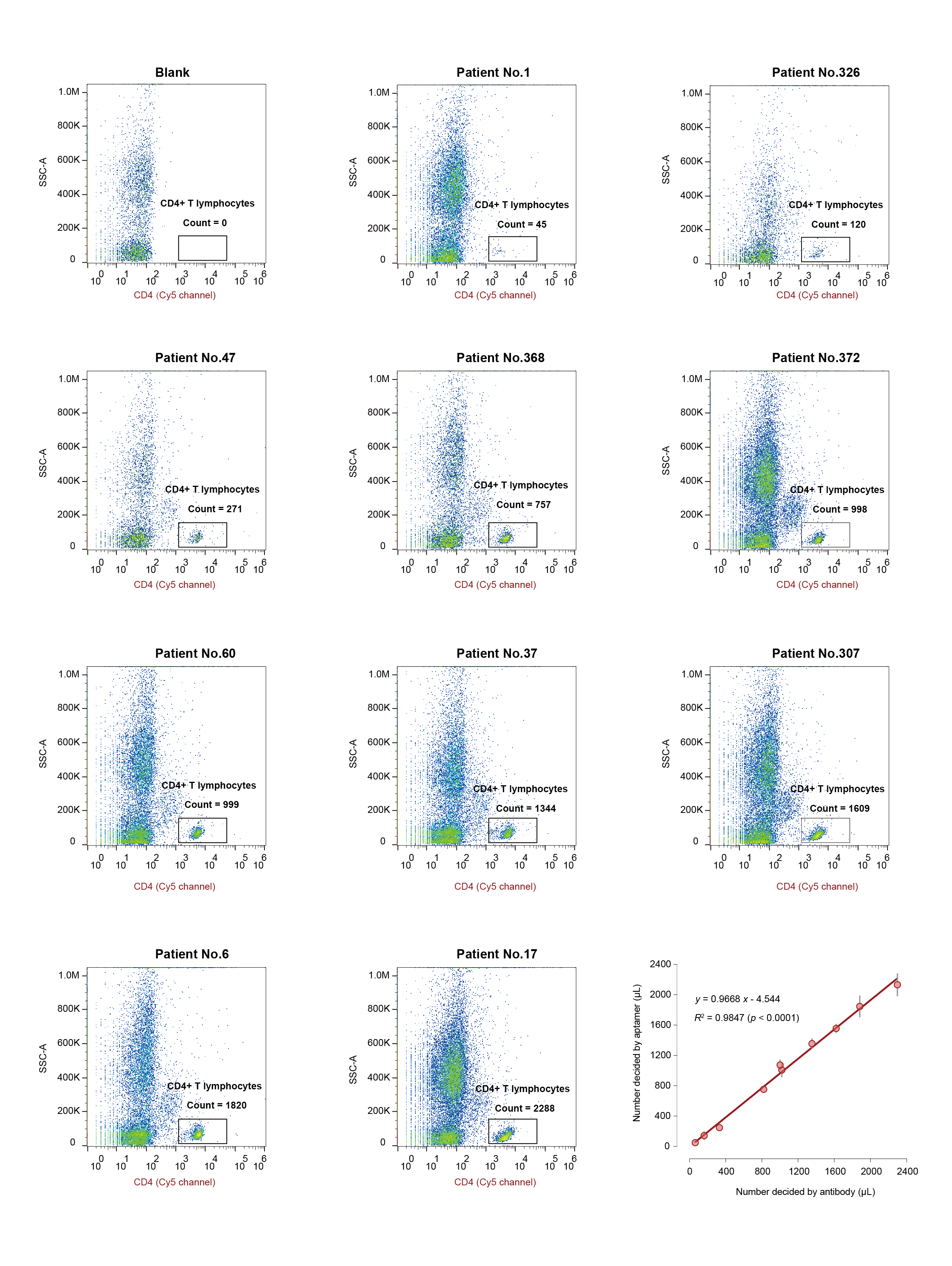
**Supplementary Fig. S10**. **The CD4+ T lymphocyte detection for ten blood samples by CD4 truncated aptamer**. The CD4 truncated aptamer is modified with Cy5 at the 5’ end. The FCA of the samples from the blank group and ten patients are shown. The linear relationship analysis of the number decided by antibody and aptamers was conducted. The relationship coefficient *R*^2^ and equation were calculated according to the detection results. The three parallel experiments determined Ths SD values. The number code is named according to the sequence of obtaining samples.


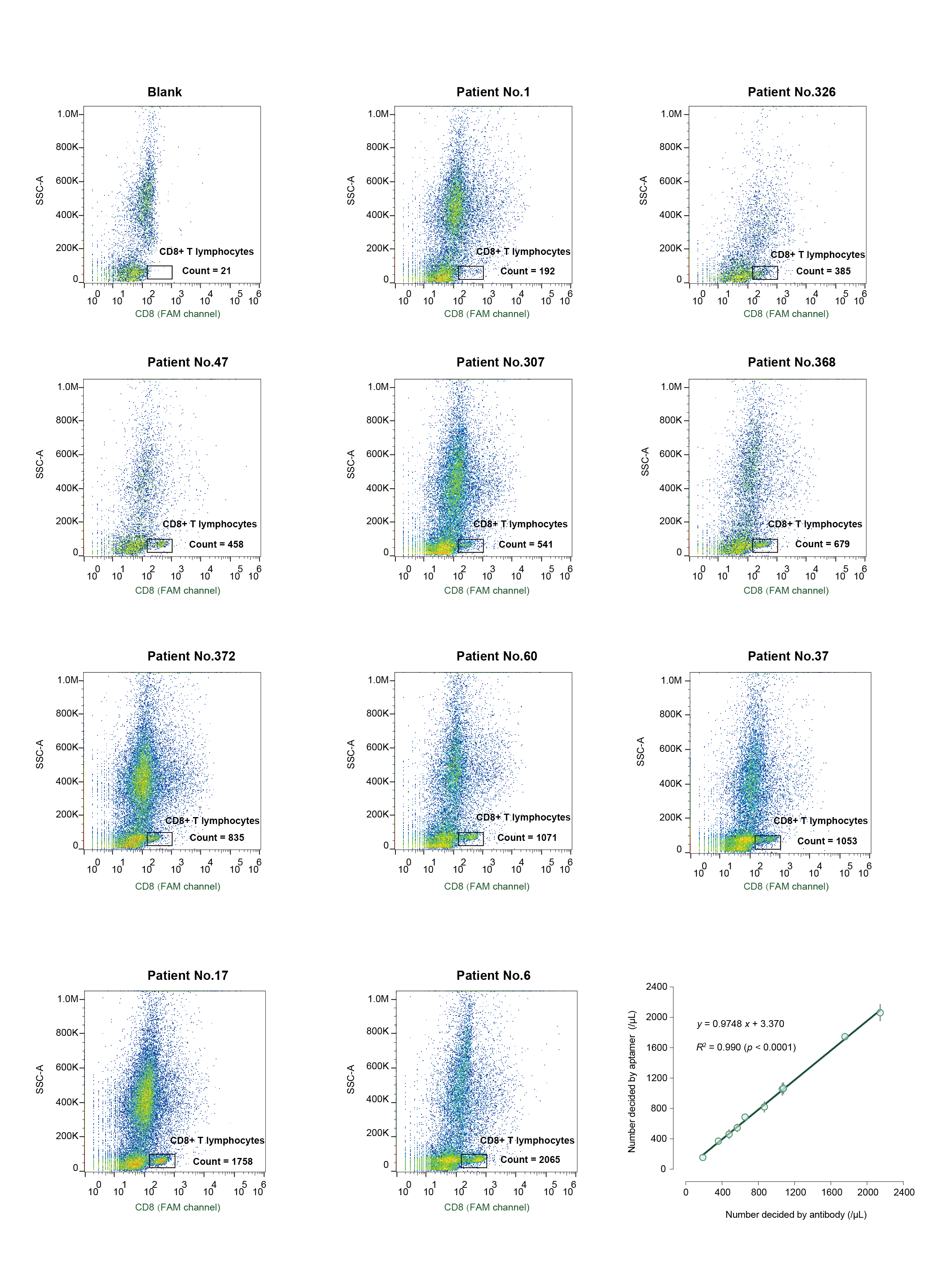
**Supplementary Fig. S11. The CD8+ T lymphocyte detection for ten blood samples by CD8 truncated aptamer.** The CD8 truncated aptamer is modified with FAM at the 5’ end. The FCA of the samples from the blank group and ten patients are shown. The linear relationship analysis of the number decided by antibody and aptamers was conducted. The relationship coefficient *R*^2^ and equation were calculated according to the detection results. Ths SD values were decided by the three parallel experiments. The number code is named according to the sequence of obtaining samples.


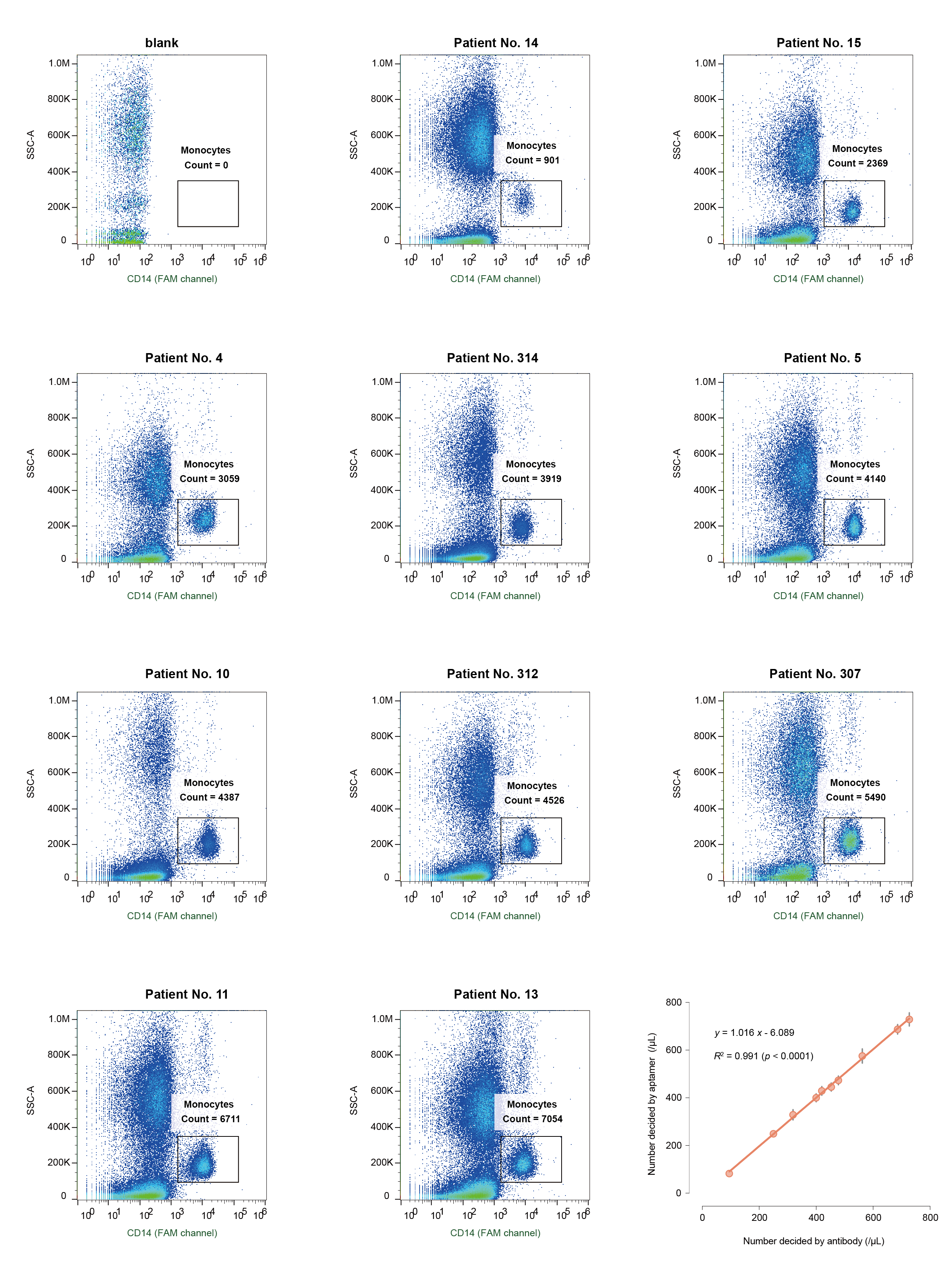
**Supplementary Fig. S12.** **The monocyte detection for ten blood samples by CD14 truncated aptamer.** The CD14 truncated aptamer is modified with FAM at the 5’ end. The FCA of the samples from the blank group and ten patients are shown. The linear relationship analysis of the number decided by antibody and aptamers was conducted. The relationship coefficient *R*^2^ and equation were calculated according to the detection results. The three parallel experiments determined Ths SD values. The number code is named according to the sequence of obtaining samples.


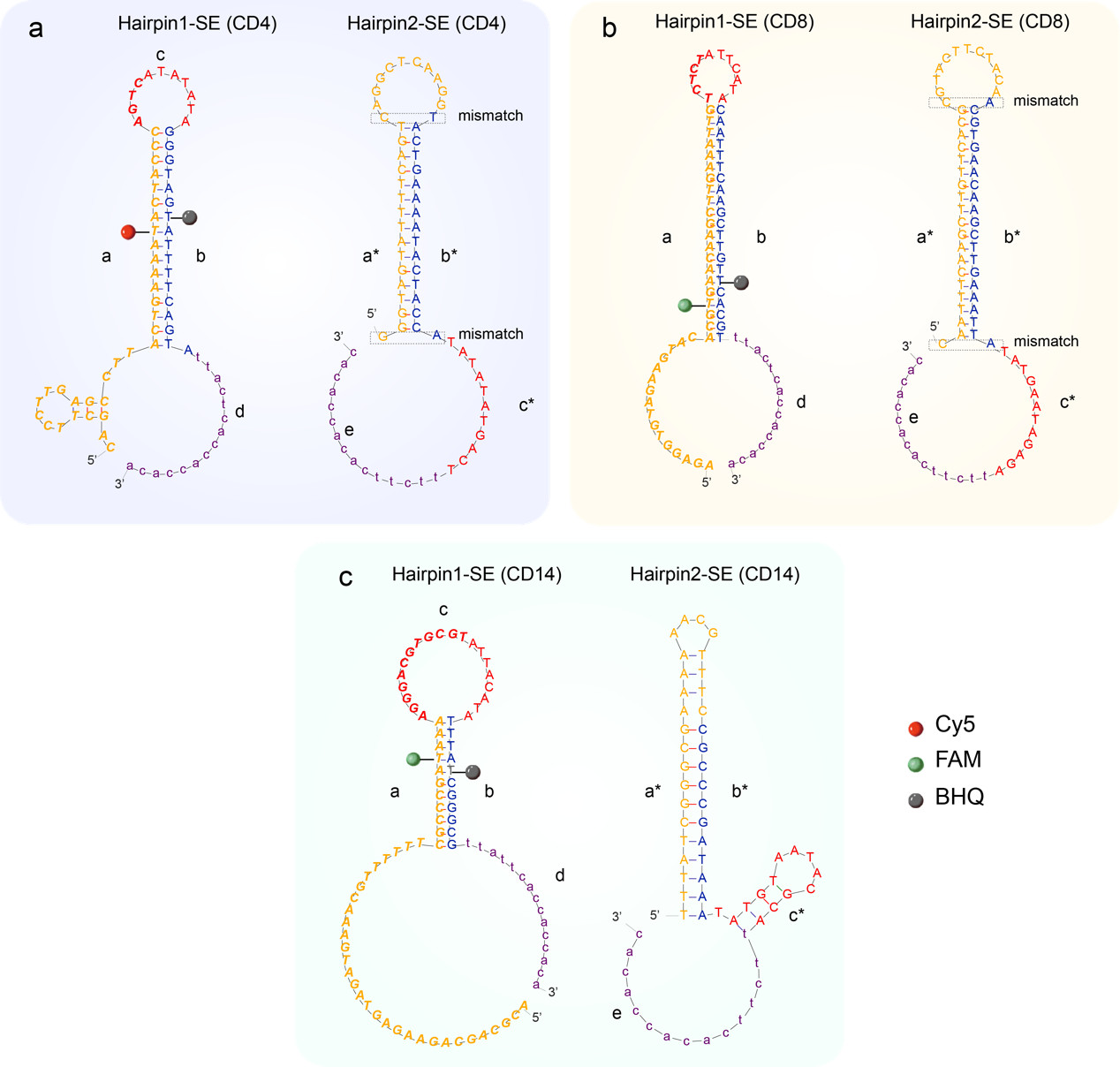


**Supplementary Fig. S13. The detailed design scheme of three pairs of HCR probes for CD4 (a), CD8 (b), and CD14 proteins (c).** The a, b, c, d, and e represented the sequences in the H1-SE and H2-SE, corresponding to the adjacent colorized regions, respectively. The * labeled letters represent the sequences, which are reversely complementary to their corresponding letter sequences. The sequences with italic and bold letters represented the truncated aptamers. The d and e regions represented the sticky ends, which are reversely complementary to S1-SE and S2-SE. The square-gated bases represent mismatch positions in H2-SE. The fluorophore and quencher, including Cy5/BHQ-2 and FAM/BHQ-1, were modified onto the bases of the stem regions, as the figure shows. The CD4 HCR probe was modified with Cy5 and BHQ-2 in the stem regions of H1-SE. The CD8 and CD14 HCR probe was modified with FAM and BHQ-1 in stem regions of H1-SE.


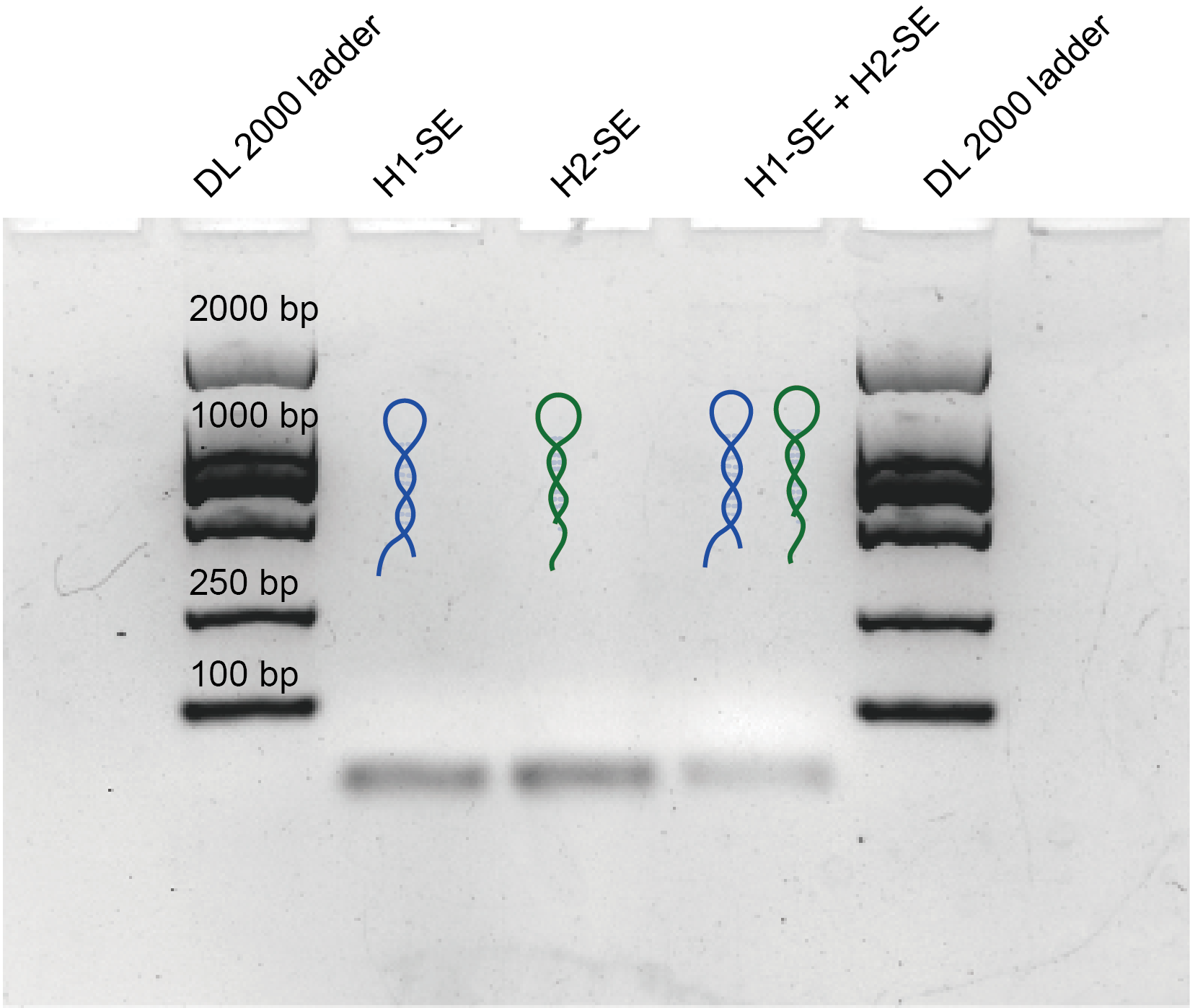
**Supplem entary Fig. S14.** **The 2% agarose gel electrophoresis (AGE) demonstrating the metastability of H1-SE and H2-SE.** The first panel to the last panel represented DL 2000 ladder, H1-SE, H2-SE, H1-SE + H2-SE, and DL 2000 ladder. The band of H1-SEs + H2-SE didn’t show any band shift or smear phenomenon, demonstrating the excellent metastability of H1-SE and H2-SE.


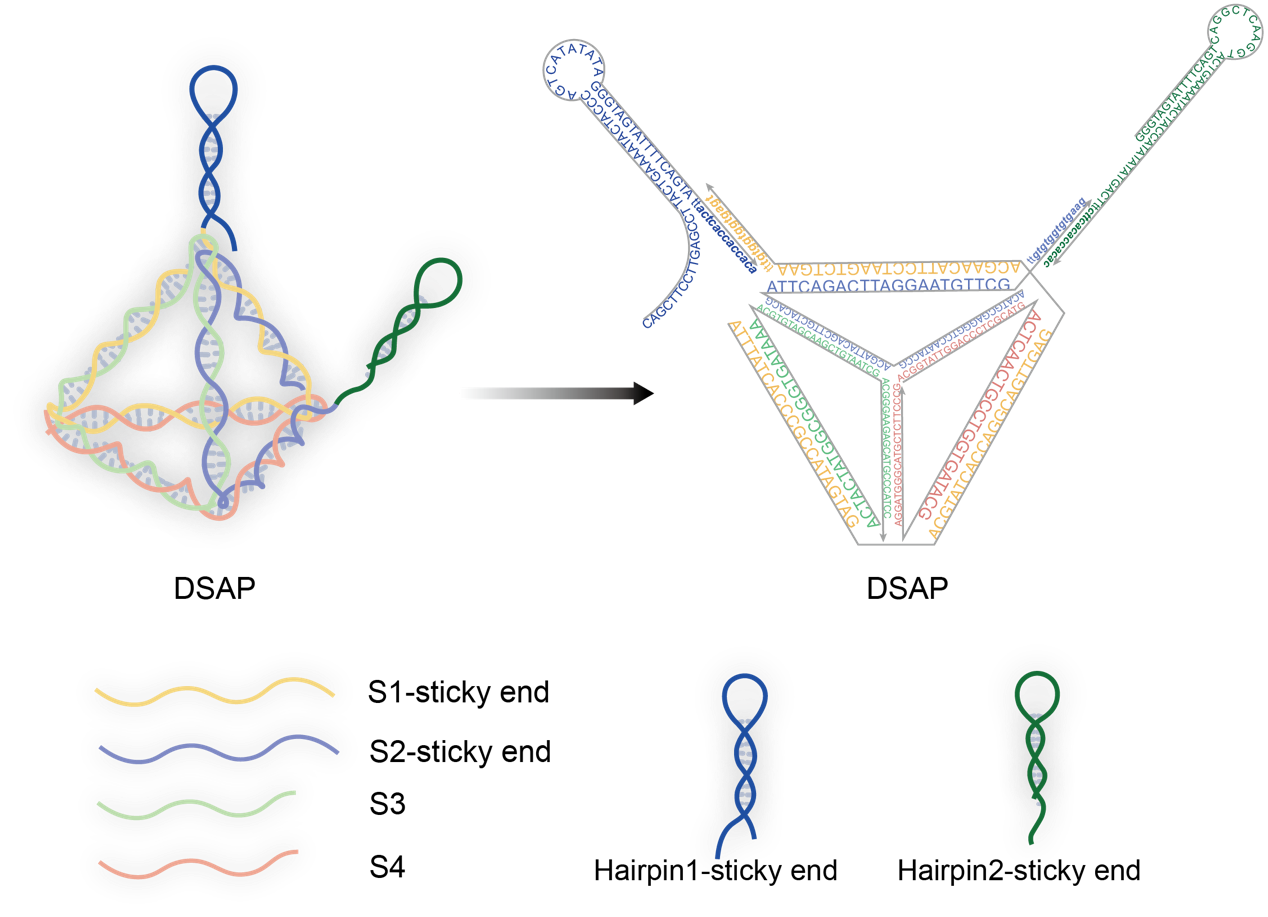
**Supplementary Fig. S15. The detailed structure design scheme of DSAP.** The DTF is synthesized from four single strands, including S1-SE, S2-SE, S3 and S4. The H1-SE and H2-SE are connected to the vertex of DTF via sticky ends. The sticky ends of H1-SE and H2-SE are different and reversely complementary to S1-SE and S2-SE.


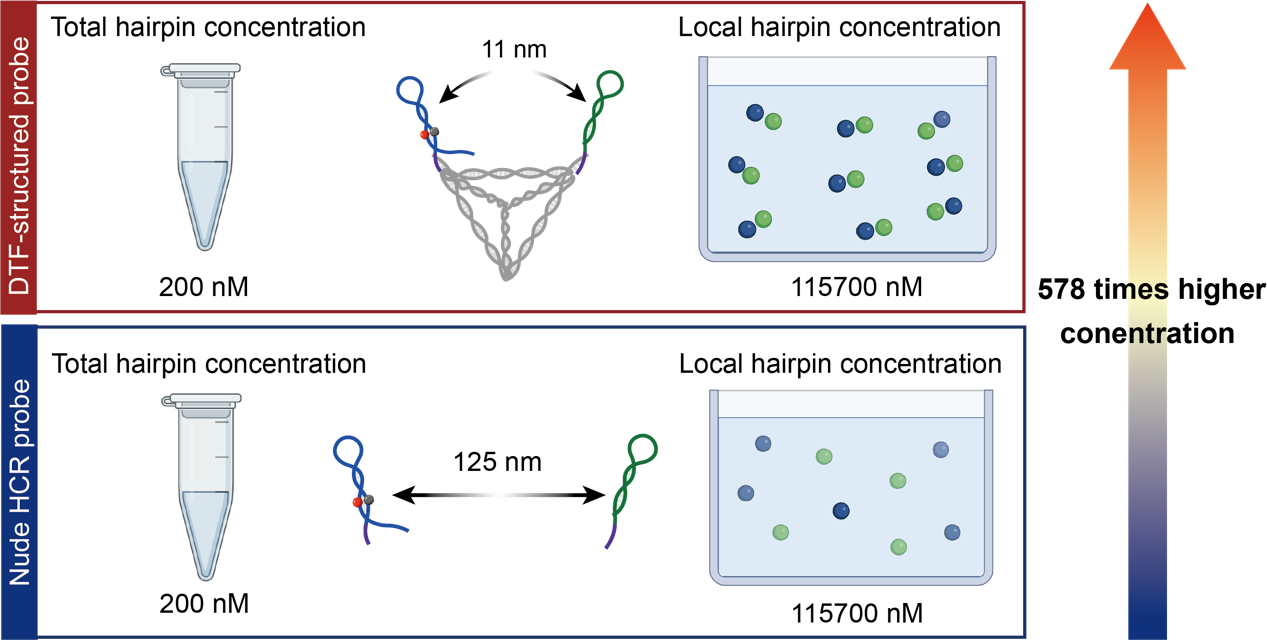
**Supplementary Fig. S16. The mechanism of accelerated HCR by the spatial confinement effect of DTF.** For the nude HCR probe in 200 nM solution, the volume of a sphere with both H1 and H2 was calculated to be 8.3 × 10 ^–18^ L with a radius of 125 nm, according to the collision theory equation. By contrast, the distance between H1 and H2 in the DSAP was about 11 nm (DTF skeleton and sticky ends). Based on the formula. The local hairpin concentration of the DSAP was 115.7 µM, 578 times higher than that of single-dispersed nude HCR probes (200 nM). Upon binding the aptamer to the target protein and subsequent unlocking of H1-SE, the adjacent H2-SE instantly hybridized with H1-SE via toehold-mediated strand displacement reaction, further driving the cascade polymerization of more DSAP to achieve signal amplification, which improved the HCR speed significantly.


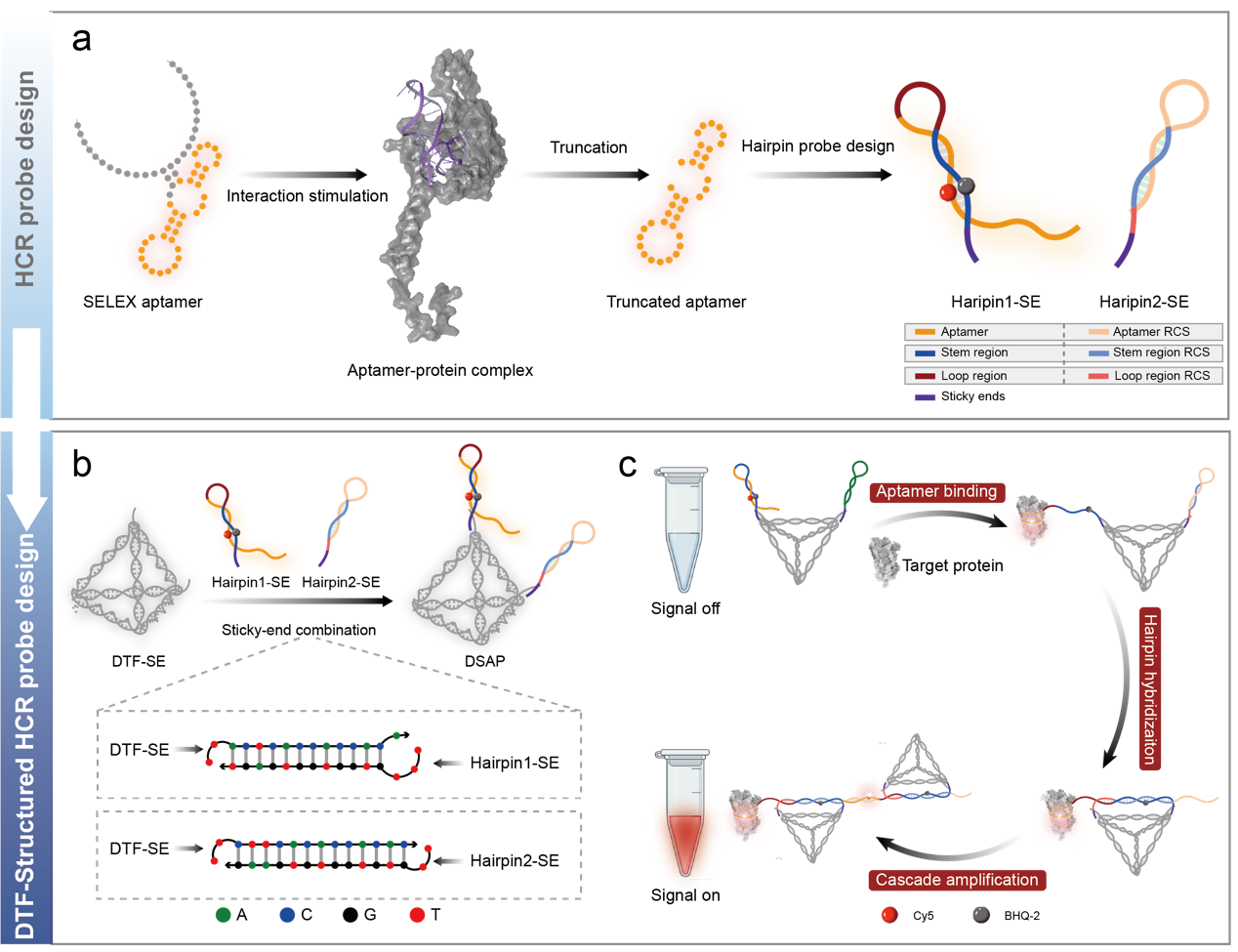
**Supplementary Fig. S17. The fabrication method of DSAP, including HCR probe design and DTF-structured HCR probe design.** a) The SELEX produced the original aptamer. The interaction simulation and secondary structure prediction of aptamer confirm the active domain. The truncation is conducted. The truncated aptamer is embedded into H1-SE partial regions. The complete H1-SE and H2-SE can be fabricated according to the HCR probe design principle. The highlighted yellow-labeled region represents the truncated aptamer. b) The H1-SE and H2-SE are connected into the vertex of DTF via sticky ends. The docking predictions between sticky ends of DTF-SE and H1-SE/H2-SE by NUPACK are shown. c) The reaction mechanism of DSAP in detecting target protein. The DSAP cannot release fluorescence due to quencher. When detecting the target protein, aptamer in H1-SE will bind with the target protein, instantly trigger hybridization of H1-SE and H2-SE, and lead to cascade amplification of DSAP. The HCR of DSAP can achieve signal amplification, increasing detection sensitivity.


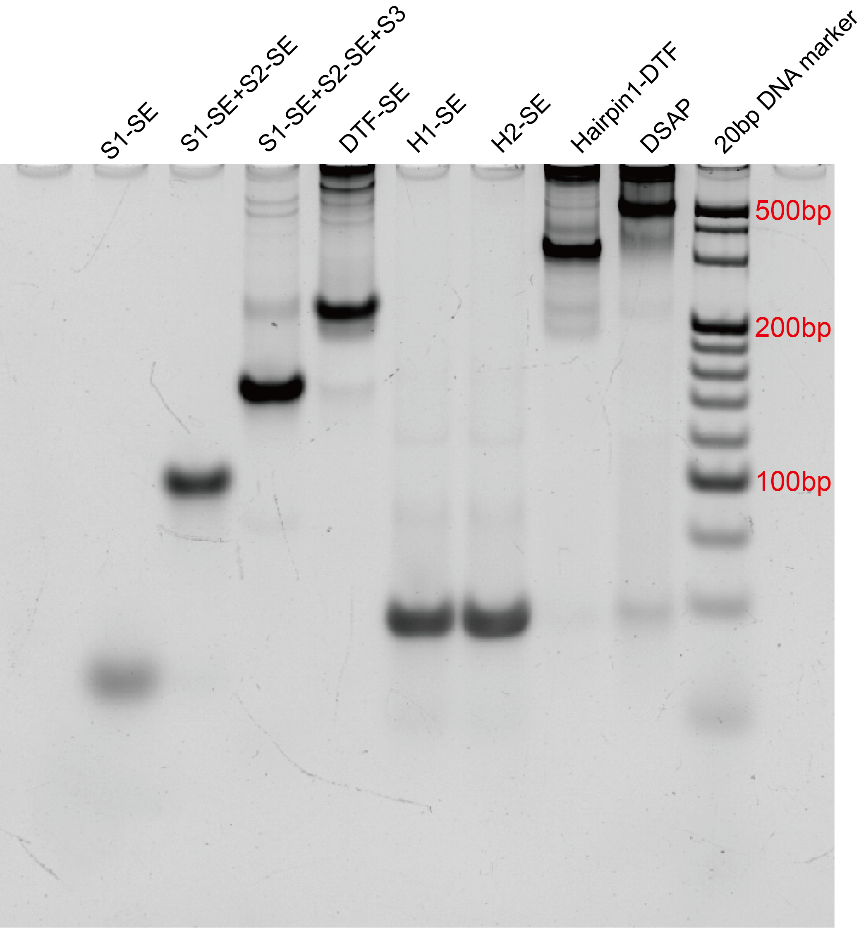
**Supplementary Fig. S18.** **The 8% polyacrylamide gel electrophoresis (PAGE) showing the step-wise construction of DSAP.** The lanes from the left side to the right side are S1-SE, S1-SE+S2-SE, S1-SE+S2-SE+S3, DTF, H1-SE, H2-SE, Hairpin1-DTF, DSAP, and 20 bp DNA ladder.


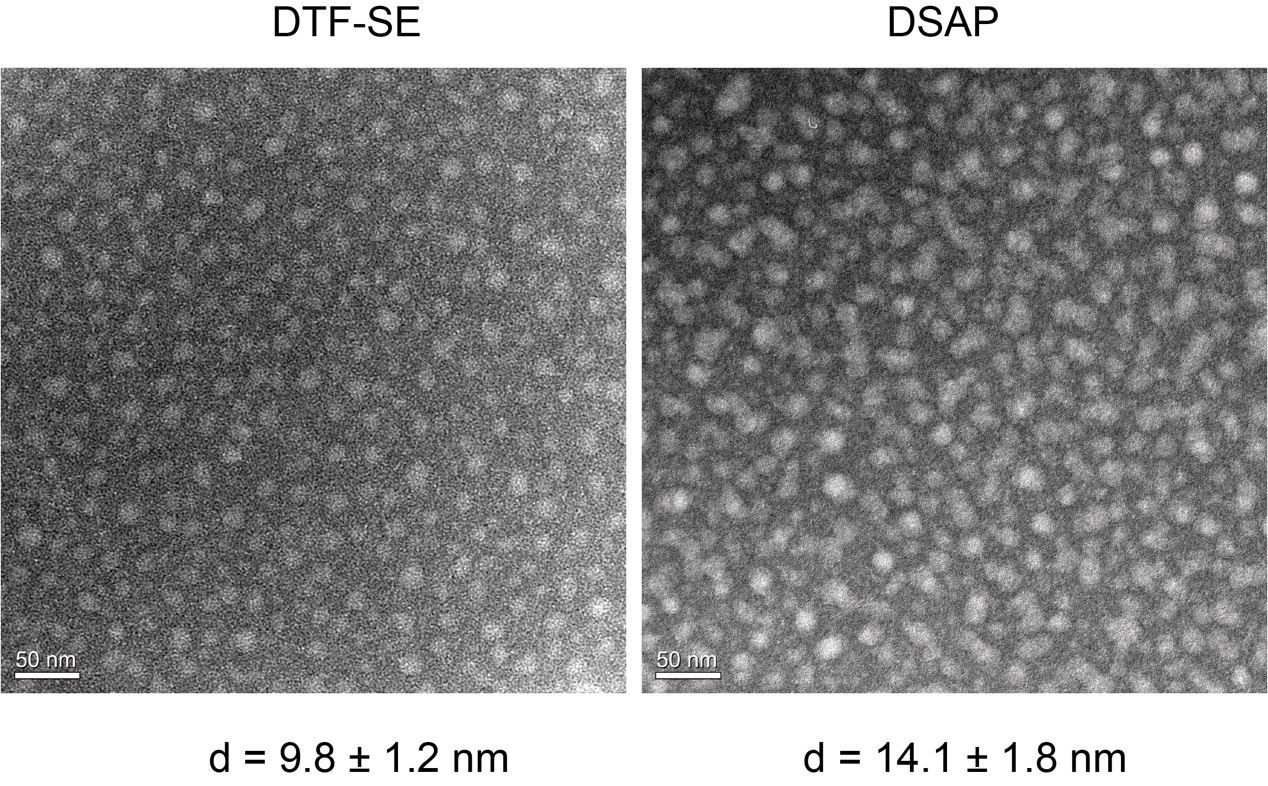
**Supplementary Fig. S19. TEM image showing the morphology of DTF-SE and DSAP.** The mean particle size of DTF-SE and DSAP was calculated by ImageJ software as 9.8 ± 1.2 nm (DTF-SE) and 14.1 ± 1.8 nm (DSAP). The size changes indicated the successful synthesis of DSAP. The scale bar is 50 nm.

**Supplementary Fig. S20. The dissociation curve and *K*_D_ value of CD4 Hairpin1-DTF.** The MFI of CD4+ T lymphocytes treated with Hairpin1-DTF is decided by flow cytometry. The *K*_D_ value of Hairpin1-DTF was 24.34 nM, according to the dissociation curve. The error bars represent means ± SD (n = 3).


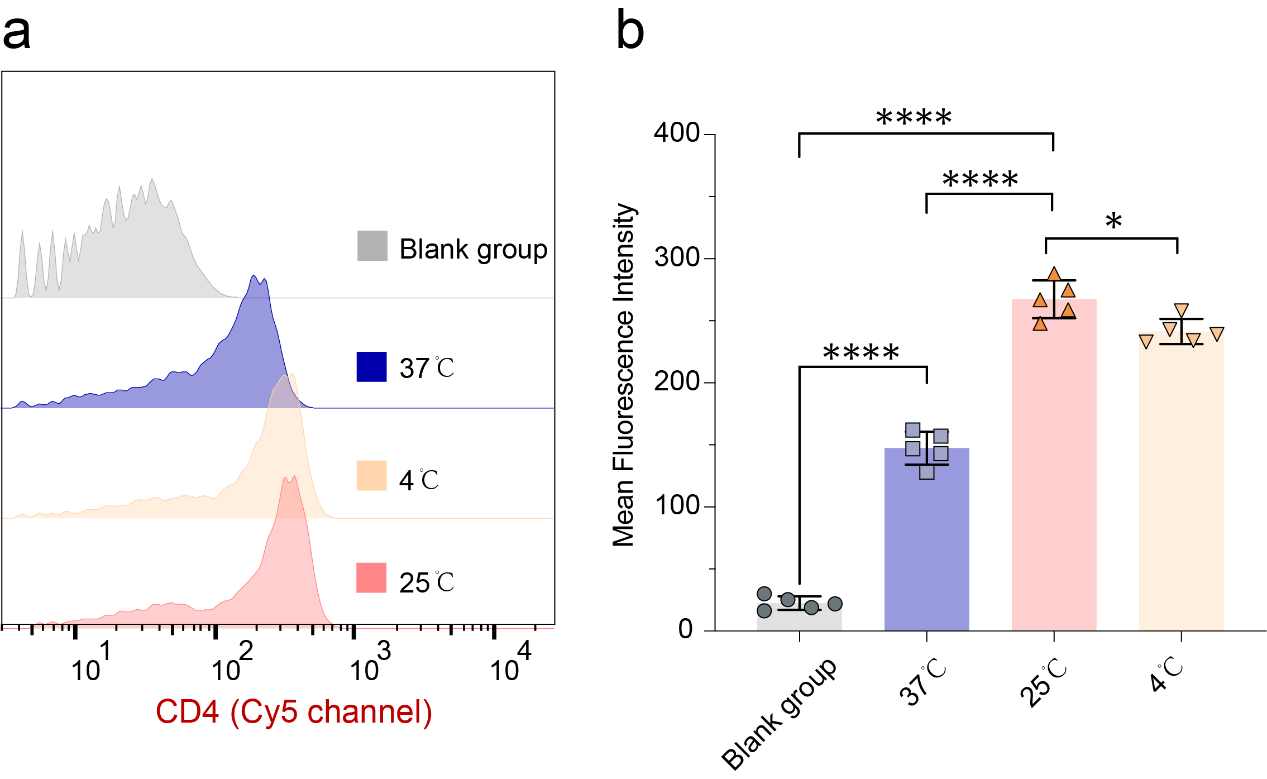
**Supplementary Fig. S21. The FCA of the influence of temperature on the HCR efficiency**. a) The flow cytometry of blank group and different reaction temperature groups on detection efficiency of DSAP. The CD4+ T lymphocytes were treated with DSAP at 4, 25, and 37 ℃. The CD4+ T lymphocytes were analyzed by flow cytometry. b) The statistical analysis of mean fluorescence intensity was decided by flow cytometry of blank and different temperature groups. The SD of the mean fluorescence intensity from 5 parallel experiments defines the error bars. The **** represented *p* < 0.0001, * represented *p* < 0.05.


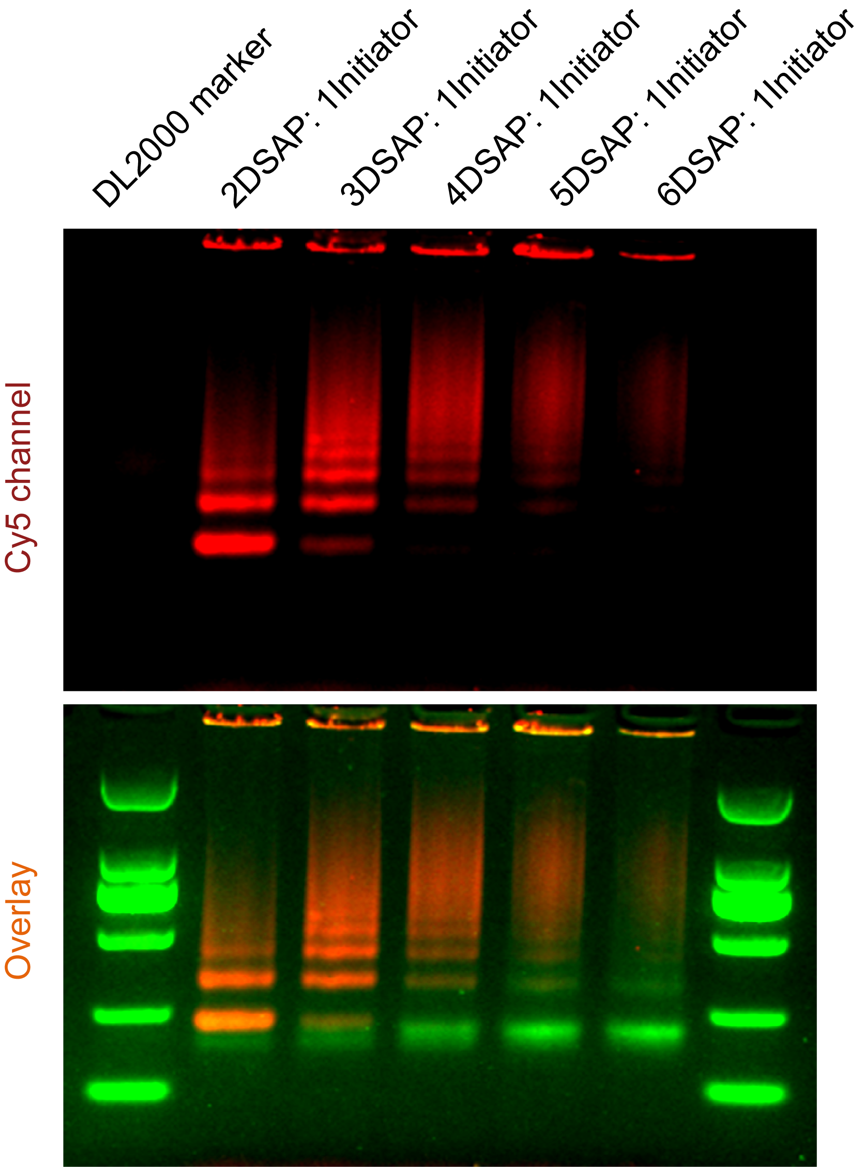
**Supplementary Fig. S22. The AGE showing the cascade amplification capacity of DSAP**.The AGE image displayed the signal amplification reaction between DSAP and the initiator. 500 nM initiator reacted with DSAP of increasing concentrations. The molar ratios between DSAP and initiator change from 1:1 to 6:1 (2-6 lanes) as the concentration of initiator decreases. The red bands and smear represent the HCR polymers due to Cy5 release.


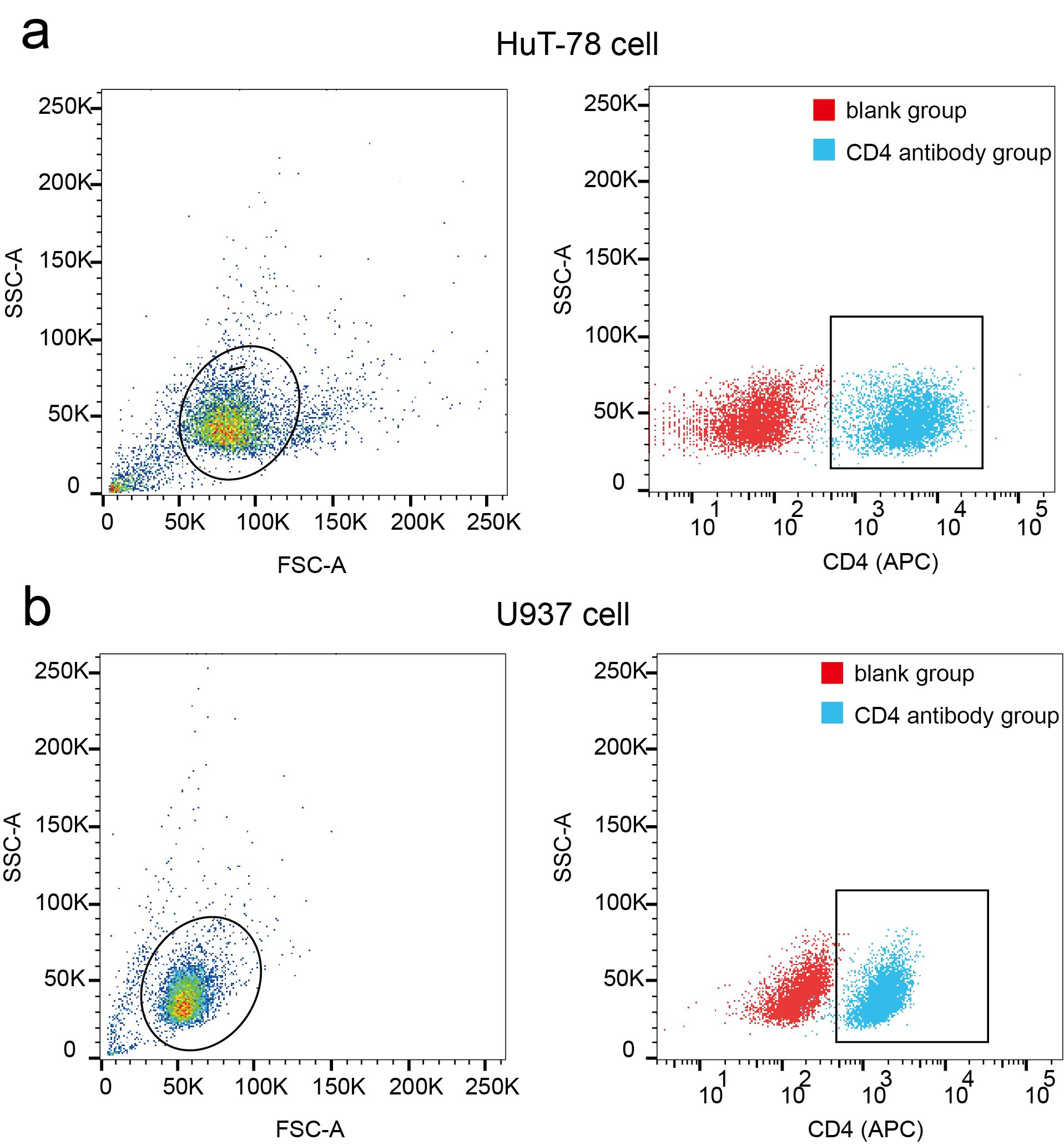
**Supplementary Fig. S23. The validation of CD4 expression in HuT-78 cells and U937 cells**. The FCA is used to demonstrate the CD4 expression of HuT-78 cells (a) and U937 cells (b). The dot figures on the right sides are overlaid by the blank group (blue) and the CD4 antibody group (red). The cells in the square gate indicated the cells were labeled with APC fluorescence.


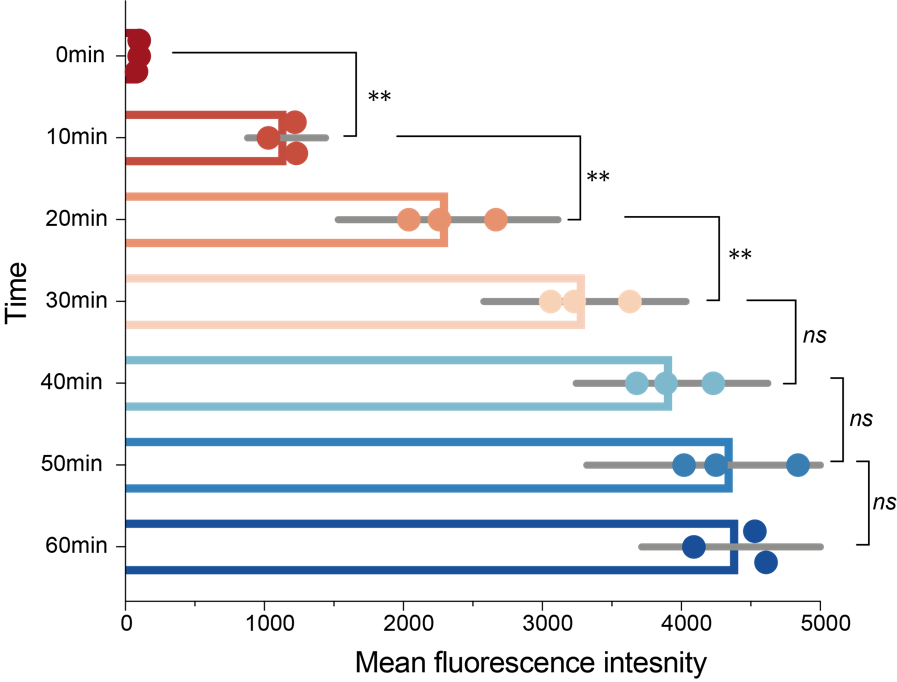
**Supplementary Fig. S24. The signal changes of HuT-78 cells treated with DSAP along with time.** The statistical analysis of mean fluorescence intensity decided by FCA of HuT-78 cells, which were treated with DSAP from 10 min to 60 min. The ** represented *p* < 0.01, ns represented no statistical significance.


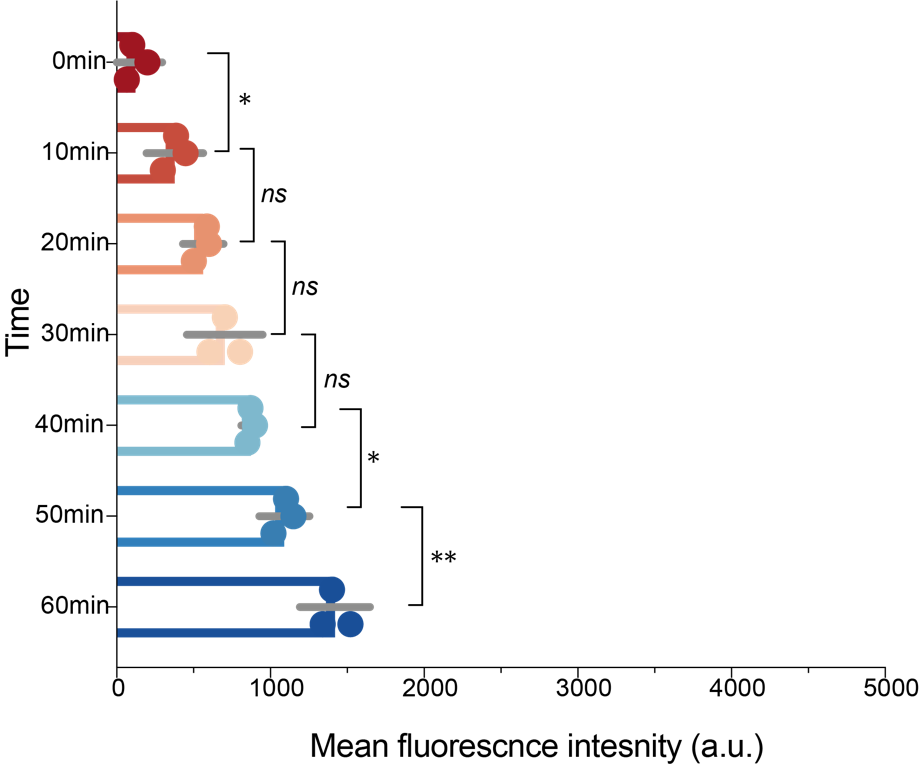
**Supplementary Fig. S25**. **The signal changes of HuT-78 cells treated with nude HCT probes along with time.** The statistical analysis of mean fluorescence intensity decided by FCA after HuT-78 cells were treated with DSAP from 10 min to 60 min. The ** represented *p* < 0.01, the * defined *p* < 0.05, and *ns* expressed no statistical significance.


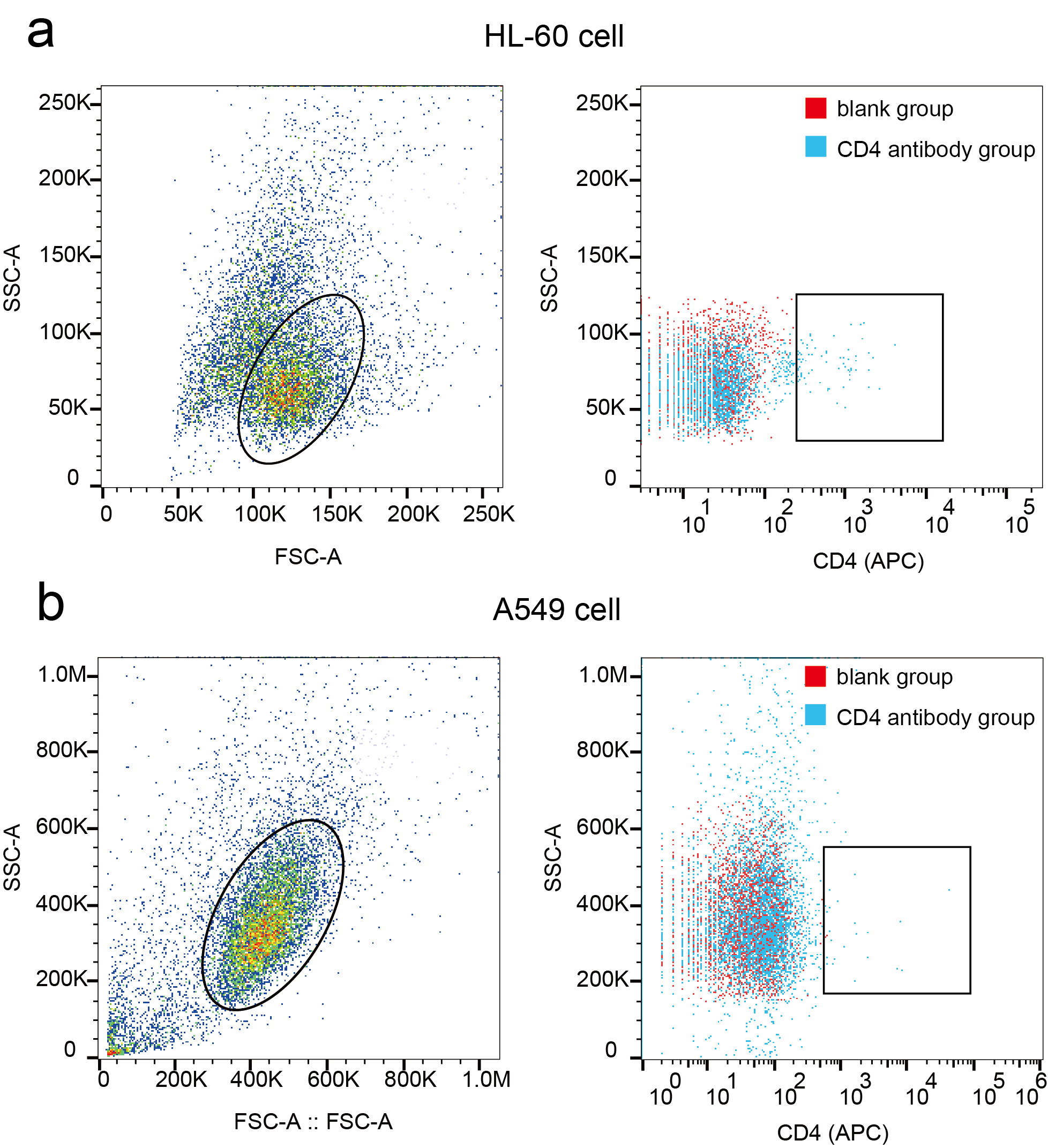
**Supplementary Fig. S26. The validation of CD4 expression in HL-60 cells and A549 cells.** The FCA demonstrates the CD4 expression of HL-60 cells (a) and A549 cells (b). The dot figures on the right sides are overlaid by the blank group (blue) and the CD4 antibody group (red). There are rare cells in the square gate, indicating the extremely low CD4 expression in the cells.


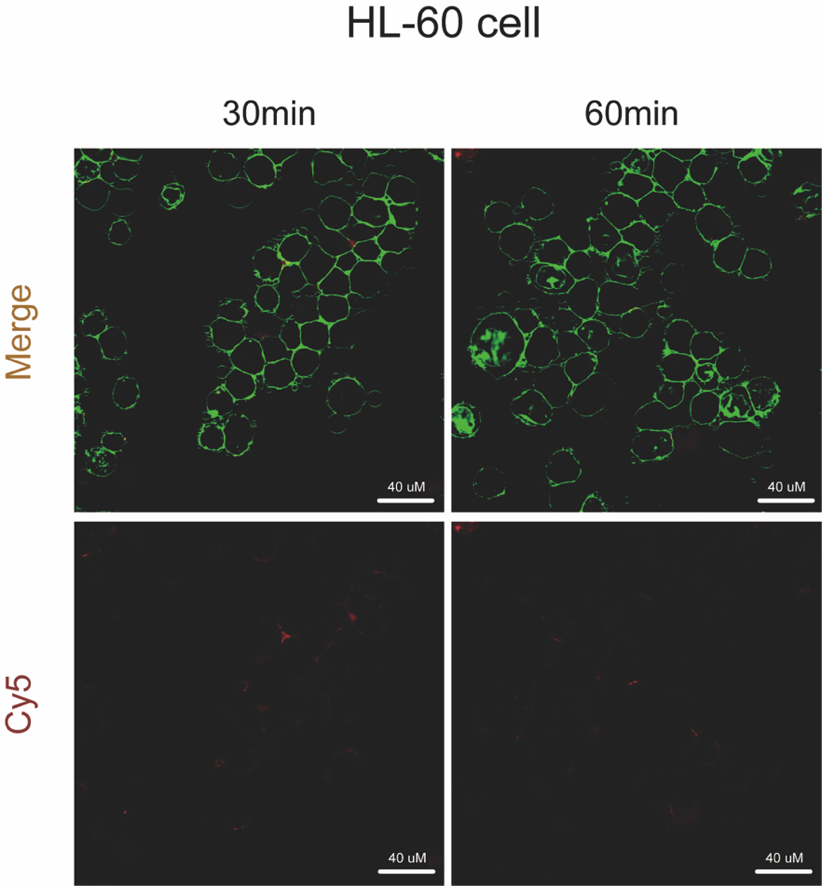
**Supplementary Fig. S27. The confocal laser scanning microscopy (CLSM) of HL-60 cells treated with DSAP for 30 min and 60 min, respectively.** The cell membranes were labeled by DiO dye (green channel). Scale bar, 40 μm


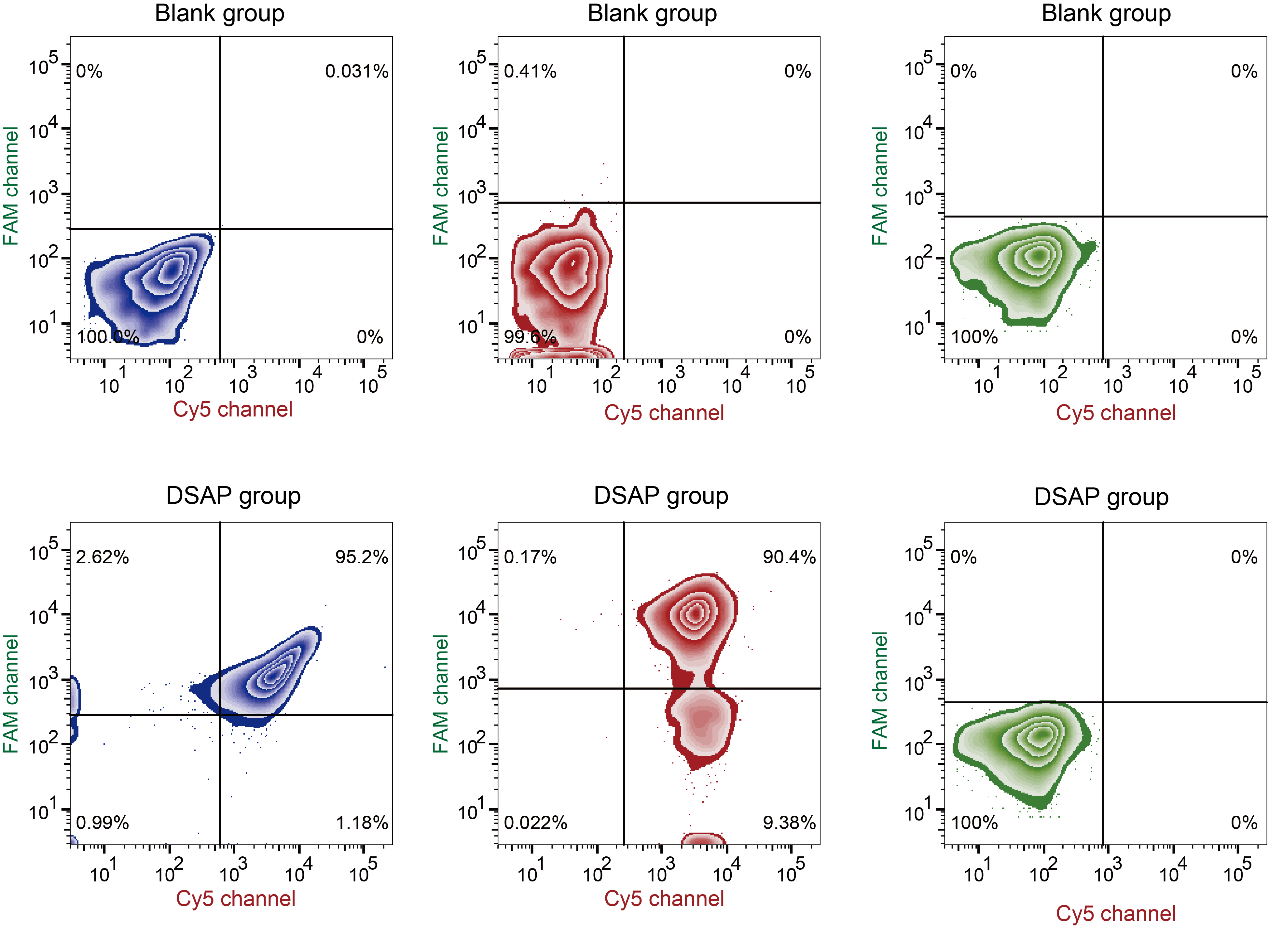
**Supplementary Fig. S28. The colocalization analysis of the Cy5 and Alexa 488 signal in U937 cells, CD4+ T lymphocytes, and HL-60 cells treated with FAM/Cy5/BHQ-2 labeled DSAP.** The blank group and DSAP treatment group of the three cells are shown in the figure.

**
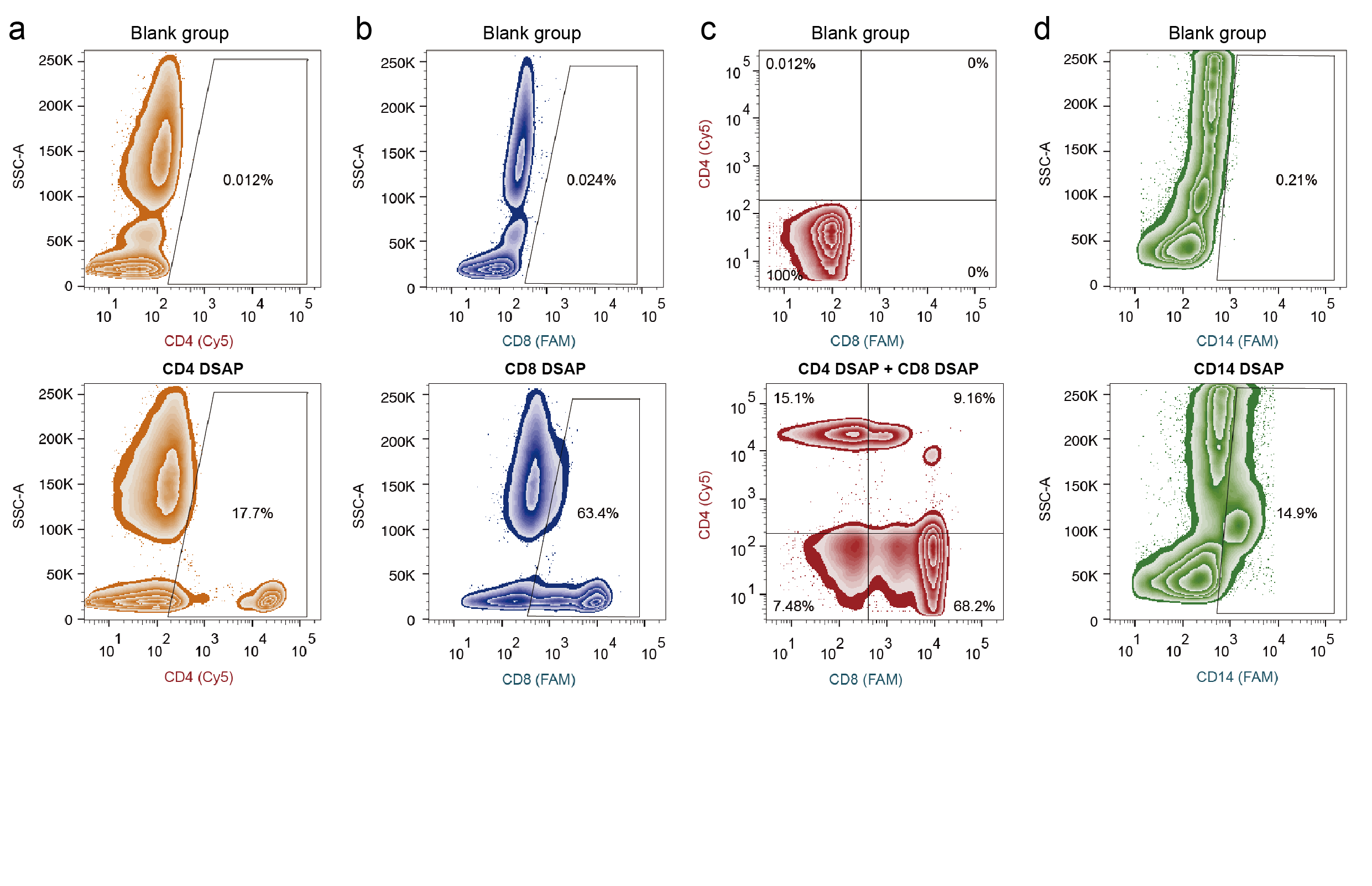
Supplementary Fig. S29. The validation of CD4+ T cell, CD8+ T cell, and monocytes detection by DSAP in whole blood samples.** The whole blood cells were treated with Cy5/BHQ-2 labeled CD4 DSAP, FAM/BHQ-1 labeled CD8 DSAP, and FAM/BHQ-1 labeled CD14 DSAP. The flow cytometry results of blank and DSAP-treated groups are shown. a) The FCA of whole blood cells treated with CD4 DSAP. b) The FCA of whole blood cells treated with CD8 DSAP. c) FCA of whole blood cells treated with CD4 DSAP and CD8 DSAP. The Cy5 and FAM signal analysis of lymphocytes is shown. d) FCA of whole blood cells treated with CD14 DSAP.


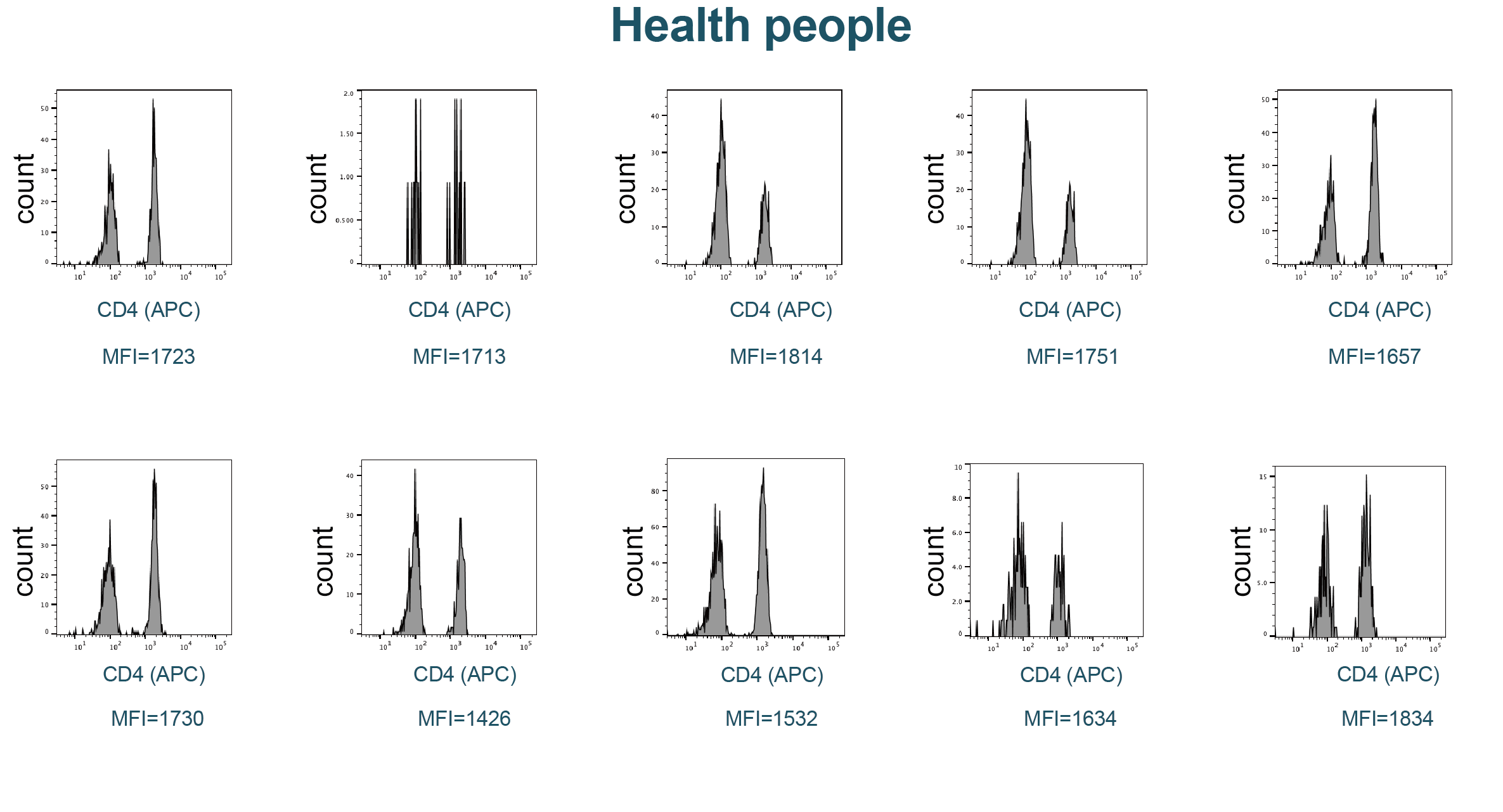


**Supplementary Fig. S30. The CD4+ T lymphocyte detection of ten blood samples from healthy people by flow cytometry.** The whole blood cells were stained with APC-labeled CD4 antibody and were detected by flow cytometry. The mean fluorescence intensity (MFI) of the CD4+ T lymphocytes in the APC channel is shown under the histograms.


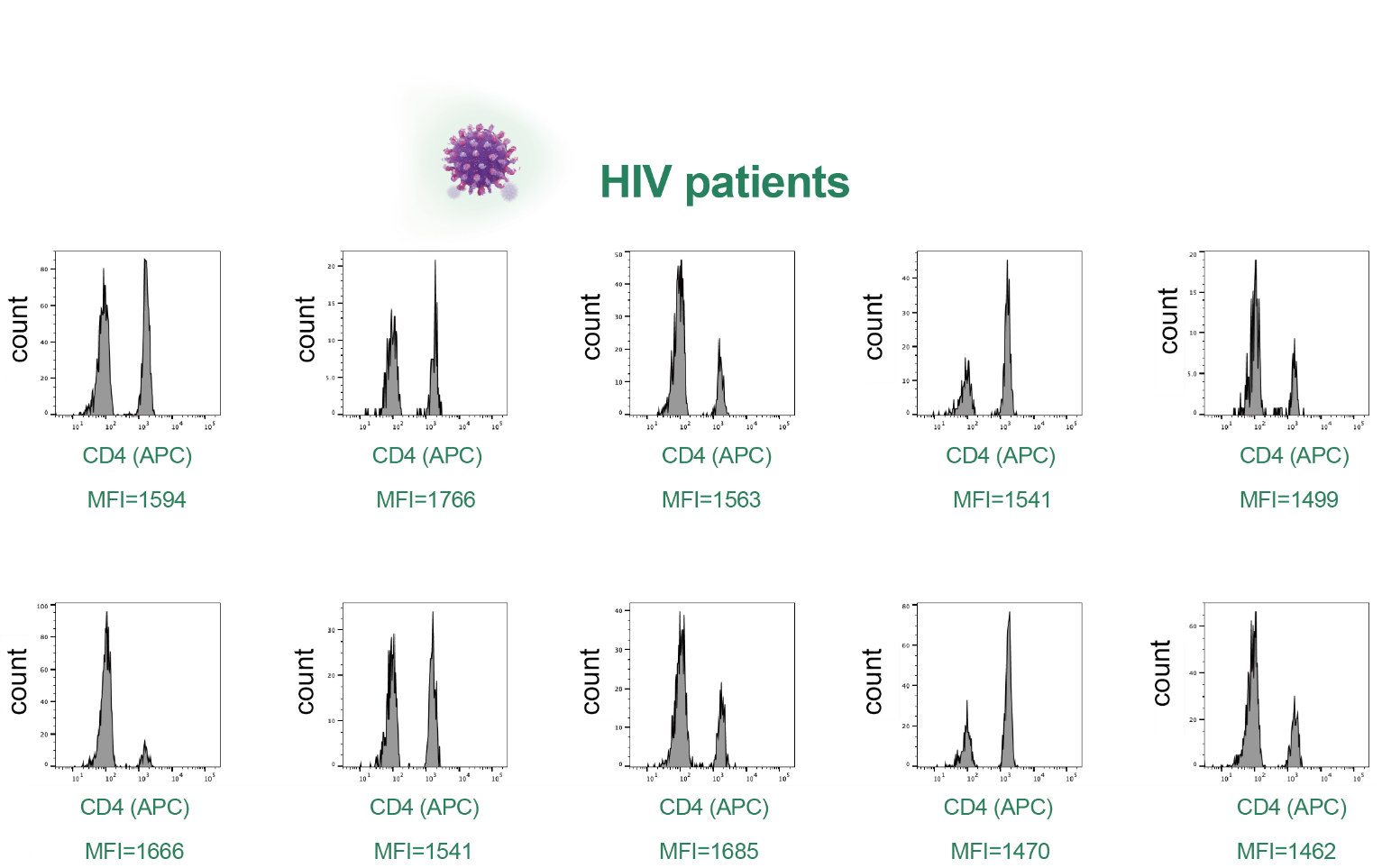


**Supplementary Fig. S31. The CD4+ T lymphocyte detection of ten blood samples from HIV patients by flow cytometry.** The whole blood cells were stained with APC-labeled CD4 antibody and were detected by flow cytometry.


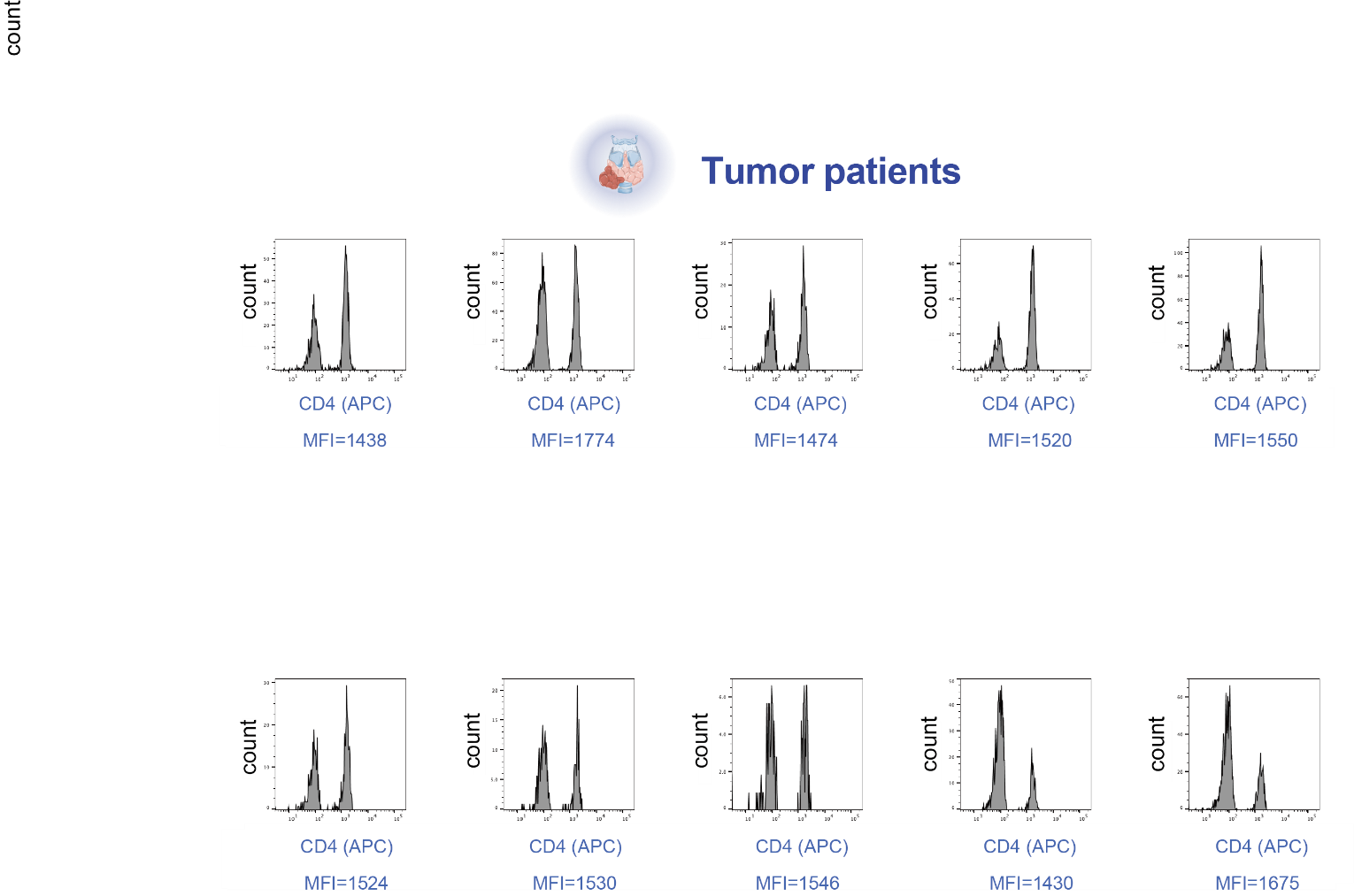


**Supplementary Fig. S32. The CD4+ T lymphocyte detection of ten blood samples from tumor patients by flow cytometry.** The whole blood cells were stained with APC-labeled CD4 antibody and were detected by flow cytometry.


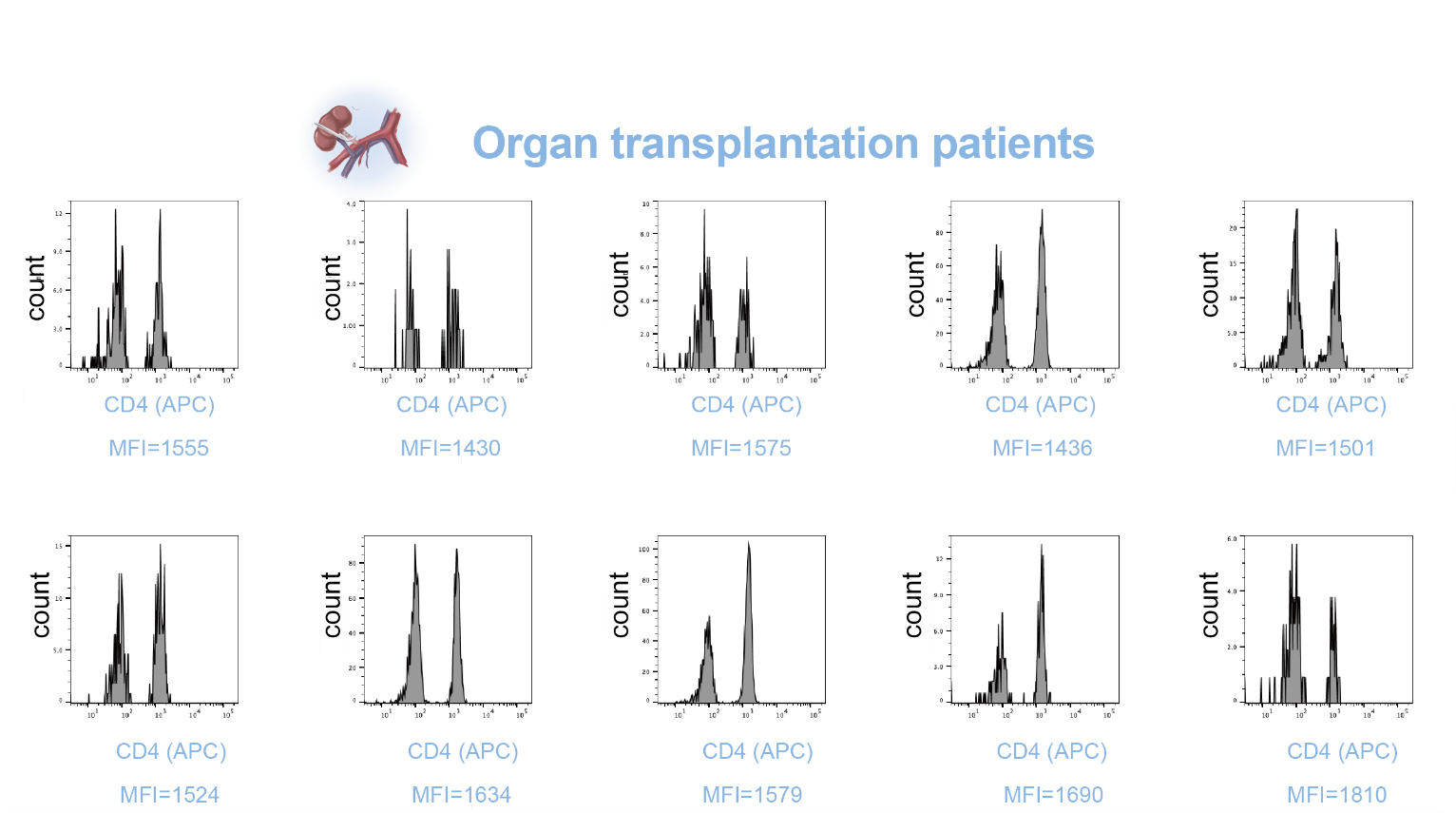


**Supplementary Fig. S33. The CD4+ T lymphocyte detection of ten blood samples from organ transplantation patients by flow cytometry.** The whole blood cells were stained with APC-labeled CD4 antibody and were detected by flow cytometry.


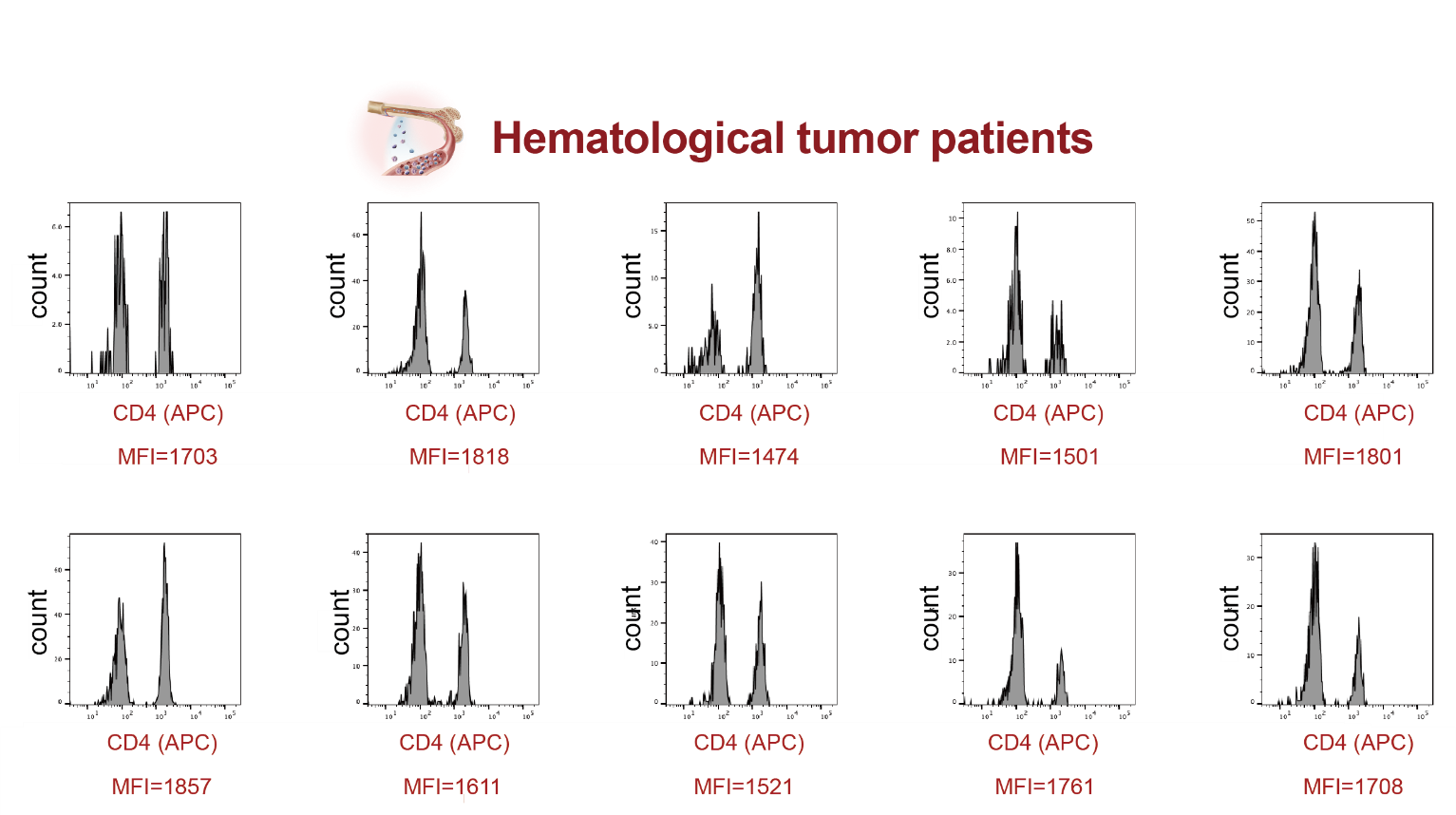


**Supplementary Fig. S34. The CD4+ T lymphocyte detection of ten blood samples from hematological tumor patients by flow cytometry.** The whole blood cells were stained with APC-labeled CD4 antibody and were detected by flow cytometry.


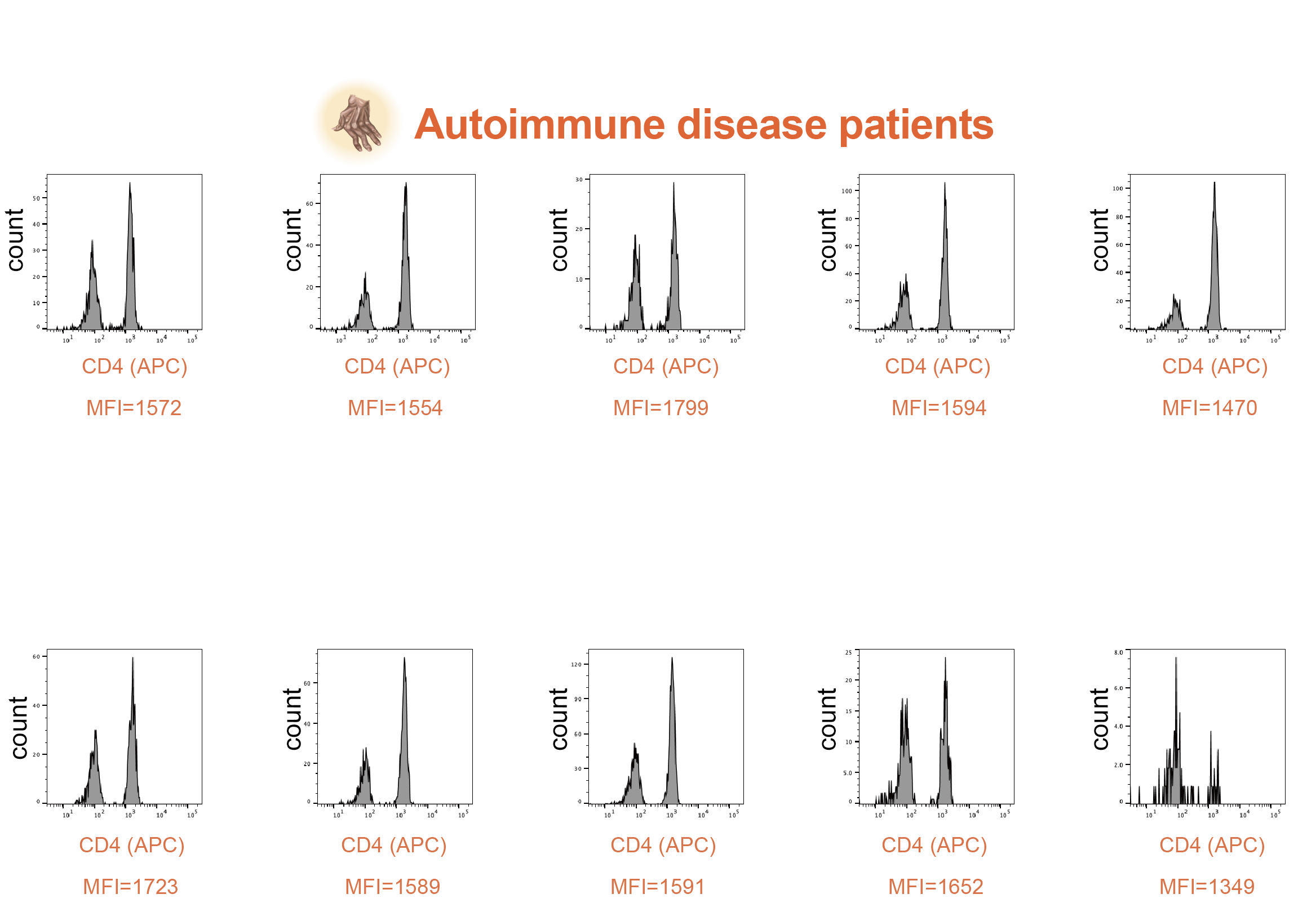


**Supplementary Fig. S35. The CD4+ T lymphocyte detection of ten blood samples from autoimmune disease patients by flow cytometry.** The whole blood cells were stained with APC-labeled CD4 antibody and were detected by flow cytometry.

**Supplementary Fig. S36. The analysis of CD4 expression level in CD4+ T lymphocytes from the patients with different health situations and diseases.** The statistical analysis of mean fluorescence intensity of CD4+ T lymphocytes decided by FCA for the blood samples from healthy people and the patients with HIV, tumor, organ transplantation, hematological tumor, and autoimmune diseases. The SD values were decided by ten different healthy people or patients with the same diseases. ns presented no statistical significance.

**Supplementary Fig. S37. The stability of the detection results based on DSAP.** The CD4+ T lymphocyte concentration of immuo-trol cells and immuno-trol low cells is confirmed as 563/μL and 113/μL by flow cytometry. The blood cell number in immuno-trol cells remained unchanged during the expiration date. The immuno-trol cells and immuno-trol low cells were treated with DSAP. We calculated the CD4+ T lymphocyte concentration according to the linear equation. We carried out the three parallel measurements every day for 7 days. The dots in the figure represent every measurement result.


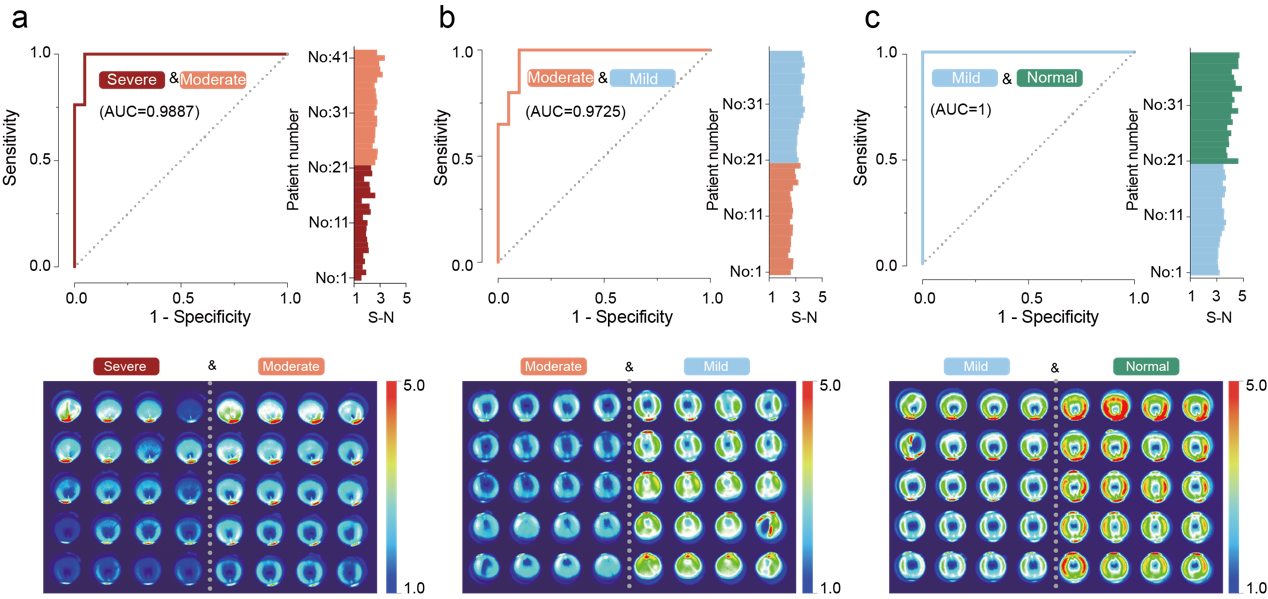
**Supplementary Fig. S38. The application of DSAP-based immune monitoring in HIV immunodeficiency staging.** The receiver operating characteristic curve and area under the curve are shown. The signal-to-noise of DSAP in detecting the blood samples from HIV patients is displayed alongside the ROC curve. The visualization of the detection results is shown under the ROC curve. The microplates, after detection, were scanned with the iBright scanning system. The thermal figures reflect the fluorescence intensity of the microwells. The diagnosis capacity of DSAP in judging severe/moderate immunodeficiency (a), moderate/mild immunodeficiency (b), and mild/normal immunodeficiency (c) are shown in the figure.

**supplementary Table 1. The sequences (5’-3’) of DSAP and aptamers in this work.**

| **Strand** | Sequences |
| --- | --- |
| **S1-SE**  **(13nt)** | ATTTATCACCCGCCATAGTAGACGTATCACCAGGCAGTTGAGACGAACATTCCTAAGTCTGAA tt tgtggtggtgagt |
| **S2-SE**  **(13nt)** | ACATGCGAGGGTCCAATACCGACGATTACAGCTTGCTACACGATTCAGACTTAGGAATGTTCG tt gtgtggtgtgaag |
| **S1-SE (17nt)** | ATTTATCACCCGCCATAGTAGACGTATCACCAGGCAGTTGAGACGAACATTCCTAAGTCTGAA tt atattgtggtggtgagt |
| **S2-SE (17nt)** | ACATGCGAGGGTCCAATACCGACGATTACAGCTTGCTACACGATTCAGACTTAGGAATGTTCG tt tggagtgtggtgtgaag |
| **S1-SE (21nt)** | ATTTATCACCCGCCATAGTAGACGTATCACCAGGCAGTTGAGACGAACATTCCTAAGTCTGAA tt tataatattgtggtggtgagt |
| **S2-SE (21nt)** | ACATGCGAGGGTCCAATACCGACGATTACAGCTTGCTACACGATTCAGACTTAGGAATGTTCG tt tagatggagtgtggtgtgaag |
| **S3** | ACTACTATGGCGGGTGATAAAACGTGTAGCAAGCTGTAATCGACGGGAAGAGCATGCCCATCC |
| **S4** | ACGGTATTGGACCCTCGCATGACTCAACTGCCTGGTGATACGAGGATGGGCATGCTCTTCCCG |
| **CD4 original aptamer** | GCCTGTTGTGAGCCTCCTAACGATGTCGACGTGCAGCTTCCTTGAGCCTTACTGAAAATACTACCCAGTCCATGCTTATTCTTGTCTCCC |
| **CD4T1** | GCCTGTTGTGAGCCTCCTAACGATGTCGACGTGCAGCTTCCTTGAGCCTTACTGAAAATACTACCCAGTC |
| **CD4T2** | GATGTCGACGTGCAGCTTCCTTGAGCCTTACTGAAAATACTACCCAGTC |
| **CD4 truncated aptamer** | CAGCTTCCTTGAGCCTTACTGAAAATACTACCCAGTC |
| **CD8 original aptamer** | CCAGAGTGACGCAGCAACAGAGGTGTAGAAGTACACGTGAACAAGCTTGAAATTGTCTCTGACAGAGGTGGACACGGTGGCTTAGT |
| **CD8T1** | ACAGAGGTGTAGAAGTACACGTGAACAAGCTTGAAATTGTCTCTGACAGAGG |
| **CD8T2** | ACAGAGGTGTAGAAGTACACGTGAACAAGCTTGAAATTGTCTCTGA |
| **CD8 truncated aptamer** | AGAGGTGTAGAAGTACACGTGAACAAGCTTGAAATTGTCTCT |
| **CD14 original aptamer** | ATCCAGAGTGACGCAGCAGAAGAGTAGATGAAACGTTTTTTCGCCCGATAAAAGGGACGTGCGTCAGACATGGACACGGTGGCTTAGT |
| **CD14 truncated aptamer** | ACGCAGCAGAAGAGTAGATGAAACGTTTTTTCGCCCGATAAAAGGGACGTGCGT |
| **Hairpin1-13 nt SE (CD4)** | CAGCTTCCTTGAGCCTTACTGAAAA/iCy5dT/ACTACCCAGTCATATATAGGGTAG/iBHQ2dT/ATTTTCAGTA tt actcaccaccaca |
| **Hairpin2-13 nt SE (CD4)** | GGGTAGTATTTTCAGTCAGGCTCAAGGTACTGAAAATACTACCATATATATGACT tt cttcacaccacac |
| **Hairpin1-17 nt SE (CD4)** | CAGCTTCCTTGAGCCTTACTGAAAA/iCy5dT/ACTACCCAGTCATATATAGGGTAG/iBHQ2dT/ATTTTCAGTA tt actcaccaccacaatat |
| **Hairpin2-17 nt SE (CD4)** | GGGTAGTATTTTCAGTCAGGCTCAAGGTACTGAAAATACTACCATATATATGACT tt cttcacaccacactcca |
| **Hairpin1-21 nt SE (CD4)** | CAGCTTCCTTGAGCCTTACTGAAAA/iCy5dT/ACTACCCAGTCATATATAGGGTAG/iBHQ2dT/ATTTTCAGTA tt actcaccaccacaatattata |
| **Hairpin2-21 nt SE (CD4)** | GGGTAGTATTTTCAGTCAGGCTCAAGGTACTGAAAATACTACCATATATATGACT tt cttcacaccacactccatcta |
| **Hairpin1 (CD8)** | AGAGGTGTAGAAGTACACG/iFAMdT/GAACAAGCTTGAAATTGTCTCTATTCATACAATTTCAAGCTTGT/iDabcyldT/CACGT tt actcaccaccaca |
| **Hairpin2 (CD8)** | CAATTTCAAGCTTGTTCACGCGTACTTCTACAACGTGAACAAGCTTGAAATTA TATGAATAGAGA tt cttcacaccacac |
| **Hairpin1 (CD14)** | ACGCAGCAGAAGAGTAGATGAAACGTTTTTTCGCCCGA/iFAMdT/AAAAGGGACGTGCGTATTACATATTTA/iDabcyldT/CGGGCG tt attcaccaccaca |
| **Hairpin2 (CD14)** | TTTATCGGGCGAAAAAACGTTTCCGCCCGATAAATATGTAATACGCA tt cttcacaccacac |

**supplementary Table 2. The results of clinical flow cytometry and DSAP in blood samples from the patients with HIV, tumor, organ transplantation, autoimmune diseases and organ transplantation**. At least three times parallel experiments were conducted in the DSAP group.

| **Clinical results** | **DSAP results (signal-to-noise, a.u.)** | | |
| --- | --- | --- | --- |
| HIV patients | | | |
| 15 | 1.6748 | 1.6682 | 1.6381 |
| 18 | 1.8639 | 1.893 | 1.7553 |
| 30 | 1.9653 | 2.0419 | 2.3731 |
| 93 | 1.894 | 1.9707 | 2.0387 |
| 100 | 2.1481 | 2.3661 | 2.354 |
| 150 | 1.9854 | 1.5435 | 1.789 |
| 151 | 2.2903 | 2.4648 | 2.344 |
| 158 | 2.3242 | 2.3909 | 2.5031 |
| 189 | 2.5841 | 2.5609 | 2.5342 |
| 194 | 2.4975 | 2.4113 | 2.4448 |
| 199 | 2.435 | 1.543 | 2.134 |
| 208 | 2.7236 | 2.8468 | 2.442 |
| 214 | 2.89 | 2.5906 | 2.983 |
| 228 | 2.7153 | 2.9568 | 2.6711 |
| 234 | 2.724 | 2.63 | 2.8721 |
| 237 | 2.756 | 2.938 | 2.698 |
| 241 | 2.7193 | 2.7065 | 2.7685 |
| 253 | 2.6478 | 2.6319 | 2.7491 |
| 284 | 2.3318 | 2.6018 | 2.8378 |
| 299 | 2.6164 | 2.846 | 2.642 |
| 308 | 3.0979 | 2.9333 | 3.0253 |
| 309 | 2.8969 | 2.9904 | 2.9377 |
| 332 | 2.6392 | 2.561 | 2.47 |
| 344 | 2.79834 | 2.983 | 2.534 |
| 370 | 3.0111 | 3.3529 | 3.2605 |
| 421 | 3.8 | 3.32 | 3.98 |
| 444 | 3.3737 | 3.4818 | 3.2932 |
| 456 | 3.4324 | 3.4661 | 3.4661 |
| 485 | 3.8015 | 3.7896 | 3.3832 |
| 528 | 3.7887 | 3.9547 | 4.0253 |
| 544 | 4.4545 | 4.2873 | 3.6545 |
| 568 | 4.2637 | 3.6553 | 4.5591 |
| 575 | 4.0133 | 3.8867 | 3.8873 |
| 602 | 4.6904 | 5.0801 | 4.9023 |
| 616 | 4.5126 | 4.4554 | 4.311 |
| 702 | 4.6754 | 4.6143 | 4.7941 |
| 710 | 4.8333 | 4.7933 | 5.0749 |
| 895 | 5.3373 | 5.5822 | 5.0969 |
| 968 | 5.3832 | 6.01291 | 6.32 |
| 745 | 4.98 | 4.5 | 4.3 |
| 523 | 3.56 | 3.612 | 3.98 |
| 99 | 1.64 | 1.543 | 1.873 |
| 534 | 3.78956 | 3.987 | 3.5839 |
| 103 | 1.456 | 1.983 | 1.345 |
| 230 | 2.23 | 2.983 | 2.5 |
| Tumor patients | | | |
| 126 | 2.2409 | 2.1617 | 2.36 |
| 225 | 2.6648 | 2.52 | 2.754 |
| 225 | 2.6313 | 2.9793 | 2.6927 |
| 317 | 3.072 | 3.3022 | 3.3023 |
| 395 | 3.3115 | 3.2271 | 3.5987 |
| 395 | 3.5434 | 3.6445 | 3.5422 |
| 424 | 3.5617 | 3.44 | 3.82 |
| 430 | 3.4212 | 3.508 | 3.768 |
| 453 | 3.7769 | 3.6036 | 3.6674 |
| 481 | 3.3349 | 3.7977 | 3.5167 |
| 591 | 4.7125 | 4.5339 | 4.5422 |
| 605 | 4.3366 | 4.6632 | 5.2944 |
| 622 | 4.5319 | 4.2074 | 4.1768 |
| 1241 | 6.5426 | 7.6618 | 7.1798 |
| 1330 | 6.1248 | 6.5625 | 6.7509 |
| 1685 | 7.6619 | 7.9865 | 7.6777 |
| 660 | 4.687 | 4.789 | 4.432 |
| 355 | 3.2113 | 3.4324 | 3.012 |
| 4 | 1.8243 | 1.321 | 1.556 |
| 1127 | 6.898 | 6.546 | 7.212 |
| 1185 | 6.909 | 7.012 | 6.654 |
| 464 | 3.8789 | 3.32 | 3.856 |
| 510 | 4.325 | 4.986 | 4.578 |
| Organ transplantation patients | | | |
| 74 | 2.0183 | 2.0142 | 1.9858 |
| 215 | 2.4264 | 2.446 | 2.426 |
| 382 | 3.2354 | 3.2585 | 3.2546 |
| 431 | 3.4123 | 3.492 | 3.2864 |
| 530 | 3.9569 | 3.938 | 3.789 |
| 784 | 5.1183 | 5.1908 | 5.2882 |
| 787 | 4.6402 | 4.6222 | 4.7329 |
| 865 | 4.7497 | 5.0078 | 5.3844 |
| 908 | 5.7741 | 5.509 | 5.7599 |
| 1074 | 6.2376 | 6.278 | 6.2376 |
| 1344 | 6.896 | 7.08 | 6.5436 |
| 1080 | 6.543 | 6.89 | 6.2 |
| 1314 | 6.98 | 7.532 | 7.43 |
| 217 | 2.87 | 2.234 | 2.654 |
| 1335 | 7.32 | 6.897 | 7.12 |
| 688 | 4.89 | 4.902 | 4.58 |
| 1059 | 6.983 | 6.543 | 6.4987 |
| Autoimmune disease patients | | | |
| 81 | 1.9112 | 1.82 | 2.04 |
| 170 | 2.2743 | 2.42 | 2.24 |
| 204 | 2.585 | 2.6335 | 2.6689 |
| 223 | 2.7213 | 2.56 | 2.64 |
| 288 | 2.7133 | 2.7222 | 2.5459 |
| 356 | 3.1408 | 3.3784 | 2.8 |
| 357 | 3.0609 | 2.886 | 3.26 |
| 451 | 3.794 | 3.596 | 3.42 |
| 478 | 3.2924 | 3.5796 | 3.5792 |
| 506 | 3.3856 | 3.3596 | 3.7096 |
| 596 | 4.3807 | 4.5467 | 4.5353 |
| 634 | 4.1554 | 3.935 | 4.2452 |
| 743 | 4.4987 | 4.6105 | 4.9043 |
| 815 | 5.0648 | 5.24 | 5.04 |
| 978 | 6.014 | 6.2599 | 5.9957 |
| 1012 | 5.8482 | 5.9476 | 6.0756 |
| 1224 | 6.5892 | 7.6862 | 6.216 |
| 550 | 4.876 | 4.43 | 4.546 |
| Hematological tumor patients | | | |
| 14 | 1.9267 | 1.8067 | 1.8662 |
| 21 | 1.6401 | 1.64 | 1.86 |
| 72 | 2.1727 | 1.9839 | 1.3669 |
| 102 | 2.0085 | 2.3496 | 2.24 |
| 104 | 2.5485 | 2.7137 |  |
| 142 | 2.3766 | 2.1018 | 2.2662 |
| 187 | 2.62 | 2.7733 | 2.6129 |
| 195 | 2.7725 | 2.6289 | 2.4463 |
| 294 | 2.7057 | 2.7132 | 2.9406 |
| 366 | 3.2164 | 3.0228 | 3.3388 |
| 474 | 3.5644 | 4.0637 | 3.2712 |
| 589 | 4.4142 | 4.2134 | 4.3669 |
| 687 | 4.7024 | 4.6588 | 4.7803 |
| 1184 | 6.6926 | 6.8435 | 6.16 |
| 882 | 5.2167 | 5.4287 | 5.292 |
| 1104 | 6.0733 | 6.58 | 6.83 |
| 981 | 5.9944 | 5.851 | 6.027 |

**supplementary Table 3. The consistency comparison between flow cytometry results and DSAP results.** The calculation results of DSAP-based were obtained by "y = 0.004164 x + 1.706"

| **Flow cytometry results** | **DSAP results** | | | |
| --- | --- | --- | --- | --- |
|  | Signal-to-noise (a.u.) | | | Calculated results |
| 73 | 2.0233 | 1.8858 | 2.3305 | 90 |
| 98 | 1.9845 | 2.0139 | 2.1111 | 79 |
| 141 | 2.2397 | 2.2553 | 2.0956 | 118 |
| 210 | 2.7313 | 2.9767 | 2.6787 | 262 |
| 305 | 2.938 | 3.3033 | 3.3576 | 359 |
| 325 | 3.3807 | 3.3193 | 3.406 | 399 |
| 367 | 3.1626 | 3.1212 | 3.0821 | 340 |
| 412 | 3.4407 | 3.8372 | 3.4488 | 449 |
| 517 | 3.7886 | 3.7275 | 3.8803 | 503 |
| 531 | 3.7552 | 4.3586 | 4.1586 | 573 |
| 535 | 3.8307 | 4.508 | 4.2607 | 599 |
| 558 | 4.4612 | 3.7613 | 4.239 | 588 |
| 567 | 4.2324 | 4.3401 | 4.4263 | 631 |
| 739 | 4.8978 | 4.991 | 5.0476 | 786 |
| 763 | 4.7623 | 4.7564 | 4.664 | 726 |
| 825 | 4.8728 | 5.2334 | 5.0736 | 824 |
| 837 | 5.2672 | 5.3593 | 5.3103 | 867 |
| 983 | 5.9944 | 5.851 | 6.027 | 1021 |

**supplementary Table 4. The mean signal-to-noise by DSAP when detecting blood samples with different CD4+ T lymphocyte concentration intervals**.

| **<100** | **100~200** | **200~300** | **300~400** | **400~500** | **500~600** | **600~700** | **700~800** | **800~900** | **900~1000** | **1000~1200** | **>1200** |
| --- | --- | --- | --- | --- | --- | --- | --- | --- | --- | --- | --- |
| 1.5671 | 2.2894 | 2.629133 | 3.199633 | 3.575567 | 3.497525 | 4.890933 | 4.6946 | 5.114933 | 5.681 | 5.957133 | 6.714575 |
| 1.93574 | 2.199367 | 2.795567 | 3.018833 | 3.7 | 4.629667 | 4.426333 | 4.9788 | 5.136975 | 5.90537 | 6.6749 | 7.128067 |
| 1.660367 | 1.594667 | 2.8212 | 2.941667 | 3.53324 | 3.7988 | 4.305367 | 4.70055 | 5.312267 | 6.099225 | 6.251067 | 7.314 |
| 1.8374 | 2.6311 | 2.4328 | 3.368667 | 3.565733 | 3.717333 | 4.1263 | 4.593333 | 5.180275 | 5.957467 | 6.544333 | 6.4794 |
| 1.713367 | 2.2542 | 2.586 | 2.77178 | 3.3969 | 3.9229 | 4.636 | 4.727567 | 5.312467 | 5.957467 | 6.885333 | 7.112333 |
| 2.126767 | 2.196867 | 2.640433 | 3.218567 | 3.3919 | 3.894633 | 4.628667 | 5.1991 | 5.312467 | 5.78475 | 6.858333 | 6.839867 |
| 2.079867 | 1.772633 | 2.646267 | 3.1064 | 3.603333 | 4.0908 | 4.713833 | 4.6353 | 5.347825 |  |  | 7.775367 |
| 2.0061 | 2.406067 | 2.767767 | 3.068967 | 3.682633 | 3.78682 | 4.790667 | 4.7554 |  |  |  |  |
| 1.923733 | 2.311433 | 2.781067 | 3.1196 | 3.454867 | 4.1998 |  | 4.8133 |  |  |  |  |
| 1.9678 | 2.559733 | 2.571 | 3.121967 | 3.684967 | 4.0709 |  |  |  |  |  |  |
| 2.0365 | 2.4512 | 2.742033 | 3.208167 | 3.609525 | 4.617333 |  |  |  |  |  |  |
| 1.685333 | 2.6159 | 2.797333 | 3.2495 | 3.5023 | 4.153833 |  |  |  |  |  |  |
|  | 2.037333 | 2.731433 | 3.3791 |  | 4.332933 |  |  |  |  |  |  |
|  |  | 2.676267 |  |  | 4.159367 |  |  |  |  |  |  |
|  |  | 2.590467 |  |  |  |  |  |  |  |  |  |
|  |  | 2.7865 |  |  |  |  |  |  |  |  |  |

**supplementary Table 5. The mean signal-to-noise of blood samples from HIV patients of severe, moderate, mild, and normal periods by DSAP.**

| **Severe** | **Moderate** | **Mild** | **Normal** |
| --- | --- | --- | --- |
| 1.5671 | 2.62913333 | 3.21856667 | 3.497525 |
| 1.93574 | 2.79556667 | 3.1064 | 4.62966667 |
| 1.66036667 | 2.8212 | 3.06896667 | 3.7988 |
| 1.8374 | 2.4328 | 3.1196 | 3.71733333 |
| 1.71336667 | 2.586 | 3.12196667 | 3.9229 |
| 2.12676667 | 2.64043333 | 3.20816667 | 3.89463333 |
| 2.07986667 | 2.64626667 | 3.2495 | 4.0908 |
| 2.0061 | 2.76776667 | 3.3791 | 3.78682 |
| 1.92373333 | 2.78106667 | 3.57556667 | 4.1998 |
| 1.9678 | 2.571 | 3.7 | 4.0709 |
| 2.0365 | 2.74203333 | 3.53324 | 4.61733333 |
| 1.68533333 | 2.79733333 | 3.56573333 | 4.15383333 |
| 2.2894 | 2.73143333 | 3.3969 | 4.33293333 |
| 2.19936667 | 2.67626667 | 3.3919 | 4.15936667 |
| 1.59466667 | 2.59046667 | 3.60333333 | 4.89093333 |
| 2.6311 | 2.7865 | 3.68263333 | 4.42633333 |
| 2.2542 | 3.19963333 | 3.45486667 | 4.30536667 |
| 2.19686667 | 3.01883333 | 3.68496667 | 4.1263 |
| 1.77263333 | 2.94166667 | 3.609525 | 4.636 |
| 2.40606667 | 2.77178 | 3.5023 | 4.62866667 |
| 2.31143333 |  |  | 4.71383333 |
| 2.55973333 |  |  | 4.79066667 |
| 2.4512 |  |  | 4.6946 |
| 2.6159 |  |  | 4.9788 |
| 2.03733333 |  |  | 4.70055 |
|  |  |  | 4.59333333 |
|  |  |  | 4.72756667 |
|  |  |  | 5.1991 |
|  |  |  | 4.6353 |
|  |  |  | 5.11493333 |
|  |  |  | 5.136975 |
|  |  |  | 5.31226667 |
|  |  |  | 5.180275 |
|  |  |  | 5.31246667 |
|  |  |  | 5.31246667 |
|  |  |  | 5.347825 |
|  |  |  | 5.681 |
|  |  |  | 5.90537 |
|  |  |  | 6.099225 |
|  |  |  | 5.95746667 |
|  |  |  | 5.95746667 |
|  |  |  | 5.95713333 |

**supplementary Table 6. The mean signal-to-noise of blood samples from HIV patients of mild period (350~500/μL CD4+ T lymphocytes) and normal period (>500/μL CD4+ T lymphocytes)**

| **350~500** | **>500** |
| --- | --- |
| 3.21856667 | 4.62966667 |
| 3.1064 | 3.7988 |
| 3.06896667 | 3.71733333 |
| 3.1196 | 3.9229 |
| 3.12196667 | 3.89463333 |
| 3.20816667 | 4.0908 |
| 3.2495 | 3.78682 |
| 3.3791 | 4.1998 |
| 3.57556667 | 4.0709 |
| 3.7 | 4.61733333 |
| 3.53324 | 4.15383333 |
| 3.56573333 | 4.33293333 |
| 3.3969 | 4.15936667 |
| 3.3919 | 4.89093333 |
| 3.60333333 | 4.42633333 |
| 3.68263333 | 4.30536667 |
| 3.45486667 | 4.1263 |
| 3.68496667 | 4.636 |
| 3.609525 | 4.62866667 |
| 3.5023 | 4.71383333 |

**supplementary Table 7. The mean signal-to-noise of blood samples from HIV patients of moderate period (200~350/μL CD4+ T lymphocytes) and mild period (350~500/μL CD4+ T lymphocytes).**

| **200~350** | **350~500** |
| --- | --- |
| 2.62913333 | 3.21856667 |
| 2.79556667 | 3.1064 |
| 2.8212 | 3.06896667 |
| 2.4328 | 3.1196 |
| 2.586 | 3.12196667 |
| 2.64043333 | 3.20816667 |
| 2.64626667 | 3.2495 |
| 2.76776667 | 3.3791 |
| 2.78106667 | 3.57556667 |
| 2.571 | 3.7 |
| 2.74203333 | 3.53324 |
| 2.79733333 | 3.56573333 |
| 2.73143333 | 3.3969 |
| 2.67626667 | 3.3919 |
| 2.59046667 | 3.60333333 |
| 2.7865 | 3.68263333 |
| 3.19963333 | 3.45486667 |
| 3.01883333 | 3.68496667 |
| 2.94166667 | 3.609525 |
| 3.36866667 | 3.5023 |

**supplementary Table 8. The mean signal-to-noise of blood samples from HIV patients of severe period (0~200/μL CD4+ T lymphocytes) and moderate period (200~350/μL CD4+ T lymphocytes).**

| **0~200** | **200~350** |
| --- | --- |
| 1.5671 | 2.62913333 |
| 1.93574 | 2.79556667 |
| 1.66036667 | 2.8212 |
| 1.8374 | 2.4328 |
| 1.71336667 | 2.586 |
| 2.12676667 | 2.64043333 |
| 2.07986667 | 2.64626667 |
| 2.0061 | 2.76776667 |
| 1.92373333 | 2.78106667 |
| 1.9678 | 2.571 |
| 2.0365 | 2.74203333 |
| 1.68533333 | 2.79733333 |
| 2.2894 | 2.73143333 |
| 2.19936667 | 2.67626667 |
| 1.59466667 | 2.59046667 |
| 2.6311 | 2.7865 |
| 2.2542 | 3.19963333 |
| 2.19686667 | 3.01883333 |
| 1.77263333 | 2.94166667 |
| 2.40606667 | 3.36866667 |
| 2.31143333 | 2.77178 |

Reference

1. Pfeiffer, F., Tolle, F., Rosenthal, M., Brandle, G.M., Ewers, J. et al. Identification and characterization of nucleobase-modified aptamers by click-SELEX. *Nat. Protoc.* **13**, 1153-1180 (2018).

2. Kacherovsky, N., Cardle, II, Cheng, E.L., Yu, J.L., Baldwin, M.L. et al. Traceless aptamer-mediated isolation of CD8(+) T cells for chimeric antigen receptor T-cell therapy. *Nat. Biomed. Eng.* **3**, 783-795 (2019).

3. Sylvestre, M., Saxby, C.P., Kacherovsky, N., Gustafson, H., Salipante, S.J. et al. Identification of a DNA Aptamer That Binds to Human Monocytes and Macrophages. *Bioconjug. Chem.* **31**, 1899-1907 (2020).

4. Guo, C.R., Zhang, Z.Z., Zhou, X., Sun, M.Y., Li, T.T. et al. Chronic cough relief by allosteric modulation of P2X3 without taste disturbance. *Nat. Commun.* **14**, 5844 (2023).

5. Liu, M., Hernandez-Morales, A., Clark, J., Le, T., Biswas, B. et al. Comparative genomics of Acinetobacter baumannii and therapeutic bacteriophages from a patient undergoing phage therapy. *Nat. Commun.* **13**, 3776 (2022).

6. Guo, L., Cheng, J., Lian, S., Liu, Q., Lu, Y. et al. Structural basis of amine odorant perception by a mammal olfactory receptor. *Nature* **618**, 193-200 (2023).

7. Broni, E., Striegel, A., Ashley, C., Sakyi, P.O., Peracha, S. et al. Molecular Docking and Dynamics Simulation Studies Predict Potential Anti-ADAR2 Inhibitors: Implications for the Treatment of Cancer, Neurological, Immunological and Infectious Diseases. *Int. J. Mol. Sci.* **24**, 6795 (2023).

8. Mukherjee, A., Hossain, Z., Erben, E., Ma, S., Choi, J.Y. et al. Identification of a small-molecule inhibitor that selectively blocks DNA-binding by Trypanosoma brucei replication protein A1. *Nat. Commun.* **14**, 4390 (2023).

9. Parkash, V., Kulkarni, Y., ter Beek, J., Shcherbakova, P.V., Kamerlin, S.C.L. et al. Structural consequence of the most frequently recurring cancer-associated substitution in DNA polymerase ε. *Nat. Commun.* **10**, 373 (2019).

10. Greco, F., Falanga, A.P., Terracciano, M., D'Ambrosio, C., Piccialli, G. et al. CD, UV, and In Silico Insights on the Effect of 1,3-Bis(1'-uracilyl)-2-propanone on Serum Albumin Structure. *Biomolecules* **12**, 1071 (2022).

11. Yan, Y., He, J., Feng, Y., Lin, P., Tao, H. et al. Challenges and opportunities of automated protein-protein docking: HDOCK server vs human predictions in CAPRI Rounds 38-46. *Proteins* **88**, 1055-1069 (2020).

12. Li, H., Huang, E., Zhang, Y., Huang, S.Y. & Xiao, Y. HDOCK update for modeling protein-RNA/DNA complex structures. *Protein Sci.* **31**, e4441 (2022).

13. Jumper, J. & Hassabis, D. Protein structure predictions to atomic accuracy with AlphaFold. *Nat. Methods* **19**, 11-12 (2022).

14. Akdel, M., Pires, D.E.V., Pardo, E.P., Janes, J., Zalevsky, A.O. et al. A structural biology community assessment of AlphaFold2 applications. *Nat. Struct. Mol. Biol.* **29**, 1056-1067 (2022).

15. Bryant, P., Pozzati, G. & Elofsson, A. Improved prediction of protein-protein interactions using AlphaFold2. *Nat. Commun.* **13**, 1265 (2022).

16. Salton, M., Kasprzak, W.K., Voss, T., Shapiro, B.A., Poulikakos, P.I. et al. Inhibition of vemurafenib-resistant melanoma by interference with pre-mRNA splicing. *Nat. Commun.* **6**, 7103 (2015).

17. Nikitin, M.P. Non-complementary strand commutation as a fundamental alternative for information processing by DNA and gene regulation. *Nat. Chem.* **15**, 70-82 (2023).

18. Wang, J., Mao, K., Zhao, Y., Zeng, C., Xiang, J. et al. Optimization of RNA 3D structure prediction using evolutionary restraints of nucleotide-nucleotide interactions from direct coupling analysis. *Nucleic Acids Res.* **45**, 6299-6309 (2017).
